# Characterization of the enhancer and promoter landscape of inflammatory bowel disease from human colon biopsies

**DOI:** 10.1038/s41467-018-03766-z

**Published:** 2018-04-25

**Authors:** Mette Boyd, Malte Thodberg, Morana Vitezic, Jette Bornholdt, Kristoffer Vitting-Seerup, Yun Chen, Mehmet Coskun, Yuan Li, Bobby Zhao Sheng Lo, Pia Klausen, Pawel Jan Schweiger, Anders Gorm Pedersen, Nicolas Rapin, Kerstin Skovgaard, Katja Dahlgaard, Robin Andersson, Thilde Bagger Terkelsen, Berit Lilje, Jesper Thorvald Troelsen, Andreas Munk Petersen, Kim Bak Jensen, Ismail Gögenur, Peter Thielsen, Jakob Benedict Seidelin, Ole Haagen Nielsen, Jacob Tveiten Bjerrum, Albin Sandelin

**Affiliations:** 1 Department of Biology, University of Copenhagen, 2200 Copenhagen N, Denmark; 2 Biotech Research and Innovation Centre, University of Copenhagen, 2200 Copenhagen N, Denmark; 3 Department of Gastroenterology, Medical Section, Herlev Hospital, University of Copenhagen, 2730 Herlev, Denmark; 4 Department of Gastroenterology, Surgical Section, Herlev Hospital, 2730 Herlev, Denmark; 5 DTU Bioinformatics, Technical University of Denmark, 2800 Lyngby, Denmark; 6 The Finsen Laboratory, Rigshospitalet, University of Copenhagen, 2200 Copenhagen N, Denmark; 7 Novo Nordisk Foundation Center for Stem Cell Biology, DanStem, University of Copenhagen, 2200 Copenhagen N, Denmark; 8 Department of Biotechnology and Biomedicine, Technical University of Denmark, 2800 Lyngby, Denmark; 9 Department of Science and Environment (INM), Roskilde University, 4000 Roskilde, Denmark; 10 Hvidovre Hospital, Gastrounit Medical Division, University of Copenhagen, 2650 Hvidovre, Denmark; 11 Hvidovre Hospital, Department of Clinical Microbiology, University of Copenhagen, 2650 Hvidovre, Denmark; 12 Centre for Surgical Science, Department of Surgery, Zealand University Hospital, 4600 Koege, Denmark

## Abstract

Inflammatory bowel disease (IBD) is a chronic intestinal disorder, with two main types: Crohn’s disease (CD) and ulcerative colitis (UC), whose molecular pathology is not well understood. The majority of IBD-associated SNPs are located in non-coding regions and are hard to characterize since regulatory regions in IBD are not known. Here we profile transcription start sites (TSSs) and enhancers in the descending colon of 94 IBD patients and controls. IBD-upregulated promoters and enhancers are highly enriched for IBD-associated SNPs and are bound by the same transcription factors. IBD-specific TSSs are associated to genes with roles in both inflammatory cascades and gut epithelia while TSSs distinguishing UC and CD are associated to gut epithelia functions. We find that as few as 35 TSSs can distinguish active CD, UC, and controls with 85% accuracy in an independent cohort. Our data constitute a foundation for understanding the molecular pathology, gene regulation, and genetics of IBD.

## Abstract (web summary)

Many SNPs associated with inflammatory bowel disease are located in non-coding genomic regions. Here, the authors perform CAGE-sequencing on descending colon biopsies of Crohn’s disease and ulcerative colitis patients to map transcription start sites and enhancer activity for analysis of regulatory regions.

## Introduction

**Fig. 1:**
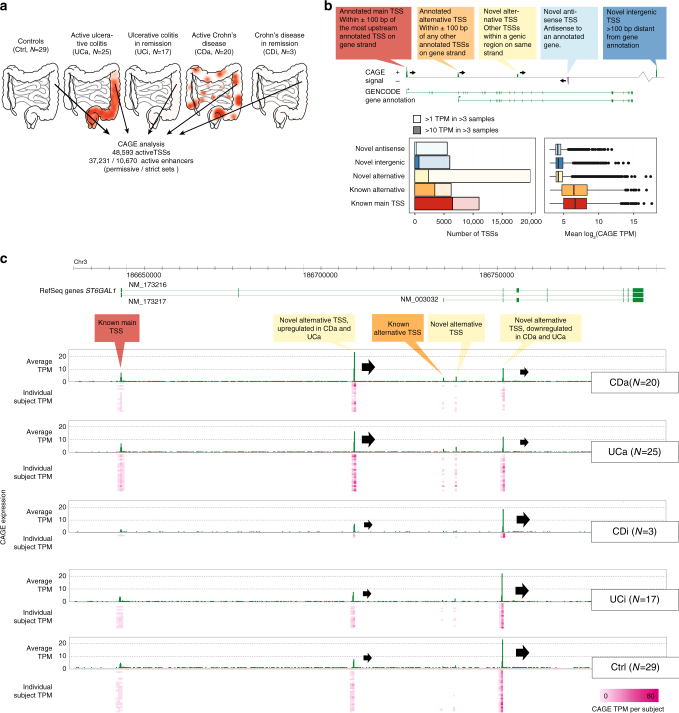
Defining the TSS landscape of IBD. **a** Overview of data set. Pinch biopsies from the descending colon were taken from 94 human subjects, classified into active ulcerative colitis (UCa), active Crohn’s disease (CDa), UC and CD patients in remission (UCi, CDi) and controls (Ctrl: subjects screened for IBD where all subsequent investigations turned out normal). For each biopsy, a CAGE library was produced, resulting in the detection of TSSs and enhancer regions. Schematics show the typical inflammatory patterns in the intestinal system, the approximate location of biopsy sampling and number of subjects in each group. **b** Detection and annotation of gene TSSs. Top panel shows an example gene with CAGE-defined TSSs, which are annotated as main, alternative or novel TSSs defined by their overlap with GENCODE gene annotation as indicated in callouts. CAGE-defined TSSs not falling into any of the categories were defined as novel intergenic TSSs. Left bottom panel shows the number of detected TSSs in each category (colors correspond to callouts in top panel), split by CAGE expression strength measured as tags per million (TPM). Right bottom panel shows the expression distribution of each category of TSSs as boxplots. **c** Genome-browser example of the detection of annotated and novel TSSs in the *ST6GAL1* gene. From top to bottom, the browser plot shows the genomic location investigated, RefSeq gene annotation (exons are denoted as boxes, green indicate forward strand transcription). Below, CAGE TPM expression on the forward strand is shown as average across subjects (green bars) and for individuals (pink heat map, each row is one subject, columns are widened 5× for readability), split by subject group. Annotated and novel TSSs, annotated as in **b** are highlighted. Note that the first novel alternative TSS is upregulated in CDa and UCa vs. remaining groups, while the last novel alternative TSS has the opposite pattern (block arrows indicate TSSs and their overall strength in each subject group for these two TSSs). Conversely, the annotated TSSs are detected but not substantially changing between groups

Inflammatory bowel disease (IBD) is an umbrella term for a range of chronic idiopathic disorders, of which Crohn’s disease (CD) and ulcerative colitis (UC) constitute the two major entities^1^, both with an increasing incidence and prevalence worldwide^2–4^ with an estimated 2.5–3 × 10^6^ patients in Europe alone^5^. UC is characterized by mucosal inflammation of the colon, whereas CD may affect all layers of the intestine throughout the gastrointestinal tract (Fig. 1a). The distinction between CD and UC is critical for correct medication and especially surgery, yet the diagnosis is challenging. In a previous meta-analysis of 22,038 IBD patients, it was impossible to distinguish UC or CD in 13% of cases^6^. This is a major problem for patients who have failed medical therapy and are facing colectomy. Genome-wide association studies (GWAS) have identified >200 loci containing IBD-associated variants^7,8^, but IBD-associated single-nucleotide polymorphisms (SNPs) can only explain 15–20% of the disease phenotype^9^. Moreover, ~70% of IBD-associated SNPs are non-coding^10^ and might affect gene regulation. However, a comprehensive map of active promoters and enhancers is lacking for IBD patients.

Genome-wide 5′-RNA sequencing of capped RNAs (Cap Analysis of Gene Expression, CAGE) can detect transcription start sites (TSSs) and thereby promoter regions^11^. Distal enhancer regions can also be detected by CAGE, because active enhancers transcribe enhancer RNAs (eRNAs)^12,13^. eRNA expression is a powerful proxy for cell-specific enhancer activity^14^, and CAGE-identified enhancers are two to three times more likely to validate in vitro than non-transcribed enhancers detected by chromatin-based methods^13^. An advantage of CAGE is that it can be easily applied on small biological samples, such as colonic biopsies that are routinely taken when diagnosing IBD.

Here we present CAGE analysis on biopsies from the descending colon from 94 IBD patients and controls. These data enabled annotation of IBD-regulated enhancers and TSSs, and characterization of IBD-associated SNPs in such regions. Furthermore, we define a small subset of TSSs that allow for accurate classification between UC, CD, and control subjects.

## Results

### The TSS expression landscape of IBD

We recruited 94 subjects undergoing lower endoscopy as part of routine visits, diagnosed as UC, CD, or control based on ref. ^15^. UC patients were graded by the Mayo score: a score ≤2 with endoscopic sub-score of 0 (no macroscopic inflammation) as quiescent UC and >2 as active UC. CD patients were graded by the Harvey–Bradshaw score: a score <5 as quiescent CD and ≥5 as active CD. For controls, an endoscopy was performed due to gastrointestinal symptoms but all clinical investigations returned normal. We extracted RNA from pinch biopsies from the descending colon of 94 subjects (cohort 1): 25 active UC (UCa), 20 active CD (CDa), 17 UC patients in remission (UCi), 3 CD patients in remission (CDi), and 29 control subjects (Ctrl) (Fig. 1a, Supplementary Table 1 and Supplementary Data 1). For UCa and CDa, biopsies taken from visually inflamed segments were confirmed by histology (Supplementary Figure 1). The choice of focusing on macroscopically inflamed rather than not visibly inflamed tissue was based on practical and statistical considerations: (i) in clinical diagnosis of IBD, histological examination is made on biopsies obtained from grossly inflamed intestine, together with endoscopical findings, medical history, laboratory, and imaging procedures. (ii) We wanted to characterize the most affected colonic regions of the disease to maximize comparability between biopsies, since non-macroscopically inflamed tissue might still show inflammation at the molecular level^16^.

For each subject, we prepared a CAGE library (Supplementary Data 2). We defined 48,593 expressed TSSs based on CAGE tag clusters (Supplementary Data 3). Most CAGE-defined TSSs were within gene loci: 22.6% (11,013) corresponded to annotated main TSSs from GENCODE models^17^, 12.8% (6229) to annotated alternative TSSs and 40.7% (19,787) TSSs were putative novel alternative TSSs within known gene loci, although the majority of these were lowly expressed (Fig. 1b). These may reflect spurious transcription initiation, TSSs expressed in rare cells or noise, but were not classified as bidirectional transcribed enhancers in our analysis (see below). The *ST6GAL1* gene, exemplifies the common occurrence of known and novel alternative TSSs in the same gene. The annotated main *ST6GAL1* TSS was detected but did not change between groups, while one novel alternative TSS was upregulated in IBD, and another was downregulated (Fig. 1c).

**Fig. 2:**
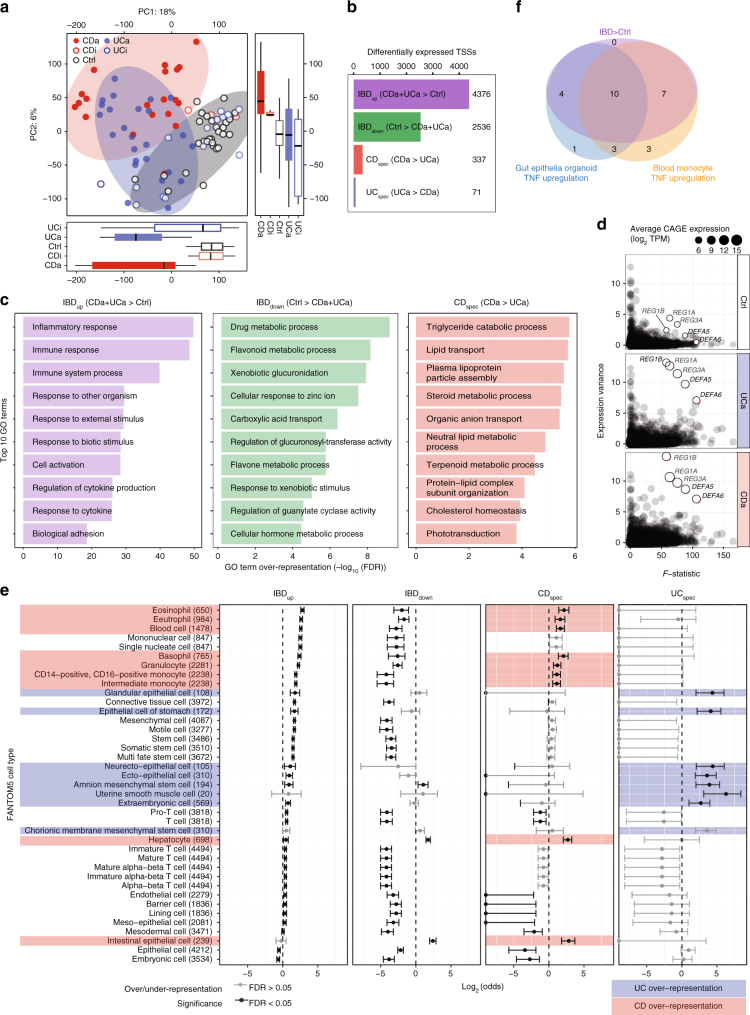
Differential expression of TSSs and genes in IBD. **a** Principal component analysis (PCA) based on CAGE TSS regions. *X*- and *Y*-axes show principal components (PCs) 1 and 2, percent of variance explained is indicated. Dots correspond to subjects, colored by group. Boxplots at the bottom and right show the distribution of PC. Circles show three major groups: CDa, UCa, and non-inflamed samples (UCi, CDi, and Ctrl). **b** Number of differentially expressed TSSs. Bar plot shows the number of differentially expressed TSSs in the four defined groups. **c** Gene ontology (GO) term overrepresentation analysis of differentially expressed genes. *X*-axis shows GO term overrepresentation FDR values on −log_10_ scale for differentially expressed genes in the IBD_up_, IBD_down_, and CD_spec_ sets. *Y*-axis shows the top 10 GO terms, ordered by FDR. **d** Identification of IBD-upregulated genes with extreme variance across IBD patients. *Y*-axis shows the variance of CAGE expression across CDa, UCa, and Ctrl subjects. *X*-axis shows the *F*-statistic from edgeR. Dots correspond to genes, where size indicates the average CAGE expression in respective group. Five outliers are highlighted, corresponding to antibacterial peptides. **e** FANTOM5 cell type enrichment of differentially expressed TSSs. Differentially expressed TSS sets were analyzed for overlap with TSSs specifically expressed in cell types in FANTOM5. *X*-axis shows under/over-representation of a given cell type expressed as log_2_(odds). Whiskers indicate 95% confidence intervals. Whiskers with black lines indicate statistical significance (Fisher’s exact test, FDR < 0.05). Cell types are ordered by their log-odds ratio in IBD_up_. Numbers in parentheses indicate the number of IBD-expressed TSSs specifically expressed in respective cell type in FANTOM5. Red shading shows cell types only enriched in CD_spec_ TSSs, blue indicates cell types only enriched in UC_spec_ TSSs. **f** Correspondence of TSSs upregulated in IBD with TSSs upregulated after TNF stimulation in epithelial organoids and blood monocytes. Venn diagram shows the number of TSSs (out of 36) upregulated in IBD (UCa or CDa vs. Ctrl), upregulated after TNF stimulation in gut epithelia organoids or blood monocytes, measured by qPCR. Upregulation after 4 and 24 h of TNF stimulation are pooled; see Supplementary Fig. 3c for time-specific measurements

CAGE TSSs principal component analysis (PCA) showed a separation between active IBD (UCa, CDa) and other groups (UCi, CDi, Ctrl), and a weaker separation between CDa and UCa (Fig. 2a). Surprisingly, UCi and CDi were not readily distinguishable from Ctrl (Supplementary Note 1). Hence, we excluded UCi and CDi from the systematic analysis below.

In order to identify differentially expressed TSSs, we used the generalized linear model framework in edgeR^18^. As observed above, CDa and UCa samples were characterized by a shared response compared to Ctrl (PC1 in Fig. 2a.), and secondly by differences between CDa and UCa (PC2 in Fig. 2a). To capture this, we defined sets of significantly up- or downregulated TSSs identified in both CDa and UCa vs. Ctrl (IBD_up_ and IBD_down_). To identify TSSs distinguishing CDa and UCa, we identified TSSs significantly upregulated in CDa vs. UCa, defined as CD_spec_, and TSSs significantly upregulated in UCa vs. CDa, defined as UC_spec_. The differential expression analysis recapitulated the PCA results: the number of TSSs within IBD_up_ (4376) and IBD_down_ (2536) was much larger than in CD_spec_ (337) and UC_spec_ (71) (Fig. 2b, Supplementary Data 4). The inclusion of additional patient data in the analysis, i.e. gender and previous medication, did not affect the number of differentially expressed TSSs substantially, indicating these effects are small compared to the CDa/UCa/Ctrl diagnosis (Supplementary Figure 2a). We also identified genes where one or more TSSs responded differently compared to the other TSSs inside the same gene (as exemplified in Fig. 1c): 2068 genes showed this pattern in IBD_up_/IBD_down_ and 82 in CD_spec_/UC_spec_ (Supplementary Data 5).

Because most functional annotations are on gene rather than TSS level, we defined differentially expressed genes by summing the contribution of each TSS within each GENCODE gene model and repeating the differential expression analysis above (Supplementary Data 6, Supplementary Fig. 2b). Gene Ontology (GO) analysis (Fig. 2c, Supplementary Data 7) of IBD_up_ genes showed a strong overrepresentation of GO terms related to inflammatory response and cytokines. IBD_up_ genes were also enriched for colon-specific processes previously related to IBD pathogenesis, for example remodeling of the extracellular matrix (FDR = 2.66e−09) and antibacterial peptide secretion (FDR = 1.02e−14). Because small networks of biologically linked genes are challenging to identify through GO analysis, we supplemented this analysis with STRING network analysis^19^. This identified smaller sets of upregulated genes with known functions in gut epithelia barrier integrity, including gap junctions (connexins *AQP5*, *GJA4*,*5*) and tight junctions (claudins *CLDN1*, *2*, *10*, *14*, and *18*) (Supplementary Fig. 2c). Interestingly, the full set of CLDN genes could distinguish IBD from Ctrl subjects, because a subset was part of the IBD_down_ set (Supplementary Fig. 2d−e). More generally, the IBD_down_ set was enriched for terms related to xenobiotic response and drug/steroid processing (Fig. 2c): STRING analysis showed that proteins corresponding to IBD_down_ genes were involved in cell cycle and growth, solute and membrane transport and maintenance of fluid balance in the intestine (Supplementary Fig. 2f), with a strong sub-network of genes involved in steroid hormone biosynthesis.

The CD_spec_ set was enriched for terms related to steroid, lipid, and lipoprotein metabolism (Fig. 2c). Because of the limited number of genes in the UC_spec_ set, only a handful of GO terms were overrepresented, including several terms linked to gap junctions. These results largely mirrored those identified by RNA-seq in ileal pediatric CD^20^. This study identified a CD-vs-UC upregulation of apolipoprotein genes, which was also present in our set (Supplementary Fig. 2g). Thus, it is clear that while CDa and UCa share a strong inflammatory component, we show that genes that differentiate CDa and UCa are not primarily related to immune response but rather to cellular functions associated to gut and gut epithelia.

Differential expression analysis will favor genes showing low variance within groups. However, gene expression variance across patients with the same diagnosis is relevant for patient stratification and precision medicine. We found that five antibacterial peptide genes, defensins *DEFA5* and *DEFA6* (normally expressed in small intestine^21^ and Paneth cells) and c-lectins *REG1A*, *REG1B*, and *REG3A* (normally expressed in pancreas and small intestine^21^) were highly upregulated in IBD and at the same time showed extreme variance across UC and CD subjects (Fig. 2d). The upregulation of these genes was highly correlated to cytokine induction (Supplementary Fig. 2h). The large variance might indicate patient-specific responses to inflammatory factors, disease chronicity or cell metaplasia, and again highlights the role of epithelia-specific genes IBD response.

Colonic biopsies consist of multiple cell types, whose composition may change in IBD. We assessed this in two ways. First, we took advantage of the FANTOM5 TSS atlas, where individual TSSs were annotated as being preferentially expressed in one or more cell types sampled across the human body based on CAGE expression^21^. With these data, we could estimate overall cell type enrichments for each differentially expressed TSS group across all FANTOM5 primary cell types (Fig. 2e, Supplementary Data 8). IBD_up_ TSSs were enriched in immune-related cells, while IBD_down_ TSSs were enriched in epithelial cells. Cell-type enrichment patterns of CD_spec_ and UC_spec_ TSSs were different: CD_spec_ TSSs were mostly enriched for immune-related cells (including basophils, monocytes, and eosinophils) and intestinal epithelial cells, while the UC_spec_ TSSs were mostly enriched in epithelial cells and mesenchymal cells. Although T-helper cells have previously suggested to define the difference between CDa and UCa (reviewed in ref. ^22^), they were not enriched in our analysis, consistent with previous results^23^.

Second, because FANTOM5 data only included cells from healthy tissue, we wanted to also assess whether induction of an inflammatory response induced IBD signature genes in immune cells and/or epithelial cells. To address this, we used primary cultures of colonic epithelia organoids^24^ and monocytes, which were stimulated with TNF (also known as TNFα), one of the most prominent inflammatory molecules associated with flaring disease in IBD patients^25^. Cells were stimulated with TNF for 4 and 24 h (Fig. 2f, Supplementary Fig. 3a, Supplementary Data 9) and the expression of 35 TSSs (established in the classification section below) was measured by qPCR. The same TSSs were measured by qPCR in gut biopsies from an independent cohort with 18 CDa, 37 UCa and 46 Ctrl subjects (cohort 2). We found that out of a total of 21 TSSs that were upregulated in UCa or CDa vs. control, 66% (14/21) and 81% (17/21) were also upregulated in TNF-stimulated organoids and monocytes, respectively, at any time point; 47% (10) of TSS were upregulated in both stimulated organoids and monocytes (Fig. 2f and Supplementary Fig. 3b, c). It is thus unlikely that the IBD expression change is solely attributed to immune cells; epithelial cells are likely part of shaping the transcriptional changes associated with UC and CD.

Thus, in agreement with the GO analysis, these cell type enrichment results indicated that the difference between Ctrl, CDa, and UCa does not only lie in immune cells and their associated general immune response pathways, but also in cells and genes associated with gut epithelia.

### The enhancer landscape of inflammatory bowel disease

**Fig. 3:**
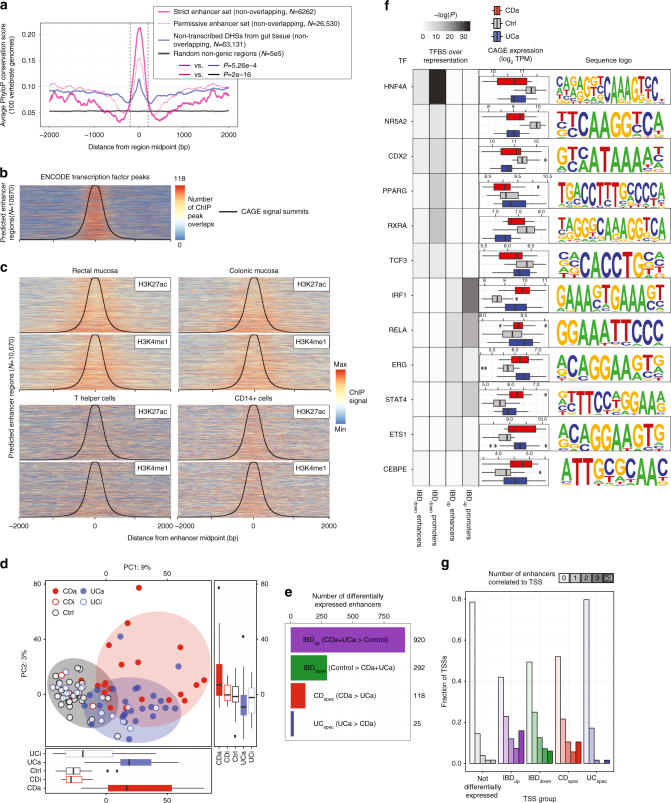
Discovery and characterization of enhancer activity in IBD **a**. Conservation analysis of enhancer regions. *X*-axis shows the distance from the center of regions. *Y*-axis shows average PhyloP100 vertebrate conservation score^65^ for strict and permissive enhancer sets, non-transcribed DHSs from gut^27^ and random non-genic regions. *P*-values indicate Mann–Whitney *U* tests between conservation scores in the ±200 bp region (dashed lines). Number of regions in each set are shown (overlapping regions were discarded). **b** Transcription factor binding enrichment within CAGE-defined enhancer regions. Heat map rows correspond to the 10,670 enhancer predictions, sorted by distance between bidirectional CAGE peaks. *X*-axis corresponds to the ±2000 bp region centered on enhancer midpoints. CAGE peak summits are shown as black lines. Color intensity corresponds to number of ENCODE transcription factor ChIP-seq peaks (all ENCODE cells) overlapping a given region. **c** H3K27ac and H3K4me1 ChIP-seq enrichment within enhancer regions identified by CAGE in gut biopsies. Heat maps are constructed as in **b**, but show ChIP-seq signal from H3K27ac and H3K4me1 from rectal/colonic mucosa, T helper and CD14+ cells^27^. Colors are assigned based on observed min and max ChIP-seq intensity values within each heatmap. **d** Principal component analysis based on CAGE expression within enhancer regions. Plot is organized as Fig. 2a. **e** Number of differentially expressed enhancers. Bar plot shows the number of differentially expressed enhancers per group, organized as Fig. 2b. **f** Predicted transcription factor site enrichment in enhancer and TSS regions. Each row shows data related to one transcription factor. Left panel shows site enrichment *P* value in respective groups of differentially expressed enhancers or promoters for the sites corresponding to the relevant motif, as indicated by color scale. Middle panel shows the CAGE expression as log_2_ TPM for the transcription factor across groups as boxplots. Right panel shows motif sequence logo. **g** Linkage between enhancer and TSS through co-expression. *Y*-axis shows the fraction of CAGE TSSs that can be linked to enhancers within 500 kb through co-expression correlation, split by how many enhancers each TSS is linked to. *X*-axis shows sets of TSSs split by their differential expression as in Fig. 2b

Gene and TSS-focused analysis as above can give insights into gene expression but not their regulation by distal enhancers. As established previously, bidirectional CAGE peaks accurately predict enhancer locations and activity^13^. Using the same method as in ref. ^13^, we predicted a permissive set of 37,231 enhancers, and a strict subset of 10,670 enhancers where ≥8 samples had detectable enhancer expression (the latter set was used in the rest of the analyses; Supplementary Data 10, 11 and Supplementary Fig. 4a). The strict enhancer set overlapped ENCODE DNase hypersensitive sites (DHSs)^26^ in 90% of cases (*P* < 2e−16, by sampling), and these enhancers were significantly more conserved across mammals than non-transcribed DHSs from gut tissue^27^ and randomly selected genomic regions (*P* < 5.26e−4, two-sided Mann–Whitney *U* test; Fig. 3a). These enhancers were also strongly enriched for ENCODE transcription factor (TF) ChIP-seq peaks from diverse cells (Fig. 3b). Similarly, they were highly enriched for H3K27ac and H3K4me1 ChIP-seq signals from rectal and colonic mucosa, and to a lesser degree other intestinal regions^27^ (Fig. 3c and Supplementary Fig. 4b). FAIRE-seq signals from colonic CD and control patients^28,29^ were also enriched (Supplementary Fig. 4c). However, the regions had less H3K27ac and H3K4me1 ChIP-seq enrichment in immune cells and non-gut tissues (Fig. 3c and Supplementary Fig. 4b). Thus, the enhancer candidates had the hallmarks of enhancer regions in general, but seemed most strongly used in gut tissue.

PCA of enhancer expression showed separation between inflamed samples (UCa and CDa) and Ctrl, but only minor separation between UCa and CDa (Fig. 3d). We used EBSeq^30^ to define four differentially expressed enhancer groups, analogous to the TSS groups defined above: shared CDa and UCa up/downregulated vs. Ctrl (IBD_up_ and IBD_down_), upregulated in CDa vs. UCa (CD_spec_) and vice versa (UC_spec_) (Supplementary Data 12 and Supplementary Fig. 4d). In agreement with the PCA, the IBD_up_, and IBD_down_ enhancer sets were much larger than CD_spec_ and UC_spec_ sets (Fig. 3e).

Next, we used HOMER^31^ to identify shared predicted TF binding sites around IBD_up_ and IBD_down_ enhancers (±300 bp around enhancer midpoints; CD_spec_ and UC_spec_ sets were too small for meaningful analysis). The same analysis was used for promoter regions of corresponding TSS groups (defined as −500/+100 bp around TSSs) (Fig. 3f). IBD_up_ promoters and IBD_up_ enhancers were enriched for sites linked to TFs previously associated with inflammation, including ETS1, IRF1, STAT4, and RELA. IBD_down_ promoters and enhancers site enrichments were also highly similar: in particular, HNF4A and CDX2 sites were over-represented, consistent with their important roles in differentiation of gut epithelial cells^32^. Decreased *CDX2* and *HNF4A* expression were previously observed in IBD^33,34^, and indeed, site enrichments generally correlated with the expression of the associated TF (Fig. 3f). We observed similar enrichment patterns when analyzing enhancer and promoter regions for overlapping ENCODE TF ChIP-seq peaks (Supplementary Fig. 5). Thus, IBD-induced enhancers and promoters share similar TF binding pattern enrichments, which are distinct from those of IBD-repressed enhancers and promoters.

To understand enhancer regulation, enhancers must be linked to target gene TSSs. As shown previously^13^, enhancer-TSS pairs can be predicted based on CAGE co-expression across samples. Following the same approach, we identified 21,502 enhancer-TSS pairs within 500 kb with positive and significant Pearson correlation (correlation test, FDR < 0.05) (Supplementary Data 13). Of these pairs, 8507 (39.6%) were between a differentially expressed TSS and an enhancer, regardless of its differential expression status. Around half of IBD_up_ and IBD_down_ TSSs (54.7% and 47.9%, respectively) were linked with at least one enhancer, compared to smaller fractions for CD_spec_, UC_spec_ (40.9% and 18.3%) and remaining TSSs (Fig. 3g). Enhancer-linked TSSs in IBD_up_ and IBD_down_ groups were commonly linked with more than one enhancer: in particular, IBD_up_ TSSs linked to >3 enhancers were as frequently observed as TSSs linked to a single enhancer (Fig. 3g).

**Fig. 4:**
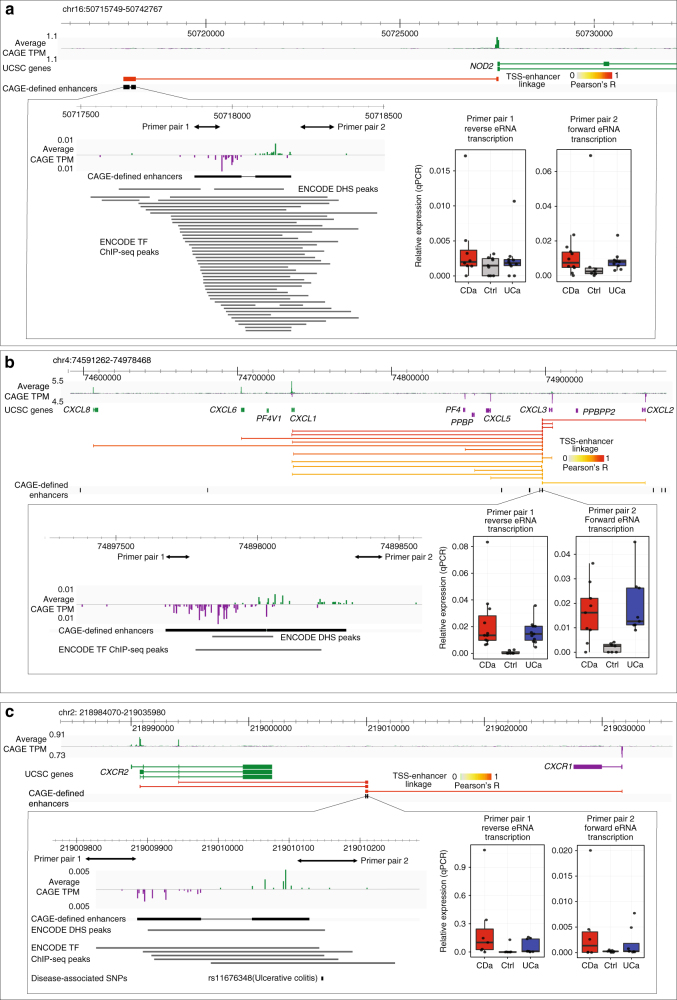
Examples of IBD-upregulated enhancers and linked TSS and genes. Each larger panel shows: genome browser with the average CAGE expression in TPM on both strands, UCSC gene models, TSS-enhancer-linkage through expression correlations (Pearson correlation coefficients are indicated by color, only positive correlations are shown) and CAGE-defined enhancers (black). Green indicates data on forward strand, purple on the reverse strand. Left part of lower panel shows a zoom-in of the enhancer region, with CAGE signal intensity as above, ENCODE DHS peaks and TF ChIP-seq peaks^26,66^. ChIP-seq peaks are labeled with cognate TF name. Primer locations for qPCR analysis are indicated as double arrows. Right lower panel shows corresponding qPCR analysis of eRNA expression in CDa, UCa and Ctrl samples on both strands as boxplots, relative to the *PIAS4* reference gene. **a** An enhancer upstream of the *NOD2* TSS. An enhancer region was detected upstream of the TSS of *NOD2*, a key gene in IBD pathogenesis with strong support from ENCODE cell line data. **b** An enhancer in the *CXCL1-3*,*5*,*6*,*8* cytokine cluster. Several enhancer regions within a cluster of chemokine genes (*CXCL1-3*, *CXCL5-6*, and *CXCL8*, all upregulated in IBD) were detected. The analysis is focused on a single enhancer linked to the above gene TSSs (for ease of visualization, only links between this enhancer and TSSs are shown). **c** An enhancer between *CXCR1* and *CXCR2*. An enhancer between *CXCR1* and *CXCR2* (receptors for the cytokines in panel **b**) was detected. This enhancer overlapped multiple ENCODE TF ChIP-seq peaks, and the UC-associated rs11676348 SNP. Note the two alternative TSSs for *CXCR2*. In the lower panel, a track with disease-associated SNPs is shown

The *NOD2* locus, a cluster of chemokine genes (*CXCL1-3*, *CXCL5-6*, and *CXCL8*), and two receptors for these chemokines (*CXCR1* and *CXCR2*), represent examples of enhancers linked with neighboring TSSs of genes associated with IBD pathogenesis (Fig. 4a–c). The examples show putative distal regulators for key IBD genes, and highlighted a potential regulatory SNP that may affect the chemokine response pathway. Due to the common occurrence of enhancers linked to cytokine-related genes, we annotated the cytokine–cytokine receptor interaction pathway with linked enhancers and alternative TSSs (Supplementary Fig. 6).

### Characterization of IBD-upregulated enhancer clusters

**Fig. 5:**
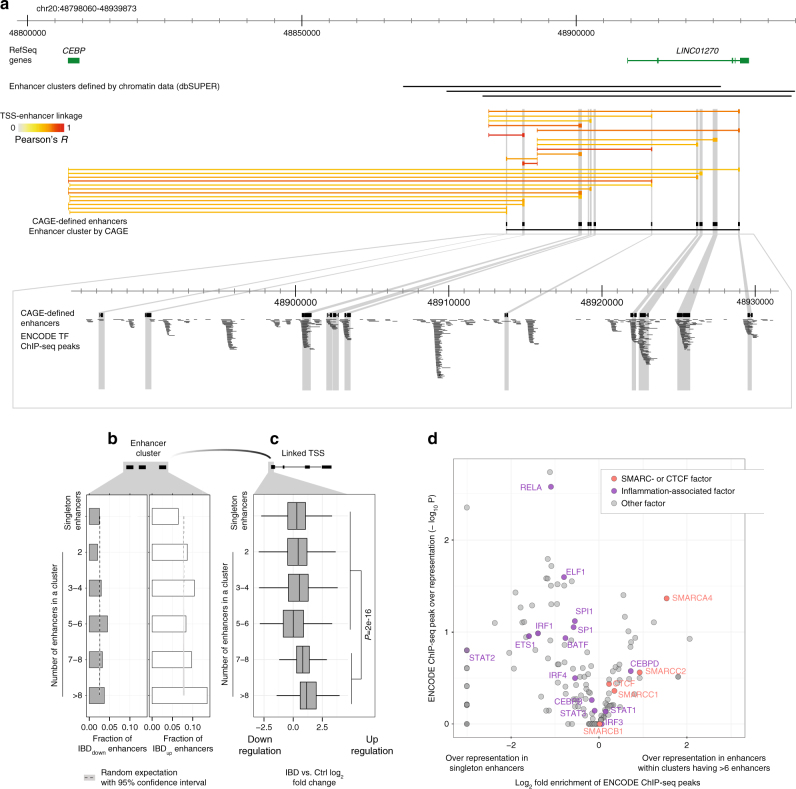
Characterization of enhancer clusters in IBD. **a** Example of an enhancer cluster in the *CEBPB* locus. Genome browser screenshot of the *CEBPB* locus, organized as in **a** but also showing chromatin-defined enhancer clusters from dbSUPER^36^, and a CAGE derived enhancer cluster located ~150 kb downstream of *CEBPB*. Because *CEBPB* has two nearby alternative TSSs with similar activity, most enhancers are linked to both. Lower panel shows a zoom-in of the enhancer cluster where ENCODE transcription factor ChIP-seq peaks are displayed: each black line corresponds to one ChIP-seq peak. **b** Relation between enhancer IBD-up/downregulation and number of enhancers within an enhancer cluster. Bar plot shows the fraction of enhancers that are significantly downregulated (IBD_down_, gray) or upregulated (IBD_up_, white) in UCa and CDa vs. Ctrl, grouped by the number of enhancers within an enhancer cluster. Enhancers not part of clusters are included for comparisons (singleton enhancers). The expected overlap by chance for each bar is indicated as dotted lines, with 95% confidence intervals. **c** Relation between the number of enhancers within an enhancer cluster and IBD upregulation of linked TSSs. Boxplots show the distribution of IBD vs. Ctrl log_2_ fold changes of TSSs linked to singleton enhancers or enhancer clusters as in **b**. TSSs are grouped by how many enhancers the linked enhancer cluster contains. **d** Overrepresentation of ENCODE TF ChIP-seq peaks in singleton enhancers vs. enhancer clusters with >6 members linked to IBD-upregulated TSSs. *X*-axis shows the log_2_ fold change in ENCODE ChIP-seq peak over-representation in single enhancers vs. enhancers within enhancer clusters having >6 enhancers, where 0 indicates no difference between sets. Only enhancers linked to IBD_up_ TSSs are analyzed. *Y*-axis shows the associated over-representation *P*-value. Each dot corresponds to one type of ChIP-seq peak, colored by whether they are annotated as inflammation-associated factors (purple), SMARC- or CTCF factors (orange), or other factors (gray). Factors of interest are highlighted

Previously, large regions having enhancer-like chromatin features—so-called “super”—or “stretch”—enhancers—were identified as drivers of central biological processes^35^. By eye, clusters of CAGE-defined enhancers were evident, for instance in the region surrounding *CEBPB*, a key inflammatory response TF (Fig. 5a). We defined CAGE-based enhancer clusters by requiring >2 consecutive enhancer midpoints spaced ≤15 kb from each other, resulting in a set of 693 enhancer clusters (Supplementary Data 14), where 67% overlapped enhancer clusters defined by ChIP-seq in immune and gut cells^36^. CAGE-defined enhancer regions within clusters often co-occurred with ENCODE TF ChIP-seq peaks (Fig. 5a).

The number of CAGE-defined enhancers within a cluster correlated with the average fraction of IBD_up_, but not IBD_down_ enhancers in the same cluster (Fig. 5b). The same trend was observed for predicted enhancer targets: TSSs linked to at least one enhancer within a cluster had a higher IBD vs. Ctrl fold change if the enhancer cluster contained >6 enhancers compared to TSSs linked to clusters with fewer enhancers (*P* < 2.2e−16, two-sided *t*-test) (Fig. 5c and Supplementary Fig. 7a). Consistent with these observations, if an IBD_up_ TSS was linked to an enhancer in a cluster with >6 enhancers, it was typically linked with all or most enhancers within the cluster (Supplementary Fig. 7b). Altogether, this suggests that enhancer clusters with many members and singleton enhancers have distinct functions in IBD.

To further explore the relation between the number of enhancers within a cluster and their regulatory function in IBD, we compared (i) IBD_up_ TSS-linked enhancers from enhancer clusters with >6 members with (ii) IBD_up_ TSS-linked singleton enhancers (not belonging to any cluster). Singleton enhancers had a greater overlap with ENCODE ChIP-seq peaks corresponding to TFs associated with inflammation (including RELA, SP1, STAT factors, and IRF1) (Fig. 5d), while enhancer clusters were overrepresented for ChIP-seq peaks for chromatin remodelers and insulators, including SMARCA4 and CTCF. As SMARCA4 has a function in activation of repressed genomic regions^37^ and CTCF is an insulator, these larger enhancer clusters might reflect large-scale changes in chromatin structure induced by the inflammatory response, while smaller enhancer clusters may be driven by the binding of one or a few TFs.

### SNP overrepresentation in enhancers and promoters

As discussed above, a majority of IBD-associated SNPs are located in intergenic regions, and may impact gene regulation. The UC-associated SNP overlapping an IBD_up_ enhancer located between cytokine receptors *CXCR1* and *CXCR2* (Fig. 4c) exemplifies an SNP with potential regulatory function. As we did not have access to the genotypes of our subjects, we investigated the overlap between publically available IBD-associated SNPs and enhancer and promoter regions using two complementary approaches; GWAS SNP enrichment and partitioned heritability of IBD.

First, we obtained all SNPs significantly associated with a disease/trait from the GWAS catalog^38^. For each GWAS SNP, we merged the region covered by other SNPs in linkage disequilibrium (LD, *R*^2^ > 0.75 and within 500 kb) into a single block, using the 1000 genomes CEU reference population^39^. If LD blocks overlapped, they were merged into an LD clump.

**Fig. 6:**
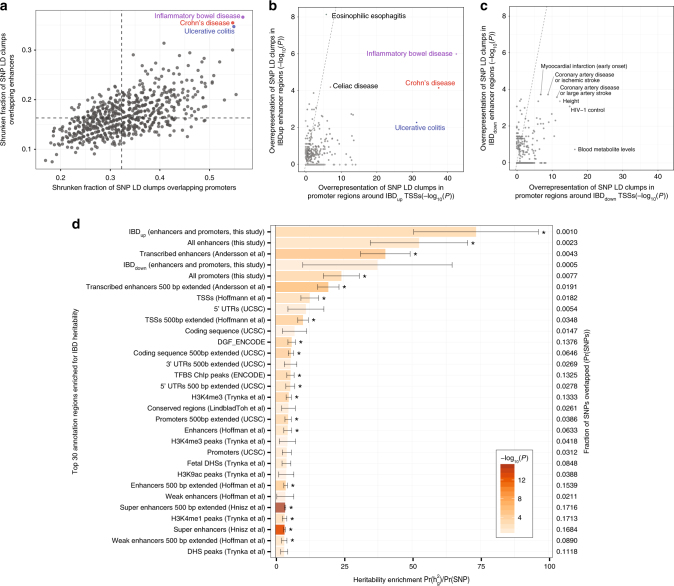
Relation between IBD-associated SNPS and IBD-induced regulatory regions. **a** Overlap of GWAS catalog diseases and traits with identified TSSs and enhancers. *Y*-axis shows the fraction of SNP LD-clumps associated to each GWAS catalog disease/trait that overlaps enhancer regions identified in this study. *X*-axis shows the same statistic for promoter regions, defined from CAGE TSSs identified in this study. Fractions are shrunk towards the mean across all diseases/traits (dashed lines) using empirical Bayes. LD clumps associated to CD, UC, and IBD show the highest degree of overlap with both promoter and enhancer sets. **b** Enrichment of IBD_up_ promoters and enhancers for GWAS catalog diseases/traits. *Y*-axis shows the overrepresentation of LD-clumps linked to GWAS catalog diseases/traits in IBD_up_ enhancers vs. all enhancers expressed as Fisher’s Exact test *P* values. *X*-axis shows the same statistic for promoter regions corresponding to IBD_up_ TSS vs. all TSSs. Each dot represents one GWAS catalog disease/trait. UC, CD, and IBD are the only shared enrichments between the two sets. **c** Enrichment of GWAS catalog diseases and traits for IBD_down_ promoters and enhancers. Plot is organized as in **b**, but showing LD-clump enrichment of IBD_down_ enhancers (*Y*-axis) and promoters corresponding to IBD_down_ TSSs (*X*-axis). **d** Partitioned heritability of IBD for different classes of genomic regions. Bar plots show the heritability enrichment of IBD, as estimated by stratified LD-score regression for each category, expressed as $\Pr \left( {h_g^2} \right)/{\mathrm {Pr}}\left( \mathrm {SNPs} \right)$ in the respective regions^40^. Whiskers show jackknife standard errors of the heritability enrichment. *Y-*axis shows the top enriched genome regions, defined in ref. ^40^, supplemented by regions defined in this study as indicated, sorted by enrichment score. Bar color indicates significance of enrichment in log_10_ scale; asterisks indicate FDR < 0.05. Percentages to the right indicate the fraction of the genome covered by each set of regions $\left( {\mathrm {Pr}\left( \mathrm {SNPs} \right)} \right)$

For every GWAS disease, we assessed the overlap between LD clumps with our identified promoter (−500/+100 bp around TSS peaks) and enhancer regions (±300 bp around enhancer midpoints) by using empirical Bayes to shrink fraction of overlapping LD clumps toward the average of all GWAS diseases. Only IBD, CD and UC LD clumps had a high degree of overlap with both the promoter and enhancer sets (Fig. 6a and Supplementary Fig. 8a). Next, we analyzed whether differentially expressed TSSs and enhancers were enriched for LD clump overlaps. IBD_up_ promoters and enhancers were enriched for LD clumps associated with several immune-related diseases, but only IBD, CD, and UC LD clumps were enriched in both promoters and enhancers (Fig. 6b). Enhancers, but not promoters, were highly enriched for other inflammatory intestinal disease LD clumps. Conversely, IBD_down_ TSSs and enhancers were not enriched for LD clumps associated with IBD, UC, or CD, but showed modest enrichment for LD clumps associated with heart disease (Fig. 6c).

The above analysis only assessed enrichments of genetic variants reaching genome-wide significance. However, many genetic variants might collectively make important contributions to IBD pathogenesis without individually reaching genome-wide significance. To this end, we performed partitioning of heritability analysis using the stratified LD-score regression method^40^ to investigate if TSSs and enhancers were enriched for SNPs explaining the heritability of IBD. Briefly, this method measures SNP heritability enrichment in a set of genomic regions (e.g. enhancers) as the proportion of SNP heritability $\left( {{\mathrm{Pr}}\left( {h_g^2} \right)} \right)$ divided by the proportion of SNPs $\left( {{\mathrm{Pr}}\left( {\mathrm {SNP}} \right)} \right)$ in the same regions, based on GWAS summary statistics. Previously, this method was employed on 24 classes of annotated functional regions and GWAS summary statistics from 17 complex diseases, to establish a “baseline model” describing the contribution of each annotation class^40^. To extend this analysis with our enhancer and TSS sets in an IBD setting, we obtained GWAS summary statistics for ~12 million SNPs from the International IBD Genetics Consortium^8^. We then analyzed the partitioned heritability of IBD in the 24 annotated functional regions from^40^ but added our enhancer (±300 bp around enhancer midpoints) and promoter (−500/+100 bp around TSS peaks) regions, and the subsets of these belonging to the IBD_up_ or IBD_down_ groups, to the original model. We found that our enhancers and promoter sets were significantly enriched for IBD SNP heritability. This was even more evident in the IBD_up_ enhancers and promoters, while IBD_down_ was not significantly enriched (Fig. 6d, Supplementary Fig. 8b, c). The combined IBD_up_ enhancer and promoter set had the highest heritability enrichment of all regions included in the baseline model, including FANTOM5 enhancers. Overall, IBD heritability enrichment was considerably higher for annotation classes corresponding to regulatory regions (enhancers, promoters etc.) than coding exons.

Overall, these results suggest that there is an association between IBD-associated SNPs and the regulatory regions we define. While the genotypes of the samples investigated here would be necessary to establish the causal effect of individual SNPs, our analysis indicate that the enhancer and promoter resources we provide are useful starting points for future investigations of non-coding IBD-associated SNPs, since they allow for a more detailed interpretation of otherwise uncharacterized intergenic regions.

### Classification of UC, CD, and controls from TSS expression

The statistical analyses above could identify TSSs and enhancers that were up- or downregulated between subject groups, but cannot assess whether expression data for a single biopsy contain enough information to accurately classify the subject as active UC, active CD, or control. This is a relevant question as the diagnosis between active UC and CD is often clinically difficult, and expression data might be useful to increase overall diagnosis accuracy. We reasoned that CAGE-based analysis is not realistic in a clinical setting; instead, we sought to define a small set of CAGE-based biomarkers for active UC, active CD and controls that were amendable to robust targeted methods such as qPCR.

**Fig. 7:**
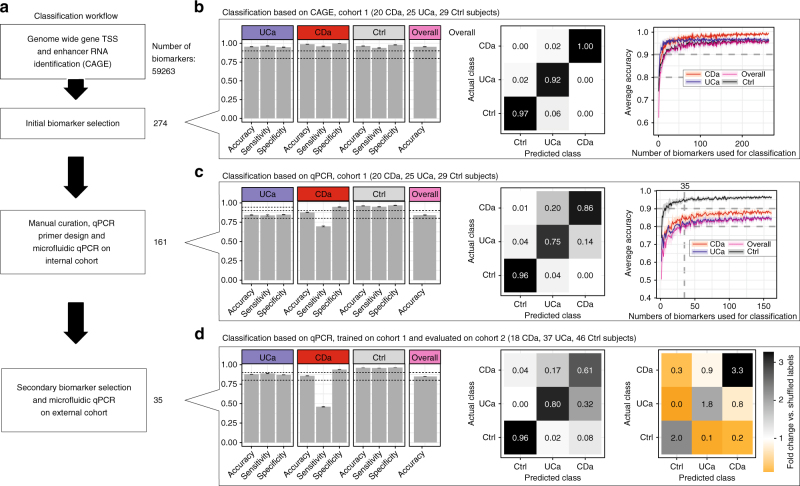
Classification of UC, CD, and controls. **a** Overview of analyses. Starting from all TSSs and enhancers (referred to as biomarkers, *N* = 59,263) in cohort 1, we performed an initial feature selection using an ensemble approach, resulting in 274 features. We designed successful qPCR primer pairs for 161 biomarkers and applied microfluidic qPCR analysis to the same samples. A secondary feature selection process was used to reduce the set of biomarkers to 35. We analyzed the expression of these biomarkers in an independent validation cohort (cohort 2) using microfluidic qPCR. Classification analysis was performed at each step (panels **b**–**d**). **b** Prediction of UCa/CDa/Ctrl diagnosis labels based on CAGE expression. CAGE expression data from cohort 1 from 274 selected biomarkers were used to train and evaluate a Random Forest model based on five-fold cross-validation 1000 times. Left panel: average accuracy, sensitivity, and specificity are shown for each subject group as bar plots along with overall accuracy. Error bars show 95% confidence intervals across cross-validations. Dotted lines indicate 0.8 and 0.9. Middle panel: confusion matrix showing average fractions of predictions that fall into each of the actual subject groups (columns add to 100%). Right panel: average prediction accuracy (*Y*-axis) as a function of number of biomarkers used for training (*X*-axis). Shaded areas indicate 95% confidence intervals across cross-validations. **c** Prediction of UCa/CDa/Ctrl diagnosis labels based on microfluidic qPCR expression. Plots are organized as in panel **b**, but based on microfluidic qPCR expression data from cohort 1 using 161 primers corresponding to selected biomarkers. **d** Validation using an independent cohort feature reduction based on the data in panel **c** resulted in the selection of 35 features. We trained a Random Forest model on microfluidic qPCR data from these biomarkers from cohort 1 and evaluated it on corresponding data from an independent cohort (cohort 2). Left and middle panels show classification results, as in panel **b**. Right panel shows a comparison between the confusion matrix (as in panel **b**) of our predictions and the confusion matrix obtained by repeating the analysis with randomly shuffled training labels. Numbers indicate the average fold changes of fractions (actual vs. shuffled)

To achieve this, we used successive steps of biomarker selection and testing (Fig. 7a). First, we analyzed the CAGE data from the 25 UCa, 20 CDa, and 29 Ctrl subjects in cohort 1 with an ensemble of statistical and manual curation methods to extract 274 TSSs/enhancers distinguishing UCa, CDa, and Ctrl groups (Supplementary Data 15). A Random Forrest (RF) classification framework trained on CAGE data for these 274 biomarkers could predict UCa, CDa, and Ctrl diagnosis with an overall accuracy of 95%, assessed by five-fold cross-validation (Fig. 7b, left and middle panels). This high accuracy could be retained when reducing the number of biomarkers (Fig. 7b, right panel). We were able to design and validate qPCR primers corresponding to 161 of these biomarkers (Supplementary Data 15, 16), and analyzed their expression using microfluidic qPCR on the same biopsies as above (cohort 1: 161 primer pairs analyzed in biopsies from 74 subjects) (Supplementary Data 17). An RF trained on the microfluidic qPCR expression data gave an overall accuracy of 84% assessed by five-fold cross-validation (Fig. 7c, left and middle panels). Thus, accurate classification of IBD based on these biomarkers was achievable across experimental methods. Similar accuracy levels were achieved using fewer biomarkers: we retained comparable accuracy using 30–40 biomarkers (Fig. 7c, right panel). Encouraged by this, we reduced the number of biomarkers to a final set of 35. In this final selection no enhancers were retained, consistent with their lower RNA abundance compared to gene TSS.

To test whether the classification power of our biomarkers generalized beyond cohort 1, we enrolled a second independent validation cohort (cohort 2: 18 CDa, 46 Ctrl, 37 UCa; Supplementary Table 1), which included biopsies acquired at two different hospitals by different physicians, and measured the expression of same 35 biomarkers using microfluidic qPCR (Supplementary Data 18). We then trained a RF on microfluidic qPCR data from the 35 biomarkers from cohort 1 and predicted the diagnosis of the subjects in cohort 2. We achieved an overall accuracy of 85% in cohort 2 (Fig. 7d, left and middle panels), strongly suggesting that our selected biomarkers generalize to the larger population. Although the CD sensitivity was lower when using qPCR than with CAGE (see Discussion), our results were in all cases substantially better than expected by chance, as estimated by training on randomly shuffled cohort 1 labels and then predicting the labels of cohort 2 (Fig. 7d, right panel and Supplementary Figure 9a, b). To explore whether CD sensitivity could be improved further, we employed the gradient boosting decision tree method implemented in XGBoost^41^, training on the same 35 biomarkers cohort 1 and predicted the diagnosis of the patients in cohort 2. This resulted in an increase in CDa classification sensitivity by 13% points with no overall classification performance decrease (Supplementary Figure 9c). Thus, our initial results are not specific to machine learning method employed and may be improved by the use of more complex techniques. In summary, we have shown that a small set of qPCR primers, selected from CAGE data, could distinguish control, active UC, and active CD biopsies.

## Discussion

Here we have profiled the enhancer and TSS landscape of biopsies taken in the descending colon of admitted IBD patients and controls. Such biopsies are identical to biopsies used in current diagnosis methods. This has the advantage that samples are highly clinically relevant, and that transcriptional changes in the disease states measured will be close to the in vivo reality. Thus, from a diagnostic perspective, such tissue samples are more relevant than selected primary cells isolated from tissue. As with all tissue samples, expression changes may be partially due to changes in cell composition. We showed that aside from a shared increase of immune cell-linked TSSs, active CD and UC seem to have different composition of cells, where UC-specific gene expression was more strongly linked to epithelial cells.

CAGE data interpreted on gene level identified similar pathway and GO term enrichments as previous RNA-seq studies^20,28,42^. A recent study^28^ identified two distinct CD populations based on RNA expression data: we could not replicate this finding but the CAGE data did show the active CD population to be much more transcriptionally diverse than active UC or controls, as discussed below.

This is a large-scale analysis of enhancer activities across a total of 94 CD, UC, and control subjects: ref. ^28^ investigated two smaller CD populations (*N* = 10 and 9) using FAIRE-seq, a method identifying accessible DNA, while H3K27ac ChIP-seq has been performed in gut tissue for even fewer individuals, and not in an IBD context^27,43^. With our data, we could establish that promoters and enhancers that are upregulated in IBD share the same DNA-binding patterns enrichments, and that enhancer clusters upregulated in IBD are distinct from corresponding singleton enhancers.

The enhancer component of IBD reported here gives new possibilities for understanding the disease and its genetics. We show a clear overrepresentation of IBD-associated SNPs in both IBD-upregulated enhancer and promoter regions, and these regions had the largest IBD heritability enrichment compared to a large set of other genomic regions. Thus, the IBD TSS and enhancer sets reported here are unique resources for interpretation of the functional impact of noncoding genetic variants.

Aside from adding to the functional and genetic understanding of IBD, CAGE data can be used to distinguish between active UC, CD, and control subjects. This accuracy could be retained using as few as 35 TSSs, quantified by microfluidic qPCR and validated in an independent cohort. Although the method is not at usable in clinical settings in its current form, it may provide an inroad for new qPCR-based diagnostic methods complementing current diagnostic methods. Several studies have used microarray- or qPCR-based approaches to classify UC, CD and controls previously (e.g.^16,23,44^), with varying degrees of success ranging from high separation in small cohorts to little or no separation: a meta-study analyzing multiple microarray experiments indicated that UC-CD differences are not substantial^23^, and the accuracy of one of the reportedly best-performing methods could not be verified in an independent laboratory^45^. These variations in accuracy may be due to small patient cohorts, and that different standards may have been applied in the initial patient diagnosis and inclusion criteria. For comparison, we tested the classifying power of gene expression markers previously selected by two previous studies^16,44^ in our set, using CAGE data in cohort 1. These markers performed consistently worse than ours (Supplementary Fig. 9d).

There are two important limitations with our analysis. First, biopsies were collected from inflamed segments of the descending colon only; expression patterns may be different in other parts of the intestinal system, and while UCa patients typically are inflamed in the descending colon, this is less common for CDa patients. Similarly, it is possible that non-macroscopically inflamed biopsies from IBD patients have better classification potential, but on the other hand macroscopically inflamed biopsies are the primary type of samples used for histology assessment in the clinical diagnosis of IBD and it is clear that IBD-upregulated regions we identified in such biopsies were highly overrepresented in SNPs associated to the disease. The latter argues that the regions identified are disease-relevant and involved in pathogenesis. Second, the sensitivity of CDa prediction was lower than that of UCa and Ctrl. This may be related to the above, but as we noticed a much higher variance among CDa patients than UCa (Supplementary Fig. 10), it might reflect subtypes of CDa^28,46^. Although a higher CDa sensitivity would be desirable, a high sensitivity in UCa prediction is more clinically important than in CDa, because the surgical option of ileal pouch-anal anastomosis exists for UC only. Thus, CD in general is considered a contraindication for ileal pouch-anal anastomosis, due to high risk of pouch dysfunction, fistula formation, and peripouch sepsis, ultimately leading to a higher rate of pouch excision and the potential for short bowel syndrome^47^.

As a summary, the data sets presented here comprise comprehensive maps of TSSs and enhancers in clinical IBD samples, and show the feasibility of predicting active UC/CD and control status using such data.

## Methods

### Human samples and cohorts

Two independent cohorts, including IBD patients and controls, were analyzed. Cohort 1 (*N* = 94) was used for CAGE assay and subsequent microfluidic real-time quantitative reverse transcription polymerase chain reaction (qPCR) validation. This cohort included 25 patients with active UC (UCa), 17 patients with UC in remission (UCi), 20 patients with active CD (CDa), 3 patients with CD in remission (CDi) and 29 healthy controls (Ctrl). Cohort 2 (*N* = 101) was used for independent qPCR validation of selected target genes. The cohort included 37 UCa, 18 CDa, and 46 Ctrl subjects. All included subjects underwent a routine sigmoidoscopy or colonoscopy as part of their clinical evaluation at the Department of Gastroenterology, Medical Section, Herlev Hospital, Denmark or Department of Gastroenterology, Medical Section, Hvidovre Hospital, Denmark due to their clinical symptoms. They were included into the study as UC or CD patients undergoing lower endoscopy to assess possible disease activity, extension of disease or surveillance for dysplasia, or as controls (endoscopy was performed due to gastrointestinal symptoms but all investigations returned normal). IBD patients were diagnosed established using well-defined criteria^15^. UC patients were graded by Mayo score: a score ≤2 with endoscopic sub-score of 0 (i.e., no macroscopic inflammation) as quiescent (in remission) UC and >2 as active UC, and CD patients were graded in accordance with the Harvey-Bradshaw score: a score <5 as quiescent (in remission) CD and ≥ 5 as active CD. Exclusion criteria were as follows: age >80 or <18, clinical evidence of infection, use of antibiotics or probiotics within 14 days, severe mental illness and pregnancy. Additional data for each cohort is available in Supplementary Table 1 and Supplementary Data 1.

### Ethics

The IBD sample collection and CAGE study was approved by the Scientific Ethics Committee of the Capital Region of Denmark, filed under the ethical permission numbers 36538/H-C-2009-057, 22484/H-C-2009-057, 43691/H-6-2014-051, and 53619/H-6-2014-051, covering both biopsy collection and the application of data to next-generation sequencing approaches. All individuals provided written informed consent to participate in this study. In this context, participation meant that five extra biopsies were obtained in addition to the number of biopsies required on medical grounds, extending the examination time by 2–3 min. Ctrl subjects were a subset of the above set, but were assigned “Ctrl” when all clinical and paraclinical investigations subsequently indicated no inflammation. Participants were informed both orally and in writing in compliance with the Declaration of Helsinki and the guidelines of the Danish National Scientific Ethics Committee. The gut epithelia organoid and monocyte studies were approved by the local bioethical committee (ethical approval numbers 1302159 and 51899, respectively). All samples were obtained from patients who provided informed consent before surgery (for gut organoids) or blood sampling (monocytes).

### Tissue isolation and RNA extraction

Mucosal pinch biopsies (weight on average 15 mg) were obtained from the descending colon using endoscopic forceps. We choose the descending colon in order to avoid intersegmental variation. The endoscopic diagnosis of active or inactive disease was confirmed by histopathology conducted on parallel biopsies taken within an inch of the 1st biopsy. The biopsies were immediately placed in RNAlater® Stabilization Solution (Ambion™, Life Technologies) and kept at 4 °C for 24 h before long-term storage at −80 °C. The RNA was extracted using PureLink® RNA Mini Kit (Ambion™, Life Technologies) with freshly made lysis buffer containing 1% 2-mercaptoethanol (Sigma). The biopsies were homogenized directly in the lysis buffer using an ULTRA-TURRAX® (IKA Works, Inc). The purification was performed following the manufacturer’s instructions including an on-column DNase treatment with the PureLink® DNase Set (Ambion™, Life Technologies). For 16 samples RNA extraction was performed as previously described in ref. ^48^. Quantity and purity of the RNA was determined for all the samples on a Nanodrop ND-1000 spectrophotometer (Thermo Scientific). The purity was measured as the A260/280 ratio and was between 1.9 and 2.1. RNA quality (integrity) was determined using the 2100 Bioanalyzer Instrument (Agilent Technologies) with the Agilent RNA 6000 Pico Kit (Agilent Technologies) as recommended by manufacturer. In cohort 1, the average RNA integrity number (RIN) was 7.6 and no samples were below 5.3. For cohort 2, the average RIN value was 8.0 and no samples were below 6.2.

### Monocyte isolation and stimulation

Blood was freshly collected from IBD patients with informed written consent. First, freshly drawn blood was diluted at a ratio of 1:1 with PBS. Peripheral blood mononuclear cells (PBMCs) were isolated from diluted blood by Ficoll-Paque density gradient centrifugation according to the manufacturer’s instructions (GE Healthcare, Uppsala, Sweden). Harvested PBMCs were used for monocytes isolation. CD14+ monocytes were isolated from PBMCs by negative immunomagnetic bead separation using Monocytes Isolation Kit II (Miltenyi Biotec, CA, USA). After monocyte isolation, 1 × 10^6^ monocytes were plated in each well of 24-well plates (TPP, Trasadingen, Switzerland) in 1 ml growing medium (RPMI-1640 medium containing 10% human serum, 50 IU/ml penicillin, 50 μg/ml streptomycin, and 0.5 mg/ml gentamycin) at 37 °C in a cell culture incubator. Cells were cultured overnight and stimulated with 10 ng/ml TNFα the next day and harvested at indicated time. Control cells were not treated with TNFα but otherwise treated in the same way. Cells were pelleted, snap frozen on dry ice and stored at −80 °C until use. RNA was purified using Qiagen RNeasy Micro Kit as recommended by manufacturer with lysis buffer containing 1% 2-mercaptoethanol (Sigma) and on column DNase digestion. The average yield was 700 ng, with RIN values between 8 and 9.2. TSS expression analysis of 36 TSS targets (as defined below) was done using the microfluidics Fluidigm platform in parallel with biopsy qPCR, as described below.

### Epithelial organoid growth and stimulation

Biopsies of morphologically normal epithelium from patients undergoing surgery for colorectal cancer were aseptically collected. After washing thoroughly with PBS, biopsies were cut into ca. 4 mm pieces and incubated in freshly prepared chelation buffer (96 mM NaCl, 55 mM d-sorbitol, 44 mM sucrose, 10 mM EDTA, 8 mM KH_2_PO_4_, 5.6 mM Na_2_HPO_4_, 1.6 mM KCl, 0.5 mM DTT) for 45 min with agitation on ice. After replacing chelation buffer with PBS, biopsies were manually shaken 3 × 10 s in order to detach colonic crypts. Filtered crypts were suspended in ice cold Matrigel, plated in 48-well plates and maintained in organoid culture medium: Advanced DMEM/F12 (Life Technologies), 1× penicillin/streptomycin (Life Technologies), 10 mM HEPES (Life Technologies), 2 mM GlutaMAX (Life Technologies), 10 mM Nicotamide (Sigma), 1× N2 (Life Technologies), 1× B27 (Life Technologies), 1  mM *N*-acetylcysteine (Sigma), 10 μM Y-27632, 0.5 μM A-83-01 (Tocris), 10 μM SB202190 (Sigma), 100 ng/ml mWnt3a (Cell Guidance Systems), 500 ng/ml mRspondin-1 (R&D systems), 100 ng/ml mNoggin (R&D systems), 50 ng/ml hEGF (Peprotech), 2.5 μM PGE-2 (Sigma). For TNFα stimulation experiments, primary organoid lines from different patients (passage number < 3) were run in technical triplicates and maintained in organoid culture medium supplemented with 10 nM hTNFα (R&D systems) for 0, 4, or 24 h in duplicate wells. Duplicate wells were pooled together and RNA was isolated using Trizol (Invitrogen) in combination with Purelink minicolumns (Life Technologies). The Purelink minikit was used as recommended by manufacturer including the on column DNase step. The average yield was 5540 ng with a RIN between 6.8 and 9.8. TSS expression analysis of 36 TSS targets (as defined below) was done using the microfluidics Fluidigm platform in parallel with biopsy qPCR, as described below.

### CAGE library preparation, sequencing, mapping, and processing

CAGE libraries were prepared according to ref. ^11^ with an input of 1500 ng of total RNA as starting material. Individually prepared CAGE libraries with unique barcodes were pooled (4 per lane). The following four barcodes were used: no. 2 (CTT), no. 3 (GAT), no. 6 (ACG), and no. 8 (ATC). All used primers and adaptors were purchased from Integrated DNA technologies (IDT). A HiSeq2000 instrument from Illumina at the National High-throughput DNA sequencing Centre, University of Copenhagen was used; 30% Phi-X spike-ins were added to each sequencing lane. CAGE reads were matched to their originating samples if barcodes matched identicaly. Linker sequences were removed and reads were filtered, requiring at least 30 in 50% of the bases usingthe FASTX-Toolkit (http://hannonlab.cshl.edu/fastx_toolkit/) with the length of 25 bp. CAGE tags were mapped using Bowtie^49^ (version 0.12.7) to the hg19 assembly using *v* = 2 and otherwise standard settings but allowing for multiple alignments. Subsequently, only uniquely mapping reads were retained. Reads that mapped to chrM were discarded. Supplementary Data 2 shows mapping statistics. In short, for each library on average 17.3 million reads mapped, of these, on average 74% (S.D.± 2.8) mapped to a unique location.

To generate a genome wide map of transcription start sites, 5′ ends of the CAGE tags (CTSSs) that mapped close to each other on the same strand were grouped into tag clusters (TCs) which were used for all the post analysis as in ref. ^50^. In brief: for initial cluster definition, for each library, CTSSs supported only by one CAGE tag 5′ end were ignored. After Tags Per Million (TPM) normalization (CAGE tags per total mapped tags in library*1e6), remaining CTSSs from all libraries were summed into a joint CTSS profile. CTSSs within 20 bp of each other on the same strand were merged into TCs. We trimmed weakly TC expressed tails around TCs using a queue-based trimming algorithm: base pairs were removed iteratively from the edge of TCs, always choosing the most lowly expressed edge, until 10% of the total TPM of the TC had been removed. Trimmed TCs were then quantified in all samples by counting the total number of CAGE tags in each TC and each sample (for this step, singleton CTSSs were included). Unless otherwise mentioned, only TCs having ≥1 TPM in at least three libraries were retained for further analysis. For simplicity, we refer to TCs as “CAGE-defined TSSs”. A summit, or ‘TC peak’, was identified in each TC, defined as the single base-pair position within the TC with highest total TPM coverage across all samples.

### Annotation of CAGE-defined TSSs and gene-level expression

CAGE-defined TSSs were annotated using GENCODE v19 annotation^17^. Canonical TSSs were defined as the most upstream annotated TSS in the GENCODE gene model. CAGE-defined TSSs within ±100 bp of the most upstream annotated TSS for a GENCODE-annotated gene were thus labeled as canonical TSSs. CAGE-defined TSSs ±100 bp from all other GENCODE-annotated TSS were defined as “known alternative TSS”. CAGE-defined TSSs within gene bodies more than 100 bp from an GENCODE-annotated TSS were annotated as “novel alternative TSSs”, CAGE-defined TSSs > 100 bp distant from gene annotation were annotated as novel intergenic TSSs and finally all TSSs located on the antisense strand to annotated genes were defined as novel antisense TSSs. Some analyses were performed at the level of annotated genes, rather than on CAGE-defined TSS level. For these analyses, based GENCODE v19 annotation^17^, we summed up all CAGE-defined TSSs overlapping the gene on the same strand, including intronic TSSs. In case of a TSS overlapping different genes (not different transcripts of the same gene), we used an annotation ranking so that the TSS was associated with a single gene, based on the type of annotation overlap with the following priority order: canonical TSSs > known alternative TSS > novel alternative TSSs.

### Enhancer identification

Enhancer regions were identified from CAGE-derived bidirectionally transcribed loci, as in ref. ^13^ with the following modifications: only enhancer regions candidates whose bidirectionality score |*D*| < 0.6 (*D* ranges from −1 to 1 where *D* = 0 corresponds to a perfectly balanced transcription between strands around the enhancer midpoint, as defined in ref. ^13^) were used. The permissive set was defined by requiring at least two CAGE tags in at least one sample within the enhancer region (as in ref. ^13^), while the strict set was defined by regions having at least two tags in at least eight samples (Supplementary Fig. 4a). Ten technical replicate libraries were included at this stage, but discarded in final expression measurement table. Overlap with FANTOM5 enhancers (http://fantom.gsc.riken.jp/5/datafiles/latest/extra/Enhancers/) was calculated using the *intersectbed* command from BEDTools package^51^.

### Evolutionary conservation analysis of enhancer regions

Enhancer regions from the strict set were aligned by their midpoint. Enhancers whose ±2000 bp flanks from their midpoint overlapped were discarded, because otherwise the same regions would contribute to the analysis more than once. For each nucleotide in the ±2000 bp region, we calculated the average PhyloP evolutionary conservation score based on 100 vertebrate genomes^52^ (http://genome.ucsc.edu/cgi-bin/hgTrackUi?db=hg19&g=cons100way). Enhancers from the permissive set were analyzed in the same way. For comparison, we defined a set of DHS peaks from gut tissue from ENCODE data (https://www.encodeproject.org/). These included transverse colon (acc. numbers: ENCSR504WYA, ENCSR979ZJS, ENCSR790FIS) and sigmoid colon (acc. numbers: ENCSR592DQC, ENCSR907VOR). We excluded the DHS peaks overlapping any gene TCs or enhancers defined in either this study or FANTOM5^13^. We further discarded DHS regions overlapping ±200 bp around any annotated exons or ±500 bp around any annotated TSSs (the same criterion used to define CAGE enhancers). The middle points of remaining DHS peaks were extended 2000 bp and used in the analysis in the same way as the strict enhancer set. Similarly, to assess background levels of conservation, we sampled 500,000 regions of 4001 bp from intergenic regions in canonical chromosomes (chr1-22, X and Y) excluding assembly gaps. Expected background per bp was calculated as mean per position across all random regions.

### Visualization of ChIP signals at predicted enhancer regions

ChIP heatmaps for transcription factor peaks were derived from ChIP-Seq peaks in the ENCODE consortium (version 3, including 161 TFs from 91 cell types). Number of peaks were aggregated for coverage per bp and used as TFBS signals in the heatmap. H3K27ac and H3K4me1 ChIP-Seq signals used for equivalently arranged heat maps were downloaded from the Roadmap Epigenetics Consortium^27^. Heat maps were ordered by the increasing width of enhancers from the strict set and centered at enhancer middle points. ChIP-Seq signals falling in ±2 kb around enhancer middle points were visualized in 401 bins as averages. Binned values were then broken into permilles and assigned to a color gradient ranging from red (high ChIP-Seq signals) to blue (low ChIP-Seq signals).

### TSS-enhancer linkage

TSS-enhancer linkage prediction was assessed by CAGE expression correlation across subjects. Prior to correlation calculation, the TSS and enhancer expression matrices, for all samples including CDi and UCi, were TPM-normalized using edgeR’s RLE-method and log_2_ transformed with a pseudocount of 0.25 (using *calcNormFactors(method* = *”RLE”)* and *cpm(log* = *TRUE))*. The R-function *cor*.*test* was used to test for significant Pearson correlations between pairs of TSSs and enhancers where the TSS peak and enhancer midpoint were within ±500 kb of each other. Resulting *P*-values were FDR corrected using the Benjamini-Hochberg method. TSS-enhancer correlations with *FDR* < 0.05 and a positive correlation were considered significant for the following analyses.

### Definition of enhancer clusters

Consecutive enhancers from the strict set were chained together if their distance was no longer than 15 kb, producing enhancer clusters with varying number of enhancer members. An enhancer cluster was defined as linked with a TSS if at least one of its enhancer members had a significant and positive correlation with the TSS, as defined above.

### Exploratory differential expression data analysis

We defined an expression matrix consisting of CAGE-defined TSSs (TCs as defined above) and their summed number of CAGE tags for each subject. Raw counts were normalized to log-TPM values and subjected to a variance stabilizing transformation using the varianceStabilizingTransformation function from the DESeq2 package^53^ (with blind = TRUE) to improve linear modeling of the data by dampening the mean-variance trend inherent in count-type data. Principal Component Analysis (PCA) was used to explore major expression patterns in the dataset using the *prcomp* function from base R (with *scale* *=* *TRUE* and *center* *=* *TRUE)*. This revealed four major groups which were not related to any specific part of the study design, but was correlated with the date, on which the individual samples were prepared (Supplementary Fig. 11). This effect may be due to change of reagent batches e.g. the biotin hydrazine and antarctic phosphatase which were replaced between batches. To remove these batch effects for plotting purposes, we used the *ComBat* function (default parameters) from the *sva* R-package^54^, after first performing a variance stabilizing transformation with blind = FALSE (Supplementary Fig. 11). All samples and all five experimental groups (CDa, UCa, CDi, UCi, and Ctrl) were used for the batch effect correction for the initial PCA plot (Figs. 2a and 3d). Only CDa, UCa, and Ctrl were used for batch-corrected gene-level expression plots for the later parts of the main text (Fig. 3f, Supplementary Fig. 2d, e, h and Supplementary Fig. 6). For differential expression analysis, batches were taken into account as blocking factors (see below). As a complement to the unsupervised PCA plot, we also performed a supervised analysis using Partial Least Squares Discriminant Analysis (PLS-DA), via the Discriminer R-package and the *plsDA* function. The same variance stabilized expression values used for ComBat (with blind = FALSE) was used as input to the PLS-DA with components set to two (comps = 2). 95% confidence ellipses were added using stat_ellipse from ggplot2 (with type = ”t”).

### Statistical tests for differential expression

The *edgeR* package was used to test for differential expression at both TSS- and gene-level using the GLM quasi-likelihood framework (default settings were used, with the exception that robust estimation at the empirical Bayes stage was used). As described in the main text, only CDa, UCa, and Ctrl samples were included in this analysis. The four major batches were included as blocking factors (Supplementary Data 1). The following contrasts were used: (i) IBD: average of CDa and UCa different from Ctrl and (ii) CDvsUC: CD different from UC. Resulting *P*-values were corrected for multiple testing Benjamini–Hochberg method (producing *FDR* values). To obtain the final sets of differentially expressed TSSs (or genes) used in the main text, we filtered TSSs (or genes) with |log_2_ fold change| > 1 and FDR < 0.05. The IBD_up_/IBD_down_ sets correspond to TSSs (or genes) with a positive/negative log_2_ fold change in contrast i) and the CD_spec_/UC_spec_ sets correspond to TSSs (or genes) with a positive/negative log_2_ fold change in contrast ii), where a positive log_2_ fold change corresponds to higher expression in CDa compared to UCa (and vice versa). The diffSpliceDGE function was used to identify differential TSS usage within annotated genes. The Simes method was used to aggregate TSS *P*-values to gene level. To decide whether including additional covariates would improve modeling of the data, we added effects of various medical labels, such as medication, smoking, gender, etc. to the design matrix described above, and used limma-voom to test for differential expression in a similar manner (due to the higher speed of limma compared to edgeR). Most extra covariates did not have any significantly differentially expressed genes, except minor effects for gender and SASA treatment. Since these were far smaller than the effects of either inflammation or batch, we chose not to include them in the main analysis shown in the main text (Supplementary Fig. 2a).

### Differential expression analysis of enhancer regions

Because edgeR analysis has limited power for globally lowly expressed entities like enhancer RNAs, we used an alternative approach to assess differential expression between conditions for these data. The enhancer RNA data were normalized using the median-based method implemented in the DESeq package (default)^55^. Instead of normalizing directly on the counts of enhancer RNA, we used the counts from TSSs from the same samples. These signals are typically orders of magnitude higher and the measured median counts are therefore associated with much smaller uncertainties. The R-package EBSeq (version 1.10.0) was used for the identification of differentially expressed enhancers based on the normalized data^30^. EBSeq assumes that any given data row (enhancer count data for CDa, UCa, and Ctrl samples, respectively) can follow one of a number of possible expression patterns, corresponding to different plausible hypotheses about that specific enhancer. For the three conditions considered here, there are a total of five possible patterns

Pattern 1: Enhancers expressed at the same level in CDa, UCa, and Ctrl (non-differential expression).

Pattern 2: Enhancers with the same expression level in CDa and UCa, and a different expression level in Ctrl (general IBD enhancers).

Pattern 3: Enhancers having similar expression levels in CDa and Ctrl, while being expressed differently in UCa (enhancers specific for UCa).

Pattern 4: Enhancers having similar expression levels in UCa and Ctrl, while being expressed differently in CDa (enhancers specific for CDa).

Pattern 5: Enhancers expressed differently in all three conditions.

For each enhancer in the dataset, EBSeq computes the posterior probabilities that it follows each of these five patterns. EBSeq also produces posterior estimates of the fold change in expression levels between each pair of the three conditions for any given enhancer. Another feature of the EBSeq method is that it uses hierarchical (or multi-level) modeling: the expression level (read count) of individual enhancers is assumed to follow negative binomial distributions whose *r*- and *q*-parameters are inferred from the data. For any given enhancer, these parameters may be identical across conditions (non-differential expression) or different across conditions (differential expression). Different enhancers each have their own set of parameters, but all *q*-parameters are assumed to be related, in that they are drawn from a higher-level beta distribution whose (hyper) parameters are also estimated from data. This multi-level modeling approach results in “borrowing of strength” (inference from data on one enhancer will help inform inference for other enhancers), and “shrinkage” (parameter estimates for enhancers with little data will be automatically shrunk toward the overall mean). Since the majority of all enhancers are not differentially expressed the approach is conservative (it requires a substantial amount of data to pull log-fold estimates away from zero) and thereby helps avoiding issues with multiple testing.

Specifically, the analysis was performed using the *EBMultiTest* function in the EBSeq package with *maxround* set to 100 iterations. Convergence was checked by inspecting plots of the hyper-parameters *Alpha* and *Beta* and the mixture parameter *P* and ensuring their values had settled at stable values at the end of the iterations. The function QQP was used to generate QQ-plots confirming that the beta prior used in the analysis was appropriate (data not shown). We used a posterior probability cutoff of 0.9 for deciding when to label an enhancer as differentially expressed, corresponding to a false discovery rate of 0.1. Labeled enhancers were then reconciled with the classification of differential expression made for TSSs as follows (*P* = posterior probability):

IBD_up_: *P*(Pattern 2) > 0.9 and (CDa and UCa positive log fold change vs. Ctrl)

IBD_down_: *P*(Pattern 2) > 0.9 and (CDa and UCa negative log fold change vs. Ctrl)

CDa_spec_: *P*(Pattern 4) > 0.9

UCa_spec_: *P*(Pattern 3) > 0.9

There is no simple way of accounting for batch effects in the software used here, and we therefore performed the analysis independently for the two batches. Results reported in this paper are for the larger batch (58 subjects), but we found very similar results for the smaller batch (Supplementary Fig. 4d). All analyses were performed both on the (larger) permissive enhancer set, and the (smaller) restricted enhancer set. On the whole, enhancers identified as being differentially expressed in the strict data set were a subset of those identified based on analysis of the permissive data set (data not shown).

### Gene ontology and protein interaction analysis

For differential expression analysis performed at the gene-level, we performed enrichment tests for GO-terms using the gProfiler online tool^56^ via the associated gProfiler R-package (https://CRAN.R-project.org/package=gProfileR) using the *gProfiler* function. Default settings were used, except the background set (i.e., “universe”) was set to all expressed genes. The results of the enrichment analysis on each of the four sets can be found in Supplementary Data 7. Additionally, we also queried each of our gene-level differential expression sets against the STRING database of protein-protein interaction networks (using default settings, but removing proteins with no connections to any other proteins).

### FANTOM5 cell specificity enrichment analysis

For TSSs that were differentially expressed, we performed enrichment tests for FANTOM5 cell specificity of TSSs. We downloaded the table of cell type specificity of CAGE tag clusters from http://fantom.gsc.riken.jp/5/datafiles/phase1.1/extra/Sample_ontology_enrichment_of_CAGE_peaks/. For each TSS, we noted whether it was differentially expressed and if it overlapped (based on genomic coordinates) with a FANTOM5 cell-specific tag cluster. We used the *table* function from base R to create a 2 × 2 contingency table for differential expression statistics and cell specificity, and tested for independence between rows and columns of this table using a Fisher’s Exact test (via R’s *fisher*.*test*). *P*-values for each cell category were corrected for multiple testing using the Benjamini–Hochberg method. The complete table of statistics for all cell type terms can be found in Supplementary Data 8.

### Transcription factor binding site enrichment analysis

Promoter regions were defined as follows: for each TSS defined as differentially expressed in the respective sets (IBD_up_, IBD_down_, as defined above), we extracted the genomic positions corresponding to −500 and +100 bp around each such TSS peak. Background promoters were defined as corresponding regions from TSSs, which were not part of any of the four differential expression sets (IBD_up_, IBD_down_, UC_spec_, CD_spec_). For enhancer regions, we selected enhancer regions that were differentially expressed in the respective sets (IBD_up_, IBD_down_, as defined above). We extracted the genomic positions corresponding to ±300 bp around the midpoint of each such enhancer region. Background enhancers were defined as corresponding regions from enhancers not part of any differentially expressed set. Next, we used the HOMER tool^31^ to score enrichment of sites corresponding to known motifs in the regions and background described in the previous paragraph (using default settings, except *genome* = *hg19* and *size* = *given*, and *mset* = *auto* which defaults to “vertebrate”). Sequence logos for these motifs were acquired from http://homer.salk.edu/homer/motif/HomerMotifDB/. HOMER motifs were manually paired with CAGE-defined transcription factor expression (ComBat-corrected gene-level expression).

### ENCODE transcription factor ChIP peaks enrichment

ChIP-Seq peaks were downloaded from the ENCODE consortium (161 TFs from 91 cell types; see above section for heatmap visualization for details). We analyzed the enrichment of each set of TF peaks in a given set of enhancer regions (e.g., IBD_up_ enhancers) by constructing a contingency table as defined in Supplementary Table 2.

Enrichment of a given TF in the region of interest was defined as ${\mathrm {log}}_2\left( {\frac{{{\it{A}}/({\it{A}} + {\it{C}})}}{{{\it{B}}/({\it{B}} + {\it{D}})}}} \right)$ (terms defined in Supplementary Table 2) and significance of enrichment was tested by Fisher’s exact test for each TF based on the above contingency table. For the analysis in Supplementary Fig. 5 the regions of interest were IBD_up_ or IBD_down_ enhancers and the background regions were non-differentially expressed enhancers. In Fig. 5d, we only focused on enhancers that were associated with IBD_up_ TSSs by expression correlation as defined previously. Among these enhancers, we defined the regions of interest as the enhancers from large enhancer clusters (>6 enhancers in a cluster) and the background regions as singleton enhancers (not part of any cluster).

### Selection of biomarkers

In order to create an initial candidate list for machine learning-based selection and classification, we reasoned that any single prioritization method has its own disadvantages. Therefore, we performed an ensemble approach where we integrated the results of multiple analysis methods each aimed to extract TSSs with high power to distinguish the subject groups. As our main goal was classification by machine learning, we will refer to these TSS regions as “features” in this section (instead of biomarkers as in the main text). Because the shared inflammatory response in UCa and CDa vs. Ctrl was strong, we divided our analysis into an IBD set of features (shared CDa and UCa up- or downregulation vs. Ctrl), and a set of features corresponding to features differentially expressed between UCa and CDa.

The ensemble analysis consisted of the following components:

i. edgeR^18^. In a similar fashion to the quasi-likelihood implementation explained above, standard edgeR (*fitGLM* and *glmLRT*) was used to test for effects of CDa vs. Ctrl, UCa vs. Ctrl, CDa vs. UCa, and CDa&UCa vs. Ctrl (shared inflammatory response), while controlling for batch effects. Extracted TSSs had a Benjamini–Hochberg FDR < 0.05 and a |log2 fold change| >1, for a total of four sets.
ii. Partial Least Squares Discriminant Analysis (PLSDA): The *plsDA* function from the R-package DiscriMiner (https://cran.r-project.org/package=DiscriMiner) was used to perform PLSDA with two components on the variance stabilized expression values from above, using all samples, including quiescent patients, in three groups: CDa+ CDi, UCa+ UCi, and Ctrl. The 500 TSSs with the highest and lowest loadings for the component separating Ctrl from the remaining samples were extracted for a total of 1000 TSSs.
iii. ComBat^54^ followed by PCA: ComBat corrected values were used for PCA analysis. We identified the component that contributed the most to the difference between the two states and sampled 1000 TSSs from each side with the highest rotation values. We focused on the PCAs that gave the most explanations for each of the comparisons (PCA1 for shared IBD-specific signal and PCA2 for CD_spec_ and UC_spec_), producing two lists for IBD (from each side one list) and one list for CD_spec_ and UC_spec_ (from each side one list).
iv. ComBat followed by limma^57^: Combat normalized data (as described above) was used for differential expression analysis using the limma package and the *lmFit* function. TSSs that passed the threshold of log fold change > |1| and FDR < 0.05 were taken into account making two lists for IBD (overrepresented in UCa vs. Ctrl and overrepresented in CDa vs. Ctrl) and one list for CDa vs. UCa.
v. Random Forest analysis. We utilized the inherent ability for Random Forests (RFs) to rank features by their importance for classification accuracy. To do this we used the *randomForest* function from the randomForest R package (https://CRAN.R-project.org/package=randomForest) on Combat-normalized TC expression data (as described above in the “Exploratory data analysis” section). To circumvent the “Large *p*, small *N*” problem with our data (74 samples, 48,593 TCs), which makes difficult to find the most predictive features, we used an iterative feature selection approach where we in each step remove a subset of the least important features. More specifically: in each iteration we first evenly divided all the data into subsets each containing max 500 features. For each of these data subsets a RF was trained to classify the patient labels based only on the data in that particular subset. Next, for each of the data subsets, we removed the 5% of features that had the lowest classification power (lowest MeanDecreaseAccuracy values) resulting in a list of features that is analyzed in the next iteration of the procedure. To make sure an important feature was not lost due to a challenging subset of data, in each iteration we randomly divided all our data into subsets 10 times. In other words, features had several chances of showing its importance. This iterative procedure was terminated when less than 200 features were left. Each RF was made with default parameters except specifying that 501 trees per forest should be generated and specifying the number of variables randomly sampled as candidates at each split by setting the “mtry” parameter to (sqrt(*x*) −1) * 2, where *x* was the number of features supplied to the individual RF. Using this approach we generated four candidate lists: two that enabled classification of IBD patients (CDa and UCa) from healthy patients (Ctrl) and two lists that aimed at differentiating between CDa and UCa. For the two lists in each category, one was based on the full data set (*N* = 48,593 TCs) and the other was based on a high confidence dataset (*N* = 9699 TCs). The high-confidence data set was obtained by requiring that (i) the tag cluster was annotated as “5_prime_UTR”, “intergenic”, “known_alternative_cds”, “known_alternative_tss”, “proximal” or “tss”. (ii) the width of the TC was in the range of 2–100 nucleotides (both included). (iii) the TC was expressed more than 10 TPM in at least 3 subjects.

As a summary, the ensemble produced 10 candidate sets for separating CDa and UCa from Ctrl (3 edgeR, 4 ComBat, 2 RandomForest, 1 PLSDA) and 5 candidate sets for separating CDa and UCa (edgeR, limma, 2 RandomForests, and PCA).

Next, we overlapped the lists from each comparison to find a consensus set of classification candidates. For the IBD vs. Ctrl comparison, we selected features that appeared in at least 9 out of 10 lists, resulting in 63 features. From the UC/CD set we selected the 169 features that appeared in at least 2 out of 5 lists. The reasoning behind this unbalanced selection was to have a higher number of features for the more difficult task of distinguishing CDa from UCa, compared to the easier task of distinguishing Ctrl from UCa or CDa.

The two lists were merged and a short list of expert-curated known and novel biomarkers (21 TSSs and 10 enhancers) was added. The combined list was then further pruned by manual curation, taking into account overall expression strength and genomic loci complexity. In total, the initial list comprised 263 features, which was used for initial classification using CAGE data (see below).

### Microfluidic qPCR analysis

For qPCR analysis, the 263 features identified above were assessed for qPCR primer targeting suitability. 181 candidates were taken into the primer design stage, where genomic complexity and primer design feasibility was evaluated manually.

Primers were designed using Primer3 (v.0.4.0)^58^ aiming for an amplicon length of 70–110 bp and a primer melting temperature of 60 °C calculated by Breslauer thermodynamics. Primers were designed to be intron-spanning when possible, and each designed primer-set was analyzed with the UCSC browser in-silico PCR tool in order to ensure a unique region would be amplified. Primers were synthesized by Integrated DNA Technologies (IDT). Primer sequences are shown in Supplementary Data 16. Primer amplification efficiencies and dynamic ranges were acquired from standard curves constructed from several separate dilution series of pooled cDNA: with the dilutions 1:5, 1:25, 1:100, 1:500, and 1:2500. Melting curves of amplicons were measured to ensure primer specificity. 161 primer pairs (features) successfully passed our quality control and were used for analysis of cohort 1 on the Fluidigm platform.

The cDNA synthesis and preamplification for cohort 1 was performed as described previously^59^. Total RNA (500 ng RNA, from biopsies) was converted into cDNA using QuantiTECT Reverse Transcription kit (Qiagen), using a mix of random and oligo-dT primers, as per the manufacturer’s instructions. We performed two separate cDNA reactions for each RNA sample. Preamplification was performed using TaqMan PreAmp Master Mix (Applied Biosystems). A 500 μl primer mix (200 nM) combining all primers to be used on the Fluidigm plate was prepared by pooling 5 μl of all primer pairs (20 μM) and filling up the remaining volume with low EDTA TE-buffer (VWR International). TaqMan PreAmp Master Mix (5 μl) was mixed with 2.5 μl of the 200 nM primer mix, 2.5 μl diluted cDNA, and incubated at 95 °C for 10 min, followed by 15–21 cycles of 95 °C for 15 s and 60 °C for 4 min (the number of cycles depended on the expression measured by CAGE). 16 U of Exonuclease I (New England BioLabs) was added to the preamplified cDNA, and incubated 30 min at 37 °C, 80 °C for 15 min. The preamplified cDNA was diluted with low EDTA TE-buffer (VWR International) either 1:5 if the cDNA was preamplified for 21 cycles or 1:10 if the cDNA was preamplified for 19 or 15 cycles.

RNA expression was analyzed by real-time qRT-PCR in the microfluidics system BioMark^™^ 96.96 Dynamic Array (Fluidigm) following the protocol described previously^59^. The following cycle parameter was used: Thermal Mix with 2 min at 50 °C, 30 min at 70 °C, 10 min at 25 °C, UNG and Hot start with 2 min at 50 °C, 10 min at 95 °C, followed by 35 cycles with denaturing for 15 s at 95 °C and annealing/elongation for 1 min at 60 °C. Melting curves were generated after each run to confirm a single PCR product (from 60 °C to 95 °C, increasing 1 °C per 3 s). We performed reactions in duplicates. Non-template controls were used to indicate problems with sample contaminations or non-specific amplification. To assess potential DNA contamination, non-reverse transcriptase controls were used.

We used the Fluidigm Real-Time PCR Analysis software 3.0.2 (Fluidigm) to aquire Cq values. These were exported to GenEx (MultiD) for data pre-processing including normalization to reference genes, interplate calibration, individual PCR efficiency correction for each primer assay, and averaging of cDNA technical replicates. Using GeNorm^60^ and NormFinder^61^, we identified the most stable reference genes of the 10 reference genes included on the plates. This was done separately for all plates run with the same number of preamplification cycles. The used reference genes for each preamplification batch are specified in Supplementary Data 16. By normalizing to these reference genes, ∆Cq values were calculated and used in the down-stream analysis.

### Feature selection for cohort 2

The 161 features were reduced to 36 features by ranking the features for classification power. Briefly, a RF was trained to predict subject groups, both using all groups and one-vs.-rest specification (i.e. UCa vs. non-UCa) using the randomForest R package^62^. The optimal value for the *mtry* parameter was found using 100 five-fold cross-validations using the caret package (https://cran.r-project.org/web/packages/caret/index.html) *train*-function and *ntree* = 1000. Feature importance for both classification tasks were extracted using the *importance* function with *type* = 2 (mean decrease in node impurity or gini index). Primer pairs with a high importance in both complete and one-vs.-rest classification were selected as the most predictive. In addition to these RF predictive features, six gene-based features were selected to validate pathology-based findings in the first cohort.

cDNA synthesis and preamplification for cohort 2 was performed as described above for cohort 1 with an adjustment of the number of pre-amplification cycles. In this cohort cDNA was either preamplified 15 or 20 cycles, depending on expression level of the primer targets. The preamplified cDNA was diluted with low EDTA TE-buffer (VWR International) either 1:5 if the cDNA was preamplified for 20 cycles, or 1:10 if the cDNA was preamplified for 15 cycles. RNA expression was analyzed by real-time qRT-PCR in the microfluidics system BioMark^™^ 192.24 Dynamic Array (Fluidigm) following the same protocol as used for the cohort 1 runs, with volumes adjusted to the 192.24 format, as described by manufacturer. Expression data (∆Cq values) were acquired and treated similar to cohort 1 (see above).

### qPCR analysis of monocyte and organoid samples

cDNA synthesis and pre-amplification was performed as described for cohort 1 and 2, but with an input of 300 μg (organoids) or 440 μg (monocytes) of RNA in the cDNA synthesis due to limited amounts of RNA. RNA expression was analyzed by real-time qRT-PCR in the microfluidics system BioMark^™^ 192.24 Dynamic Array (Fluidigm) as described for cohort 2. Monocyte samples and organoid samples were placed on two independent plates.

### Batch correction and expression normalization of qPCR data

For each primer all ∆Cq values from cohort 1 and cohort 2 were batch corrected using the *removeBatchEffect* function from limma, giving the subject/group labels (CDa, UCa, Ctrl) as covariates and cohort and hospital of origin as batch effects and specifying the ‘robust’ method should be used for the linear models. To make organoid and monocyte data comparable with the batch corrected data a batch correction factor, obtained as the mean of the batch correction factors for the two hospitals, was applied to the organoid and monocyte data. Afterwards the batch corrected ∆∆Cq values were calculated in a two-step process: (1) *x* = 2^(−1* ∆Cq)^. (2), ∆∆Cq = log2(*x*/min(*x*)).

### Preprocessing of microfluidic qPCR data

For classification analysis of cohorts 1 and 2 (see below), only Ctrl, UCa, and CDa subjects were considered. We removed one subject and one primer pair from the analysis which both had >10% “NA” values from the ∆∆Cq Fluidigm data (Supplementary Data 18), making the final feature count 35. The remaining missing values (*N* = 16, 0.26% of total) were imputed via bagged regression trees using the *preprocess* function from the “caret” R package (https://CRAN.R-project.org/package=caret) specifying methods to the *bagImpute* function. Experimental practice and reporting were performed according to MIQE guidelines.

### Comparison of organoid, biopsy, and monocyte qPCR data

Only measurable ∆∆Cq Fluidigm data were analyzed (values labeled “missing” or “infinite” were discarded). For each qPCR primer we defined the following quantifications:

i. Inflammatory response in UCa or CDa as log_2_(∆∆Cq__CDa/UCa_ (average(∆∆Cq__all_Ctrl_))^−1^).
ii. 4 or 24 h TNFα response in gut epithelia organoid as log_2_(∆∆Cq__organoid_TNF_4h/24h_ (average(∆∆Cq__all___organoid_TNF_0h_))^−1^)
iii. 4 or 24 h TNFα response in monocyte as log_2_(∆∆Cq__monocyte_TNF_4h/24h_ (average(∆∆Cq__all___organoid_TNFa_0h_))^−1^)

Median of responses across replicates or subject group (CDa, UCa, monocyte TNF 4 h, monocyte TNF 24 h, organoid TNF 4 h and organoid TNF 24 h) was then used to indicate the IBD or TNFα response of the group. Specifically, if the median IBD or TNFα response of a qPCR primer in a sample group was >0, it was labeled upregulated in the sample group, and vice versa. qPCR primers with missing median in any of the sample groups were discarded, resulting in analysis of 30 qPCR primers in Fig. 2f and Supplementary Fig. 3b, c.

### Real-time quantitative PCR

For specific enhancer RNA expression validations (Fig. 4), we used the Applied Biosystems® QuantStudioTM 6 Flex Real-Time PCR System, with the 384-well plate. This platform allows for higher reaction volumes compared to the Fluidigm system. The cDNA preparations were done as described for cohort 1. All samples were run in triplicates in 5 μl reaction volumes using SYBR Select Master Mix (Life Technologies) as described by manufacturer, with a primer concentration of 0.5 μM, and under the following conditions: 50 °C for 2 min, 95 °C for 2 min, 45 cycles of 95 °C for 15 s, 58 °C for 1 min. All results were normalized to the reference gene PIAS4.

### Prediction of subject diagnosis using random forests

For all three classification analyses in Fig. 7, we used a two-step RF classification approach with the *randomForest* function from the randomForest R package (https://CRAN.R-project.org/package=randomForest). In this approach, the first step was a RF trained to distinguish IBD (CDa and UCa) from Ctrl and the second step was a RF trained to distinguish CDa from UCa. The two-step approach was chosen over an all-in-one approach because, based on five-fold cross-validation in the CAGE data, resulted in better CDa sensitivities (data not shown).

To optimize the parameters most important for a RF model, *m* indicating the number of variables randomly sampled as candidates at each split and *n* indicating the minimum size of terminal nodes (corresponding to the *mtry* and *nodesize* options in the R RandomForest package), we used a grid search approach testing each pairwise combination where $n = \{ 2,3,4,5\}$ and $m = \left( {\sqrt {x - 1} } \right)\ast y$, where *x* is the number of features supplied to the RF and *y*={1/2, 3/4, 1, 6/4, 2}. For each of the three classifications analyses performed, the final *m* and *n* parameters were chosen as the combination giving the highest overall accuracy in the two-step RF approach based on the average result of five-fold cross-validation in cohort 1. Apart from this all RFs were created with default parameters except specifying that each RF should have 1001 trees.

To assess the accuracy of the classification model we performed 101 RF iterations of 5-fold cross validation. The only exception to this was the classification of subject labels on cohort 2, where 101 RFs were created based on data from cohort 1 and used for prediction in cohort 2. For each of the 101 iterations, the overall accuracy and CDa, UCa, and Ctrl-group-specific accuracy, sensitivity and specificity were calculated. For each of these, we reported the average and 95% confidence interval across the 101 iterations. The CDa, UCa and Ctrl-group specific performance measures were calculated by for each label recasting the problem in a binary setting (e.g. for the “Ctrl” label converting both CDa and UCa to “non-Ctrl”) and then assessing accuracy, sensitivity, and specificity. The overall accuracy was assessed on all three categories.

To assess the observed vs. expected performance in classification, we trained 1001 RF models on randomly shuffled labels (again only based on cohort 1 data). These models were then used to predict the patient labels of cohort 2 and these predictions were compared to the true labels. Performance measures were calculated as described above.

To analyze the relation between number of features in the model and classification accuracy, for a selected number of features referred to as *i*, we randomly selected *i* features from the total set of features and performed five-fold cross-validation with the same parameters as identified by the grid search above. This procedure was repeated 11 times for each selected *i*. The average accuracy and 95% confidence interval of these repeated analyses were reported for each *i*.

### Prediction of subject diagnosis using XGboost

We used the XGboost framework^41^ to perform the same classification as done with Random Forests in Fig. 7c (training on cohort1 qPCR data and evaluating on the independent cohort 2 qPCR data). For additional power, each sample was scored using a database of curated pathways (wikipathways.org). Briefly, for each patient, we computed pathway expression scores, based on the sum of overlapping genes in each pathway and the qPCR data. From the 569 original pathways in the database, 16 returned a non-null score. These scores were then used to provide 16 supplemental features, on top of the 35 original ones, for the training of the classifier. After parameter optimization on the training data (*max_depth*, *min_child_weight and subsample)*, the final classifier was used on the cohort 2.

### Analysis of GWAS data

A recent version of the GWAS catalog was downloaded (2016-12-15) and lifted to hg19 using the *gwascat* R-package (http://bioconductor.org/packages/gwascat/). To calculate linkage disequilibrium (LD), we used genotype data from whole-genome sequencing of Europeans. This was obtained from the VCF files from the 1000 Genomes CEU population release 20130502. We use only diallelic SNPs with <5% missing genotypes that were polymorphic in the CEU population^39^. SNPs from the GWAS catalog were merged with 1000G SNPs by genomic position, and PLINK^63^ was used to calculate LD between all SNPs within 500 kb (using the function *plink -r2 -ld-window-kb 500 -ld-window 99999 -ld-window-r2 0*.*5)*. Pairs of SNPs were considered to be in LD if *R*^2^ > 0.75. LD clumps were found by expanding all SNPs with their associated LD-SNPs, and then reducing the set by merging overlapping regions.

To assess LD clump enrichment, Empirical Bayes shrinkage was performed with the ebbr R-package (https://github.com/dgrtwo/ebbr) package using the *ebb_fit_prior* and *augment* functions (with default arguments). Prior means were calculated as *alphaz(alpha*+*beta)*^−*1*^ and added to the plot of shrunken proportions. LD-clump (one-sided) enrichment was calculated using the *kegga* function from the limma R-package^57^, but using user-supplied sets: *universe* was set to all TSSs/enhancers, *de* the respective differentially expressed sets and *gene*.*pathway* set to all promoters overlapping LD clumps for a GWAS disease. No trend based on an additional covariate was used.

Partitioned heritability analysis was performed using the ldsc tool from (https://github.com/bulik/ldsc)^40,64^. The two analyses below were done based on recommended practice by the creators of the tool: https://github.com/bulik/ldsc/wiki/LD-Score-Estimation-Tutorial, https://github.com/bulik/ldsc/wiki/Partitioned-Heritability). LD-scores were calculated for TSSs, enhancers, IBD_up_ and IBD_down_ sets (including both promoter (−500/+100 bp around TSS peaks) and enhancers (±300 bp around enhancer midpoints)). IBD SNP summary statistics were obtained from the International IBD Genomics Consortium, https://www.ibdgenetics.org/downloads.html (downloaded 2016-12-22). SNP summary statistics were processed using *munge_sumstats*.*py* (with settings -N-cas 12882 -N-con 21770 for sample sizes (as described in ref. ^8^). Base model enrichment statistics for this set of GWAS summary statistics can be seen in Supplementary Fig. 8b. All four extra categories were added to the baseline model for Fig. 6d. Additionally, each new annotation was separately added to the baseline model in sequence, similar to the cell-type group analysis from the original paper^40^. Proportions, enrichments and coefficients were obtained from each model and compared (Supplementary Fig. 8c)

### Data availability

CAGE data from this study has been deposited in GEO database under accession number GSE95437. Microfluidics qPCR data are available in Supplementary Data 9, 17, 18.

## Electronic supplementary material

Supplementary Information

Description of Additional Supplementary Files

Supplementary Data 1

Supplementary Data 2

Supplementary Data 3

Supplementary Data 4

Supplementary Data 5

Supplementary Data 6

Supplementary Data 7

Supplementary Data 8

Supplementary Data 9

Supplementary Data 10

Supplementary Data 11

Supplementary Data 12

Supplementary Data 13

Supplementary Data 14

Supplementary Data 15

Supplementary Data 16

Supplementary Data 17

Supplementary Data 18
