# Supplementary material for: Characterization of the enhancer and promoter landscape of inflammatory bowel disease from human colon biopsies
Source: Nat Commun. 2018 Apr 25;9:1661. doi: 10.1038/s41467-018-03766-z (PMC5916929; doi:10.1038/s41467-018-03766-z)
Supplement: Supplementary file 1 — Supplementary Information [file 41467_2018_3766_MOESM1_ESM.pdf]

Supplementary information for:
Characterization of the enhancer and promoter landscape of inflammatory bowel disease from human colon biopsies, Boyd et al

Supplementary Table 1

Description: Levels	Ctrl / Internal	UCa / Internal	UCi / Internal	CDa / Internal	CDi / Internal	Ctrl / External	UCa / External	CDa / External
Age (median)	*54.0*	*39.0*	*48.0*	*31.5*	*26.0*	*56.0*	*42.0*	*43.0*
HB (median)	*NA*	*NA*	*NA*	*7.5*	*2.0*	*NA*	*6.5*	*8.0*
Mayo (median)	*NA*	*5*	*0*	*NA*	*NA*	*NA*	*8*	*NA*
Gender: F/M/NA	*15/14/0*	*13/12/0*	*12/5/0*	*8/12/0*	*3/0/0*	*21/25/0*	*17/20/0*	*8/9/0*
Smoking: FALSE/TRUE/NA	*17/12/0*	*21/4/0*	*12/5/0*	*11/9/0*	*2/1/0*	*38/8/0*	*35/2/0*	*16/1/0*
Familial: FALSE/TRUE/NA	*0/0/29*	*22/3/0*	*14/3/0*	*11/7/2*	*1/2/0*	*5/0/41*	*34/2/1*	*16/1/0*
Severity: healthy/remission/mild/moderate/severe/NA	*29/0/0/0/0/0*	*0/2/11/11/1/0*	*0/17/0/0/0/0*	*0/0/10/8/2/0*	*0/3/0/0/0/0*	*46/0/0/0/0/0*	*0/0/8/20/9/0*	*0/0/11/4/2/0*
Age_at_diagnosis: <18/>18/NA	*0/0/29*	*2/23/0*	*2/15/0*	*1/19/0*	*2/1/0*	*0/0/46*	*4/32/1*	*4/11/2*
Duration_of_disease: <10/>10/NA	*0/0/29*	*17/8/0*	*8/9/0*	*19/1/0*	*2/1/0*	*0/0/46*	*27/9/1*	*7/9/1*
SASA: FALSE/TRUE/NA	*29/0/0*	*6/19/0*	*4/13/0*	*15/5/0*	*3/0/0*	*41/0/5*	*3/25/9*	*11/0/6*
LASA: FALSE/TRUE/NA	*29/0/0*	*20/5/0*	*14/3/0*	*20/0/0*	*3/0/0*	*41/0/5*	*8/20/9*	*11/0/6*
SSB: FALSE/TRUE/NA	*29/0/0*	*23/2/0*	*17/0/0*	*18/2/0*	*3/0/0*	*41/0/5*	*24/4/9*	*11/0/6*
LSB: FALSE/TRUE/NA	*29/0/0*	*21/4/0*	*17/0/0*	*20/0/0*	*3/0/0*	*41/0/5*	*22/6/9*	*10/1/6*
TNF: FALSE/TRUE/NA	*29/0/0*	*24/1/0*	*17/0/0*	*17/3/0*	*2/1/0*	*41/0/5*	*26/2/9*	*9/2/6*
AZA: FALSE/TRUE/NA	*29/0/0*	*20/5/0*	*16/1/0*	*16/4/0*	*2/1/0*	*41/0/5*	*22/6/9*	*8/3/6*
Antibiotics: FALSE/TRUE/NA	*26/3/0*	*23/2/0*	*17/0/0*	*16/4/0*	*3/0/0*	*41/0/5*	*28/0/9*	*11/0/6*
Other: FALSE/TRUE/NA	*29/0/0*	*24/1/0*	*17/0/0*	*18/2/0*	*3/0/0*	*41/0/5*	*28/0/9*	*10/1/6*
Surgery: FALSE/TRUE/NA	*0/0/29*	*25/0/0*	*17/0/0*	*17/3/0*	*3/0/0*	*0/0/46*	*28/0/9*	*8/3/6*

Clinical details of subjects in cohort 1 and 2, divided into the five diagnosis groups used in the study. A subset of these were used as additional covariates in Supplementary Figure 2
A. The first column describes the clinical characteristics, along with the statistic or levels used for summarizing within groups. In all rows, NA indicates missing data. All medians were calculated after discarded NAs.

Age: Median age when biopsied.
HB_score: Median Harvey-Bradshaw activity index for Crohn's disease.
Mayo_score: Median disease activity index for ulcerative colitis.
Gender: Female (F) or Male (M).
Smoking: Whether the subject smokes
Familial: Family history of IBD
Severity: Healthy/remission/mild/moderate/severe/NA: Histological evaluation of the degree of inflammation. Remission corresponds to inactive patients.
Age at diagnosis: Whether the patient was diagnosed before or after 18 years of age.
Duration of disease: Whether the patient had the disease for more than 10 years at the time of biopsy sampling.
SASA: Treatment with systemic mesalazin (5-ASA).
LASA: Treatment with local mesalazin (5-ASA)
SSB: Treatment with systemic steroids
LSB: Treatment with local steroids.
TNF: Treatment with biological TNFα inhibitors.
AZA: Treatment with azathioprine.
Antibiotics: Treatment with known prescription drugs of antibacterial character.
Other: Treatment with rare IBD related drugs: 6-MP, MXT or Integrin antibodies.
Surgery: Whether the patient had surgical intervention.

Supplementary table 2:

	Regions of interest	Background regions
Overlap TF peak of interest	*A*: # region overlaps the selected TF peaks	*B*: # background region overlaps the selected TF peak
Do not overlap TF peak of interest	*C*: # region does not overlap TF peaks	*D*: # background region does not overlap TF peaks

Contingency table for calculation of ENCODE peak over-representation in regions of interest

Supplementary note 1:
Initial differential expression analysis including inactive patient samples.

Our initial hypothesis was that there is hidden information in the UCi and CDi samples that would help in distinguishing CD and UC. As described in the main text, this turns out not to be the case. To show this more clearly, we here include a version of an initial differential expression analysis, that was eventually discarded in favour of the approach currently included in the paper.

First, we made additional PCA analyses where we removed the inflamed samples to see possible additional patterns (Figure 2A shows the same analysis when all samples are present). Care should be taken in interpretation since PC distances are not comparable between different PCA plots if the samples in each plot are not the same. Below is the set of PCA-plots for TSSs and enhancers, split by sample type:

TSSs:

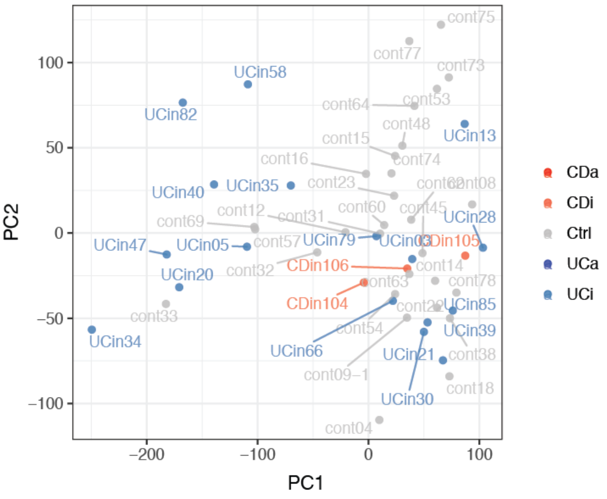

Enhancers:

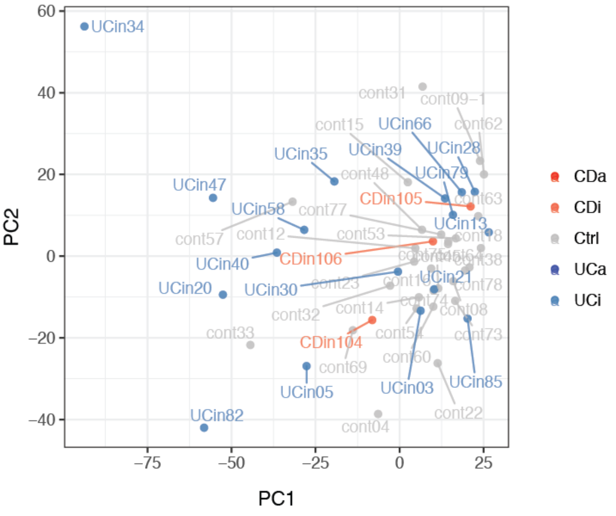

Our conclusion is that removing CDa and/or UCa does not improve the separation of controls from CDi and/or UCi, on either enhancer or TSS level.

Having established that there were no obvious subgroups in the inactive sets, we made an initial analysis that included

inactive samples. Here, we used a simple setup of the differential expression analysis: we performed all pairwise comparisons while correcting for batch effects. The results of such pairwise comparisons (performed using limma-voom) are shown in the barplot below: The X axis below shows the number of differentially expressed TSSs at FDR<0.05. Y axis show pairwise analyses.

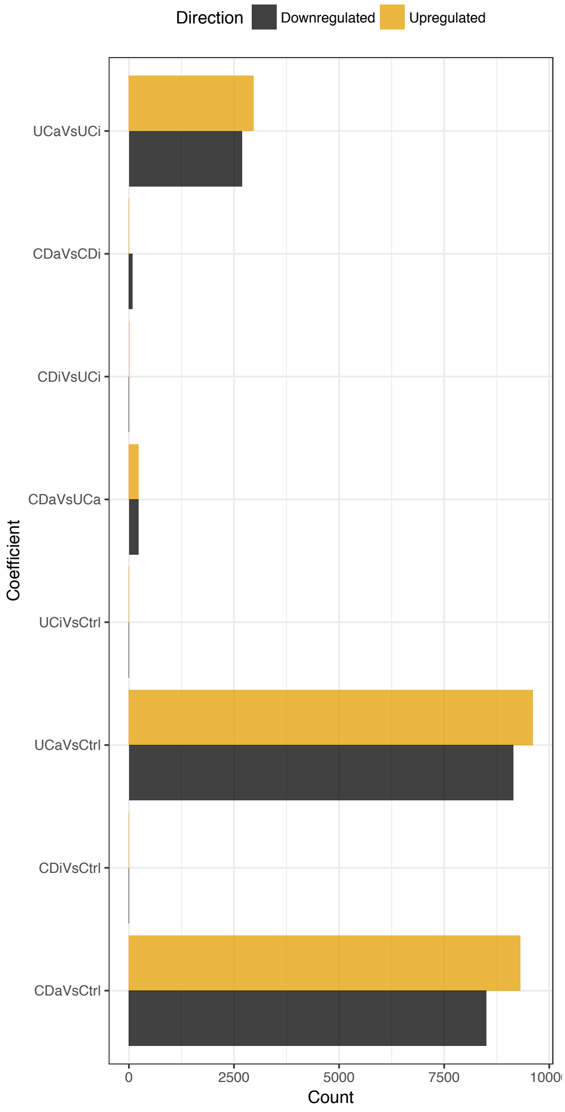

We can make several observations from this bar plot, all in agreement with the PCA plot from figure 2:

1. There is a very high number of differentially expressed TSSs in most comparisons between inflamed (CDa or UCa) vs. non-inflamed samples (CDi, UCi and Ctrl), as also seen in PC1 in Figure 2A.
2. There are very few (<12) differentially expressed TSSs between non-inflammed samples (any pairwise comparison between UCi, CDi and controls). This again reflects the similarity between UCi, CDi and Ctrl in the PCA plot in Figure 2A.
3. There is a modest amount of differentially expressed TSSs between CDa and UCa. This is also seen on PC2 in Figure 2A.

What can account for this very low amount of differentially expression in UCi/CDi vs Ctrl? CDi is most likely explained by the low sample size of n=3, resulting in very low power to detect anything but the highest fold changes. In the case of UCi, the PCA shows that this group seems to consist of a spectrum going from samples that are very similar to controls, and at the other extreme, somewhere between UCa and controls.

To see what comparisons that affect the same TSS(s), we used Venn Diagrams:

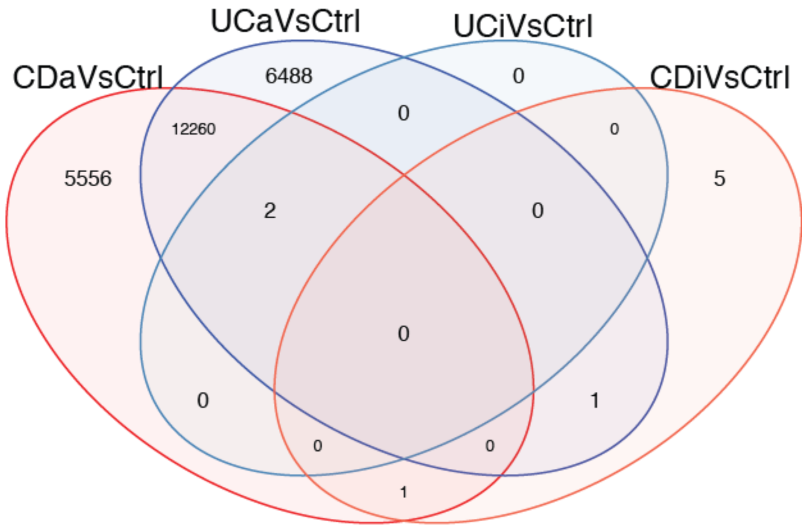

In this first comparison, where all groups vs Ctrl analyses are overlapped, we can see that a very large proportion of differentially expressed TSS are shared between CDa vs Ctrl and UCa vs Ctrl, showing that the two responses are similar, likely reflecting shared inflammatory response.

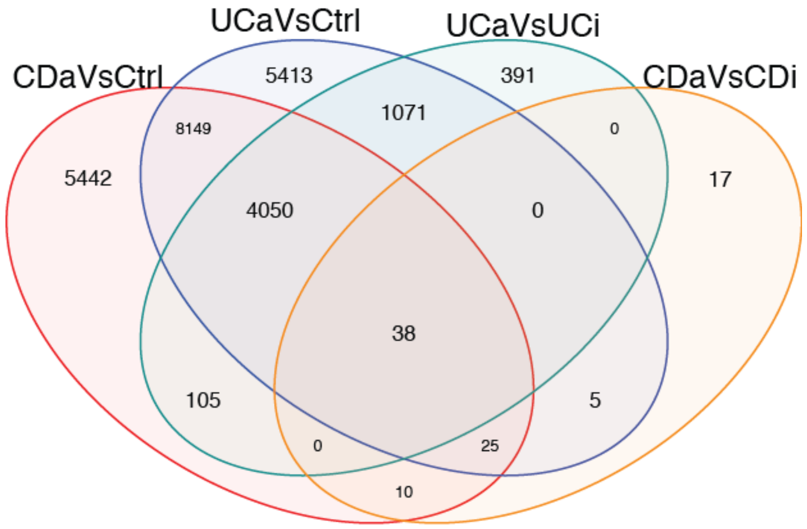

In this second analysis, where we compared active UC and CD vs Ctrl and inactive samples, we can see that although there is a substantial number of differentially expressed TSS between UCa and UCi (as also seen in the barplot above), the majority of these are also seen in UCa vs Ctrl, showing that these are just UCa-specific TSSs and that control and UCi are similar.

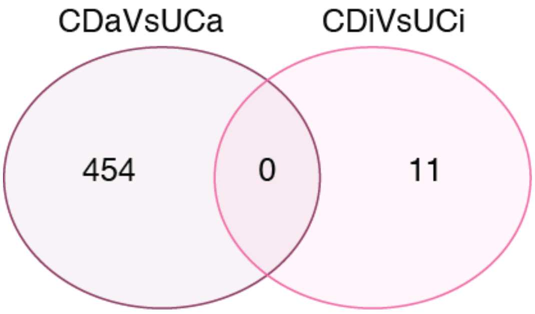

Finally, looking across comparisons between CD and UC samples, there is a far larger number of differentially expressed TSSs in the CDa vs UCa comparison than in the CDi vs UCi comparison, and no overlap between the two.

We interpreted these results as follows:

1. CDi has too low sample size to be of any real use in differential expression, or classification.
2. UCi is not informative enough to be modeled as a simple group. While it may be possible to model the response as a continuous variable (i.e. as PC1 in Fig 2A), this would make the model much more complex than the one we use in the paper, and it is prone to over-interpretation.

Because of this, in the paper, we chose to model this as a general inflammatory response (IBD_{up}/IBD_{down}) and weaker signal separating CD and UC. In statistical terms, we have two comparisons (contrasts):

1. IBD (IBD_{up}/IBD_{down}), which is equal to the mean of CD and UC vs control different from 0, and
2. CDvsUC, which is the difference between CDa and UCa and results in CD_{spec} and UC_{spec}.

These results also mean that a computational approach to distinguish CD from UC or controls will not be able to obtain much additional information from the UCi and CDi samples compared to UCa, CDa and control comparisons.

Representative histology images from control, UCa, CDa, UCi, CDi biopsies.

Images show representative histology of colonic pinch biopsies from (a) three controls (Ctrl), (b) three patients with Crohn´s disease (CDa), (b) three patients with ulcerative colitis (UCa), (f) one example of inactive CD (CDi) and one inactive UC (UCi). The hematoxylin and eosin stains presented were taken with a x40, x200 or a x400 lens. The scale bars represent 200 μm in the x40 pictures, 50 μm in the x200 pictures and 25 μm in the x400 pictures.

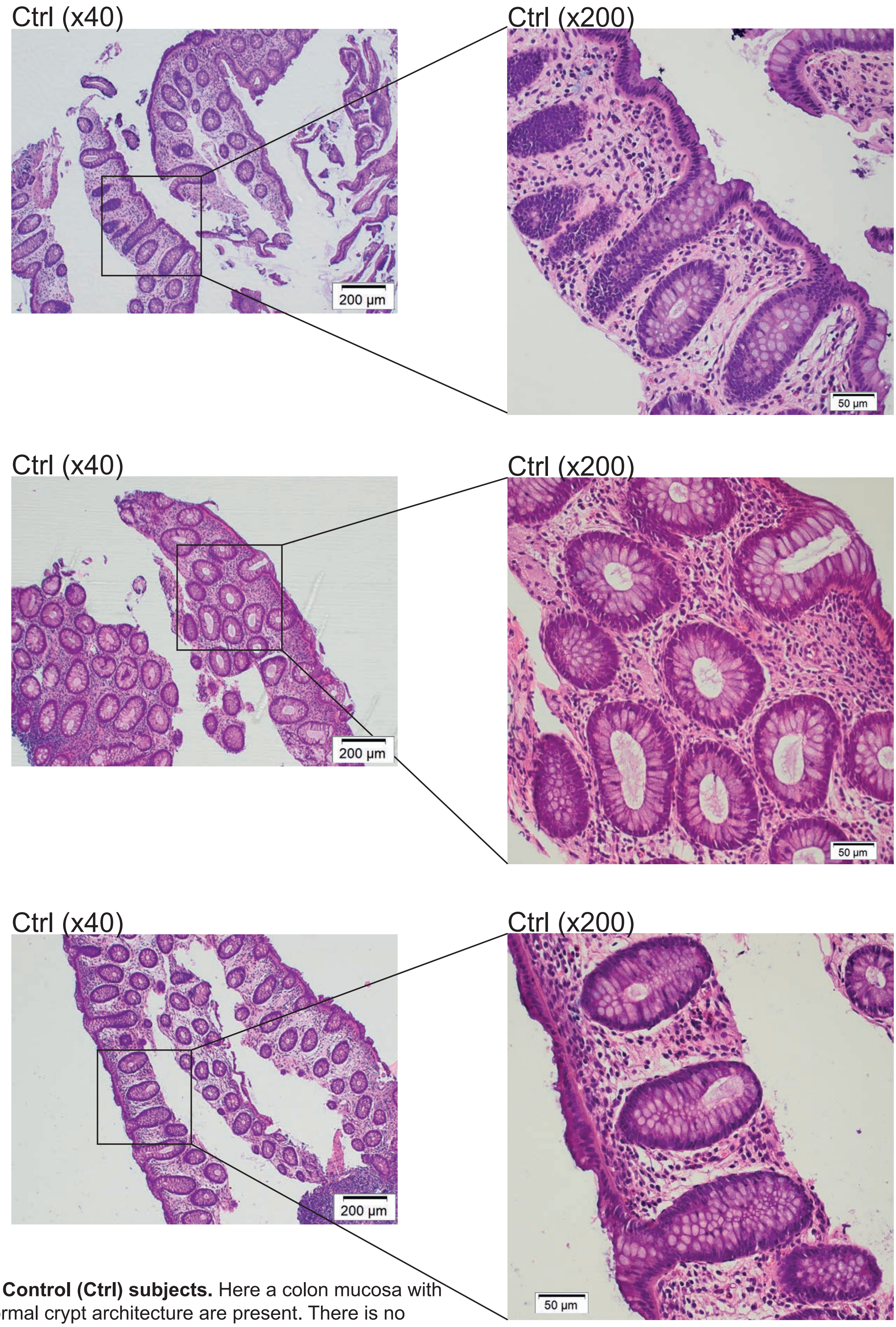

a: Control (Ctrl) subjects. Here a colon mucosa with normal crypt architecture are present. There is no acute or chronic inflammation in the epithelium or lamina propia.

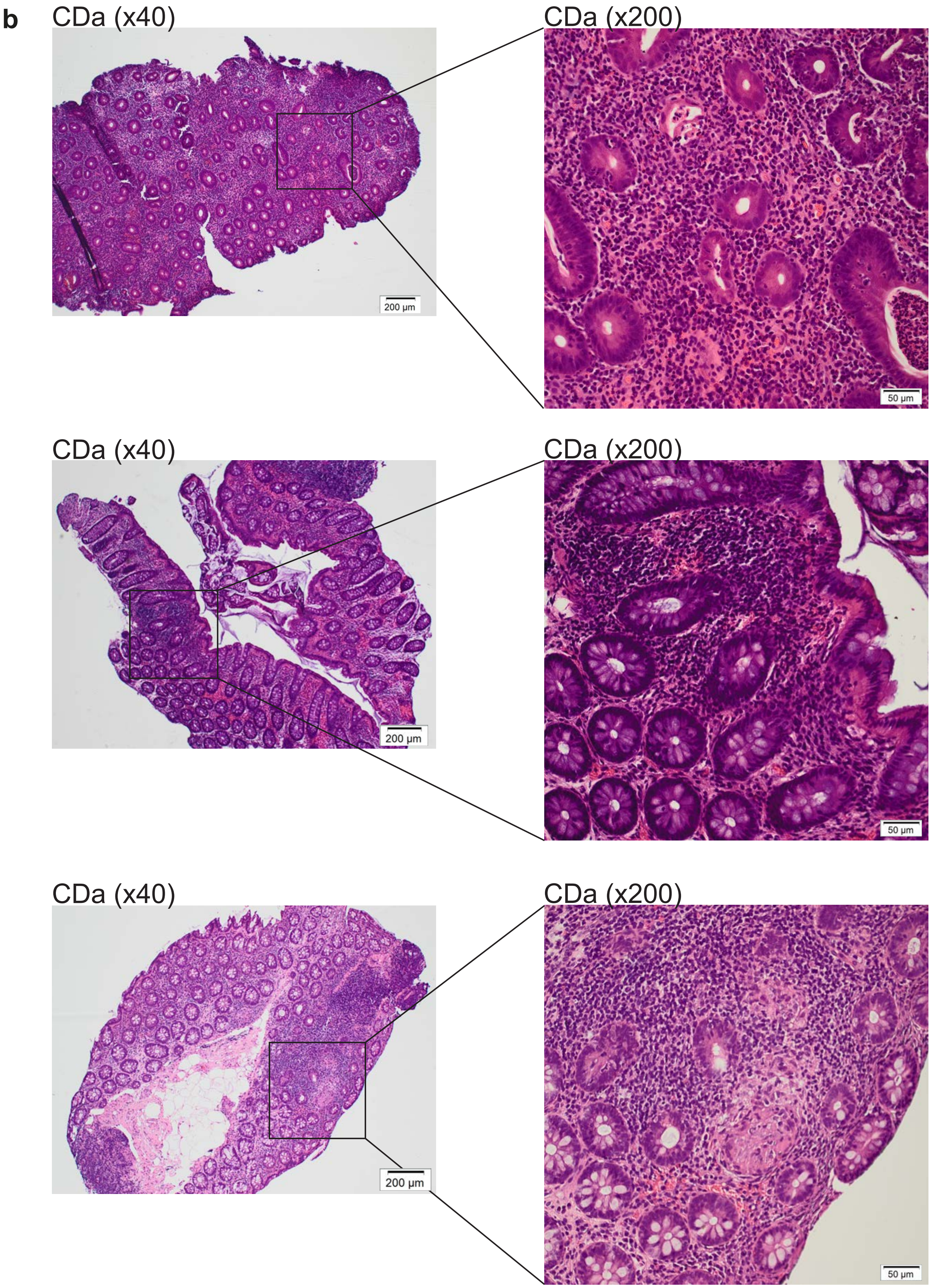

b: Active CD patients (CDa) . Here colon mucosa with chronic inflammation is present. In some biopsies, active inflammation is manifested by the presence of neutrophils in the epithelium. One biopsy contains a non-caseating epithelioid cell granuloma.

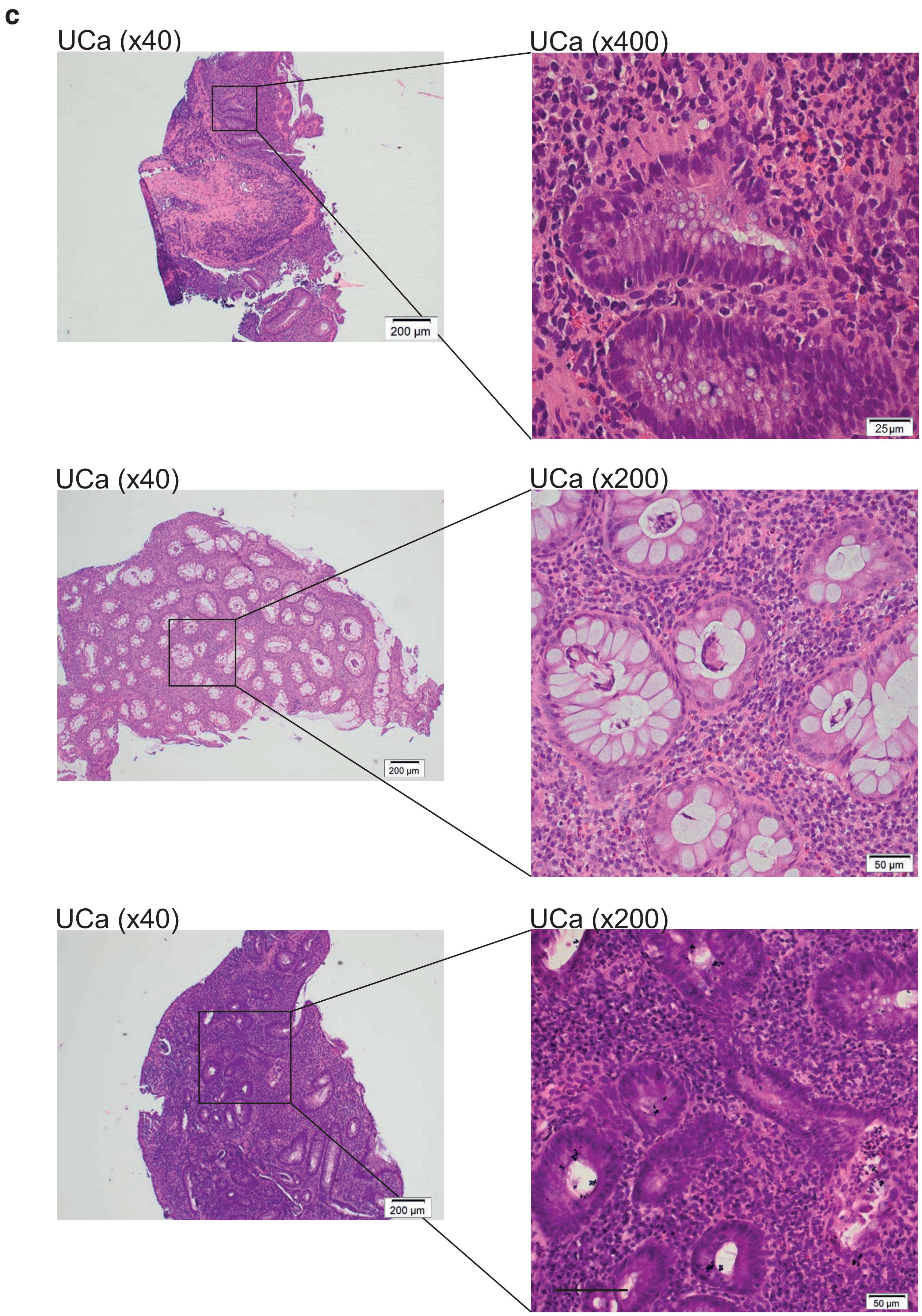

c: Active UC patients (UCa). Here a colon mucosa with active ulcerative colitis as present. There is crypt architecture distortion and a diffuse chronic inflammatory infiltrate in lamina propia with lymphocytes and plasma cells. Active disease reflected by the acute inflammation with cryptitis and crypt abscesses.

d

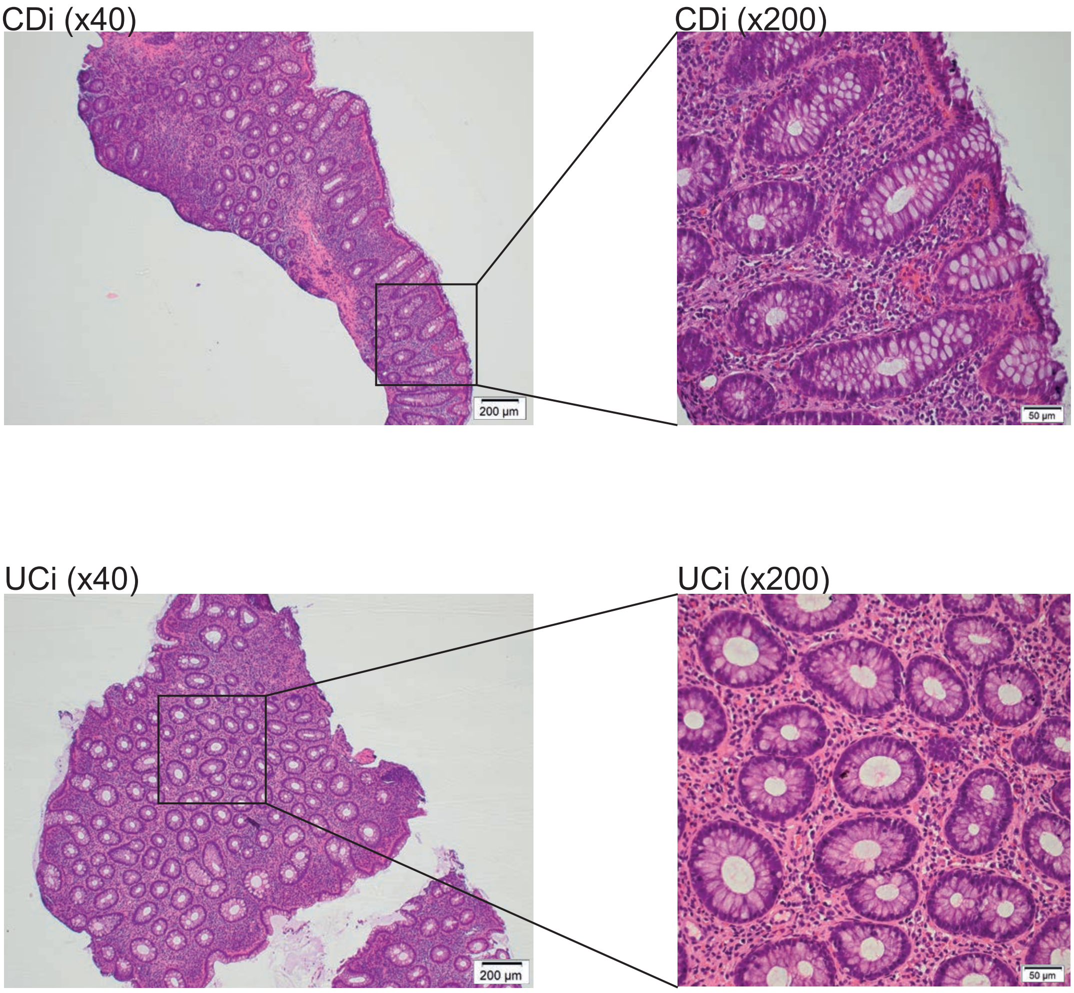

d: Inactive CD and UC patients (Ddi and UCi). Here a colon mucosa with normal crypt architecture and no acute or chronic inflammation are observed.

a

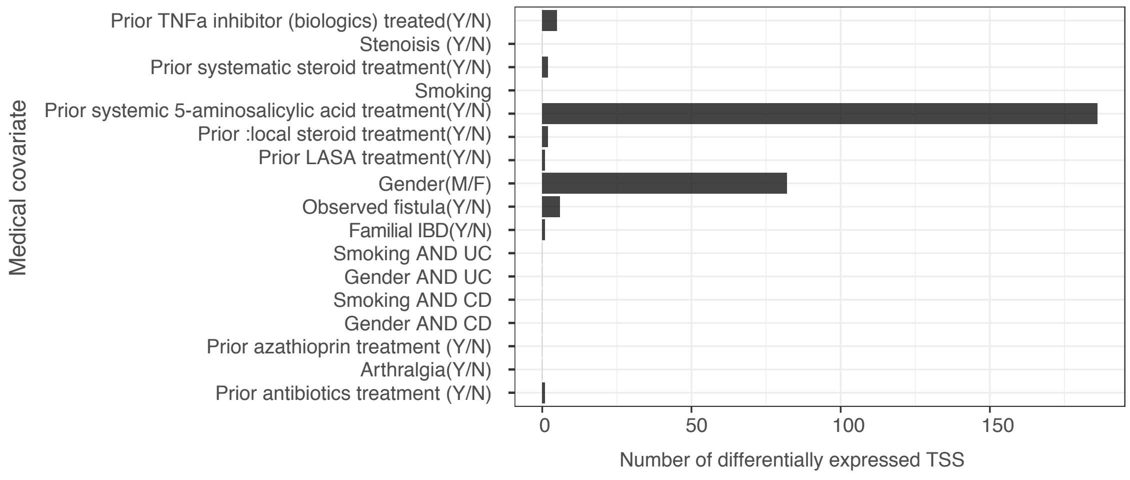

b

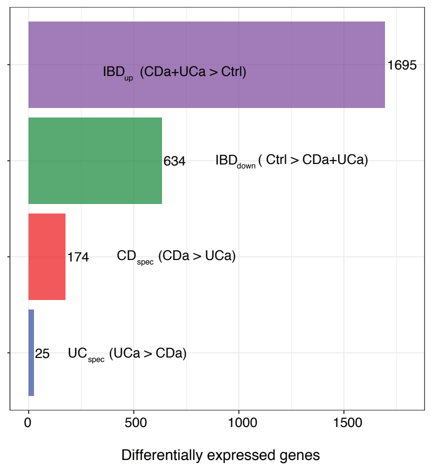

a: Impact of adding additional covariates on differential expression analysis.
limma-voom was used to fit TSS-wise linear models using additional medical covariates for subjects. Bars show the number of differentially expressed TSSs for each additional covariate. The largest effect is 5-ASA treatment (at around 175 differentially expressed TSSs) and gender (at around 75 differentially expressed TSSs), which is 1% or less compared to the 7320 TSSs that are differentially expressed when comparing diagnosis labels (CDa, UCa, Ctrl, as seen in Fig. 2b).

b: Number of differentially expressed genes
Bar plot shows the number of differentially expressed genes (by edgeR) in the four defined groups, as in Figure 2b, but evaluated by summing CAGE tags across gene models.

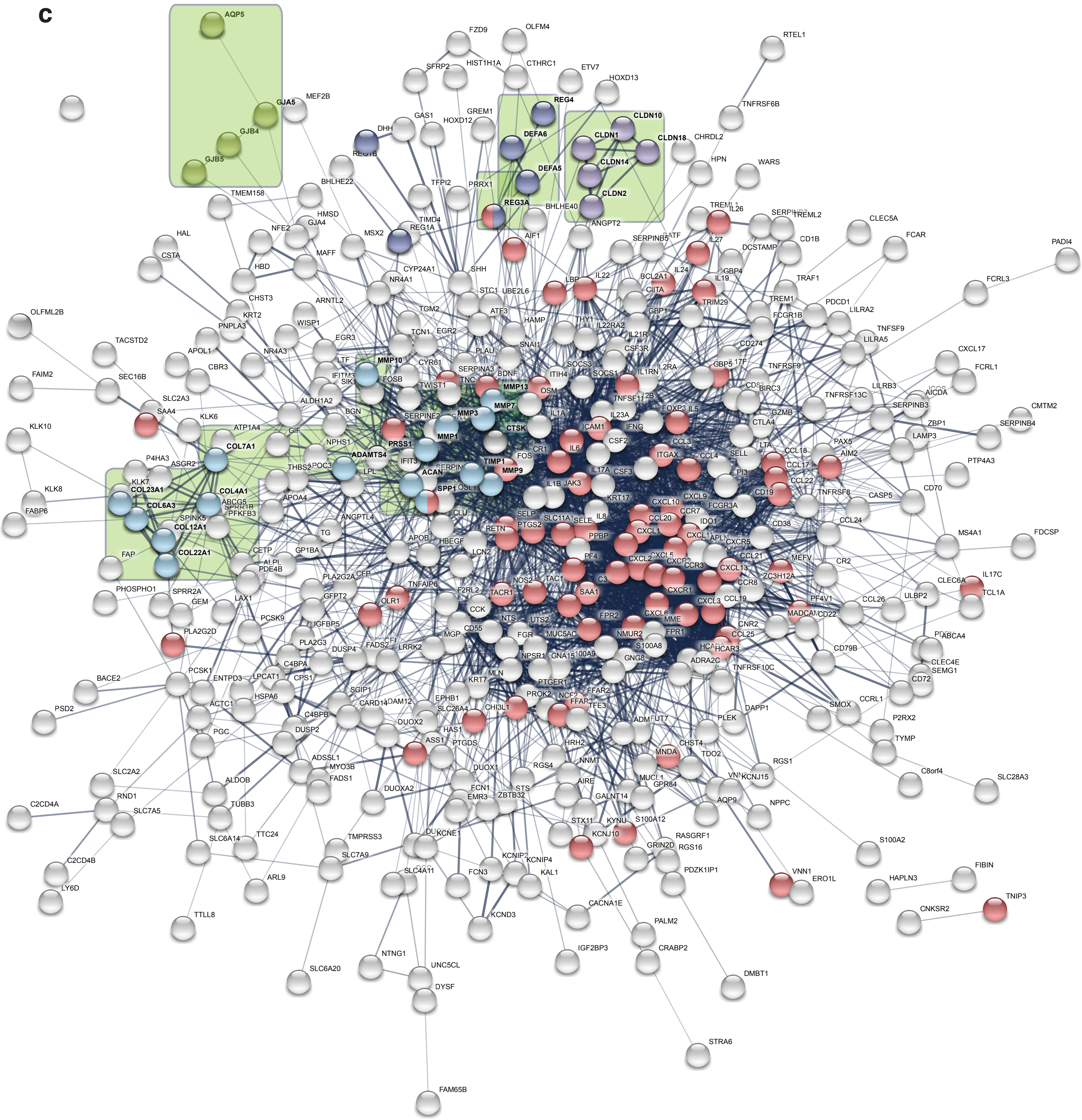

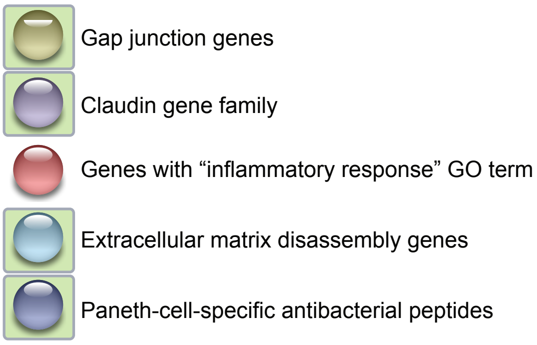

c: Protein-Protein interactions between IBD-upregulated genes
The STRING graph shows all IBD_{up} genes with at least one interaction at the additional requirement of an log fold change > 1.5 (to improve visibility of the network), where line width indicates the strength of interaction evidence. Genes involved in selected similar cellular functions are marked with colors.

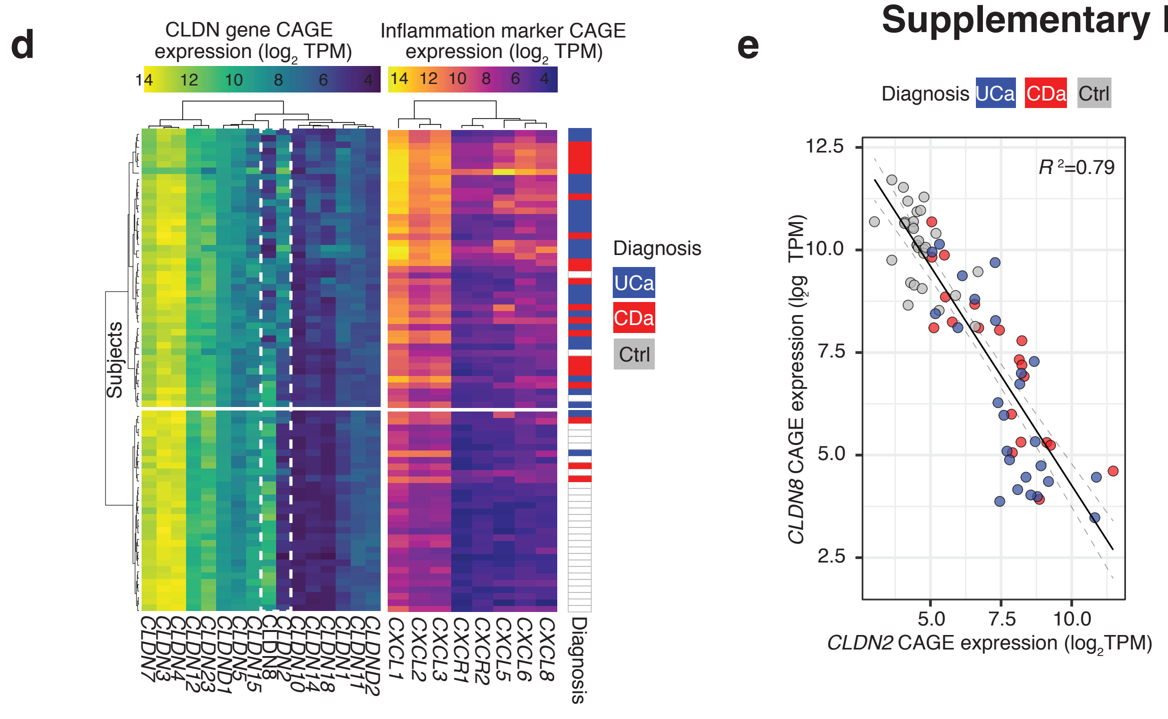

d: Analysis of CLDN gene expression
Left heatmap shows the expression of CLDN genes. Columns correspond to genes, rows to subjects. Right heatmap shows the CAGE expression of a set of cytokines and cytokine receptors, serving as inflammatory markers. Rows of the left and right heatmap clustered by the expression values of the left heatmap. The last two columns show subject diagnosis (CDa, UCa or Ctrl). Note that CLDN gene expression can distinguish IBD (CDA and UC) and Ctrl samples, and two genes (CLDN2 and CLDN8) were anticorrelated (see panel e).
e: Relation between CLDN2 and CLDN8 expression.
X-axis shows the CAGE expression of CLDN2, Y-axis shows CLDN8. Dots are colored by subject diagnosis as indicated. A linear regression (solid line) with standard errors (dashed lines) and the R^2 statistics (upper right corner) are superimposed.

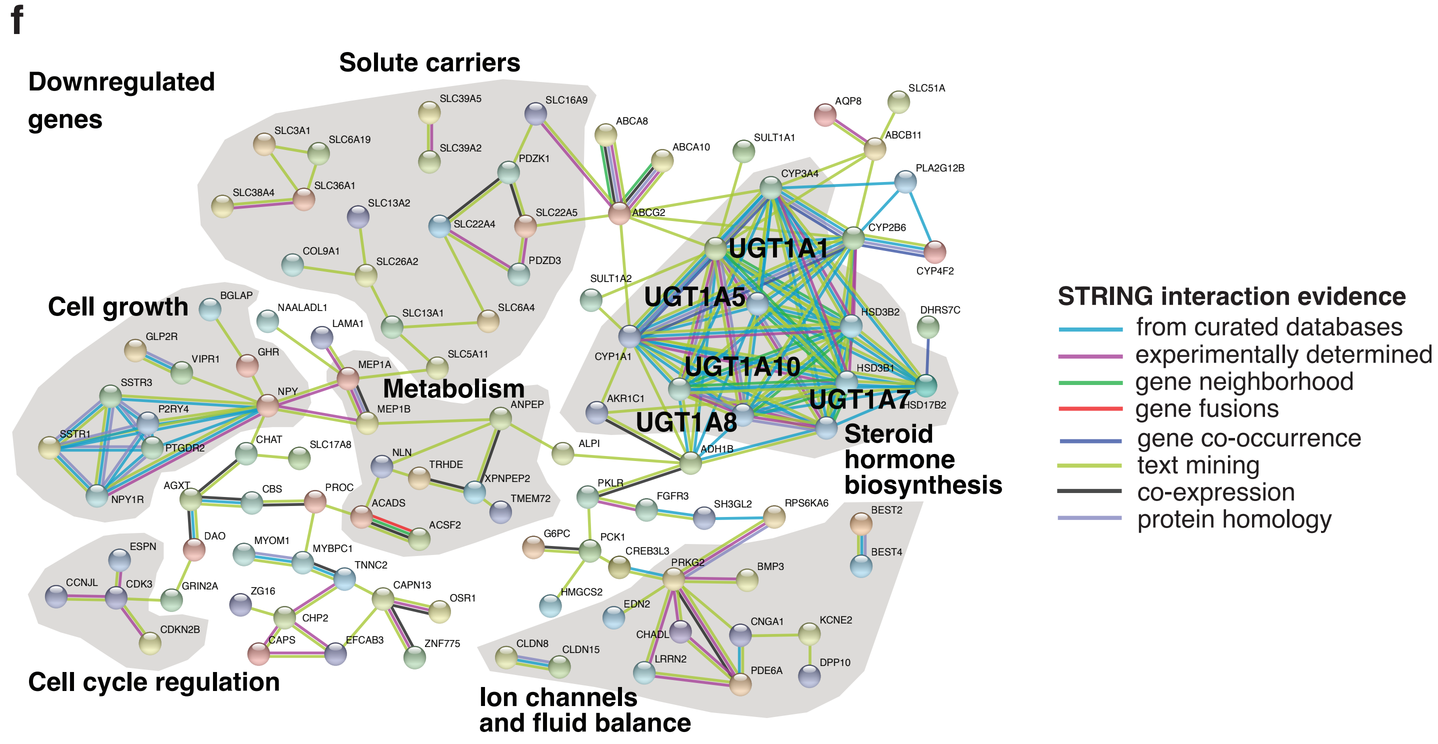

f: Protein-Protein interactions network between IBD-downregulated genes
The STRING graph shows all IBDdown genes with at least one interaction, where line width indicates the type of interaction evidence. Genes involved in similar cellular functions are marked with grey shading.

g

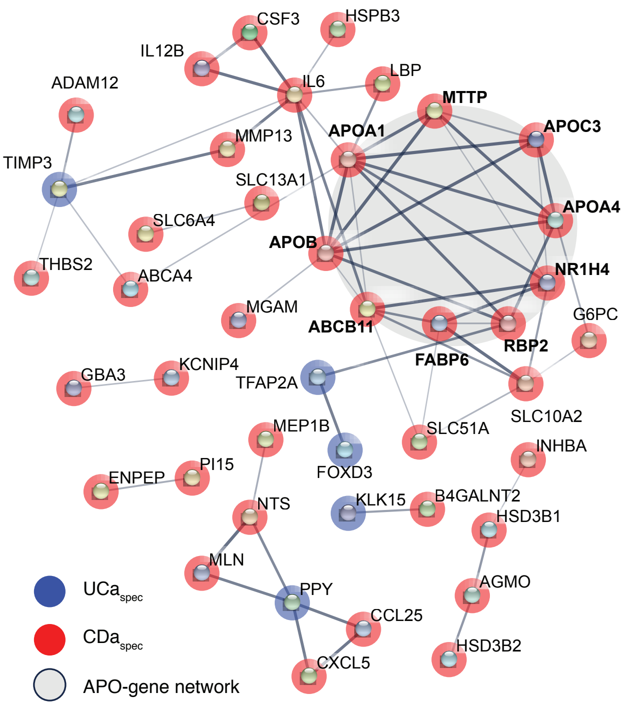

g: Interaction map of genes differing in expression between UC and CD.
All CD_{spec} and UC_{spec} genes were subject to The STRING graph shows all genes in CD_{spec} and UC_{spec}, where unconnected nodes are not shown and line width indicates strength of interaction evidence. Red and blue highlights correspond to gene membership to CD_{spec} and UC_{spec} groups, respectively. Genes involved in lipid handling or chylomicron formation are indicated in boldface with grey background.

h

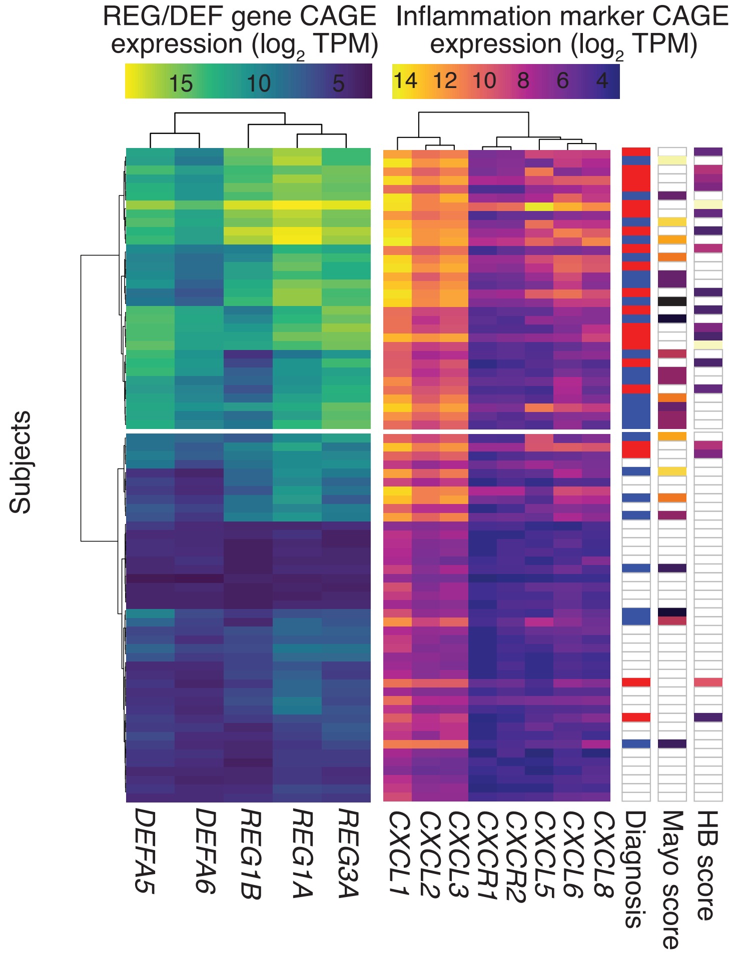

h. Expression of antibacterial peptide genes.

Left heatmap shows the expression of the antibacterial genes identified in Fig.2d, as log_2 CAGE TPM. Columns correspond to genes, rows to subjects. Right heatmap shows the CAGE expression of a set of cytokines and cytokine receptors, serving as inflammatory markers. Rows of the left and right heatmap are ordered by the left heatmap are clustered by the expression values of the left heatmap. The last two columns show subject diagnosis (CDa, UCa or Ctrl), and disease severity scores for each patient, Mayo scores for UCa and HB scores for CDa.

Supplementary Figure 3

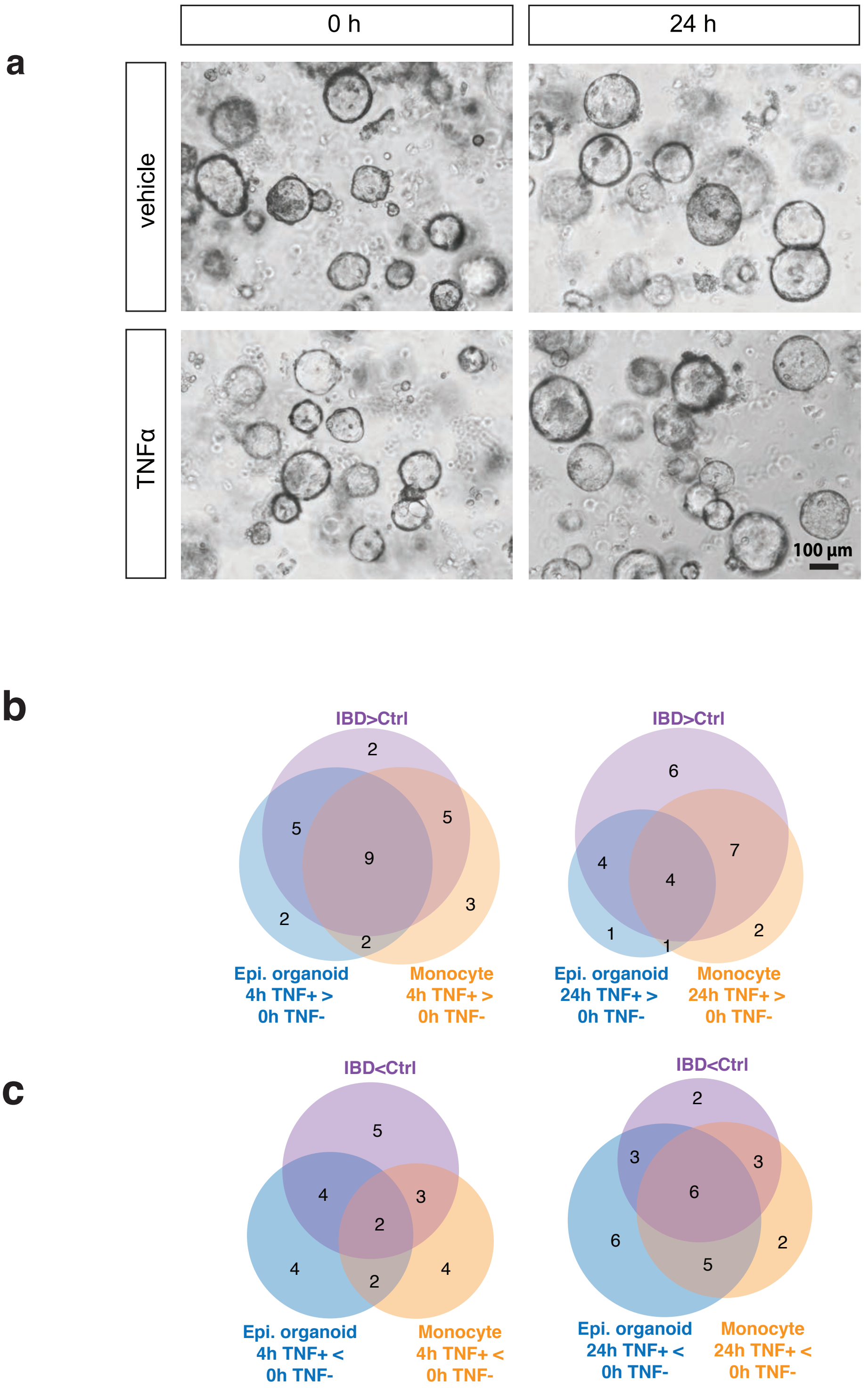

a: TNFA(TNFα) treatment of human colonic organoids. Images shown are representative examples of colonoids cultured in 3D hydrogel domes with or without TNF. Organoid cultures were treated for up to 24 hours without causing gross morphological changes.
b: Correspondence of TSSs upregulation in IBD to TNF induction in epithelial organoids and blood monocytes. Venn diagram shows the number of TSSs (out of 36 tested by qPCR) that are upregulated in IBD, upregulated after TNF treatment in gut epithelia organoids or blood monocytes, after 4h or 24h.
c: Correspondence of TSSs downregulation in IBD to TNF–induced downregulation in epithelial organoids and blood monocytes. Venn diagram shows the number of TSSs (out of 36 tested by qPCR) that are downregulated in IBD, downregulated after TNF treatment in gut epithelia organoids or blood monocytes, after 4h or 24h.

a

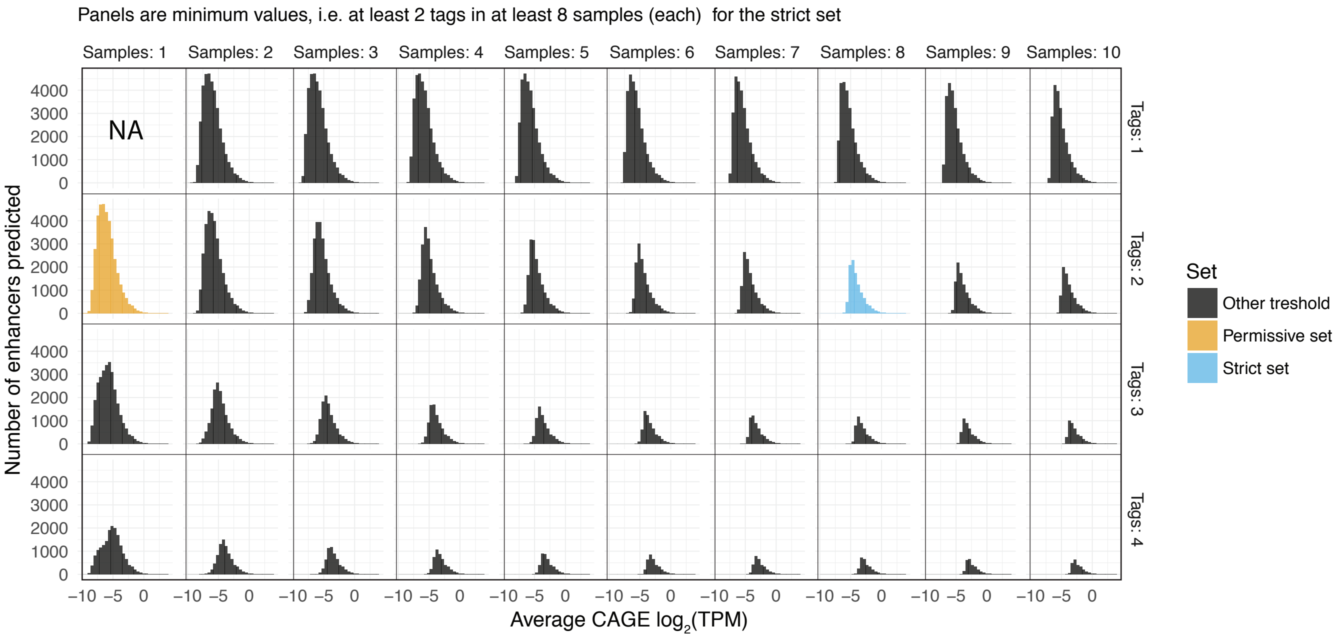

a. Thresholding effects in enhancer detection. Each panel shows histograms of detected enhancer counts (Y axis) for a given CAGE expression (average $\log_2$(TPM), X axis). Panels represent different combinations of cutoffs on tags and samples. Rows define minimum number of tags required and columns define the minimum number of samples in which this number of tags have to be present . The strict set (at least 2 tags in 8 samples, each) is shown in blue and the permissive set (at least 2 tags in at least 1 sample) is shown in yellow. The combination 1 tag in at least one samples is not used even in the permissive set because all enhancers are required to be bidirectional in at least a single sample which requires a minimum of two tags. The corresponding panel is therefore shown as "NA".

b

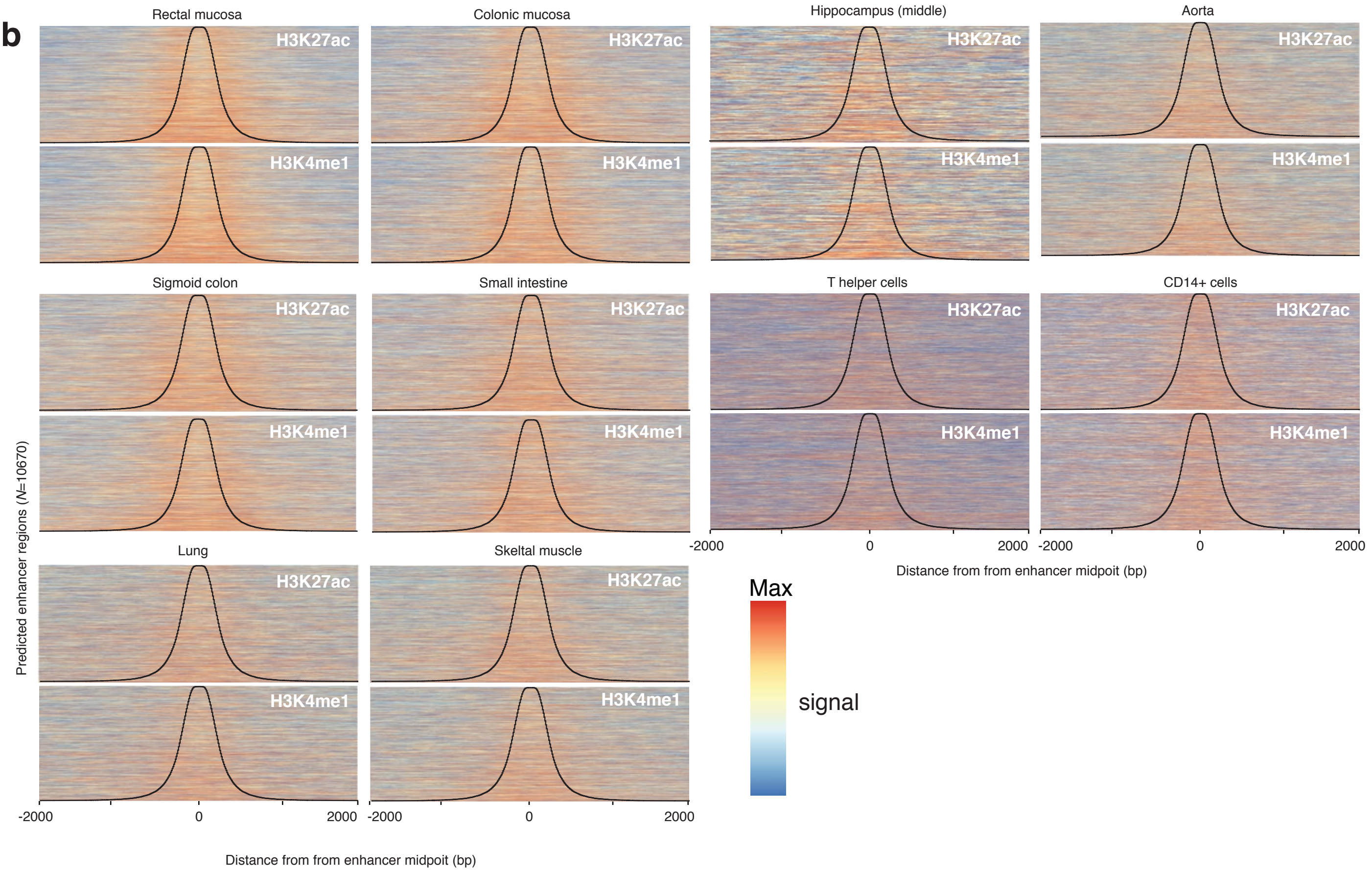

b. H3K27ac and H3K4me1 ChIP-Seq enrichment within enhancer regions identified by CAGE in gut biopsies. This image expands Figure 3c and is organized in the same way, but with additional ChIP-seq adta from several tissue samples (The Roadmap Epigenomics Consortium et al. 2015)

c

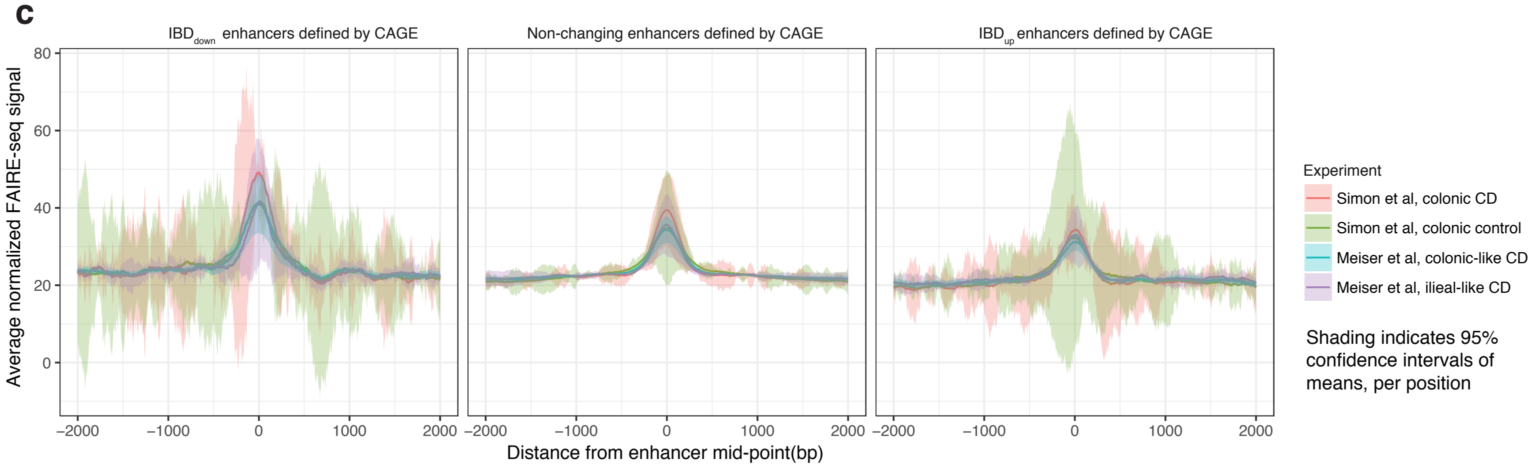

c: FAIRE-seq enrichment within enhancer regions identified by CAGE in gut biopsies. The same regions as above, but split by differential expression (IBD$_{up}$, IBD$_{down}$ and non-changing enhancers) were overlaid by FAIRE-seq data from colonic samples from two studies: Simon et al 2016 (two control and two CD biopsies) and Meiser *et al* 2016 (Only CD samples, split up by the authors as colonic-like(N=11) and ileum-like samples (N=9)). Y-axis show the mean FAIRE-seq signal, divided into subsets in respective study (colored lines). Shading indicates 95% confidence intervals. X-axis show the distance to enhancer midpoints.

d

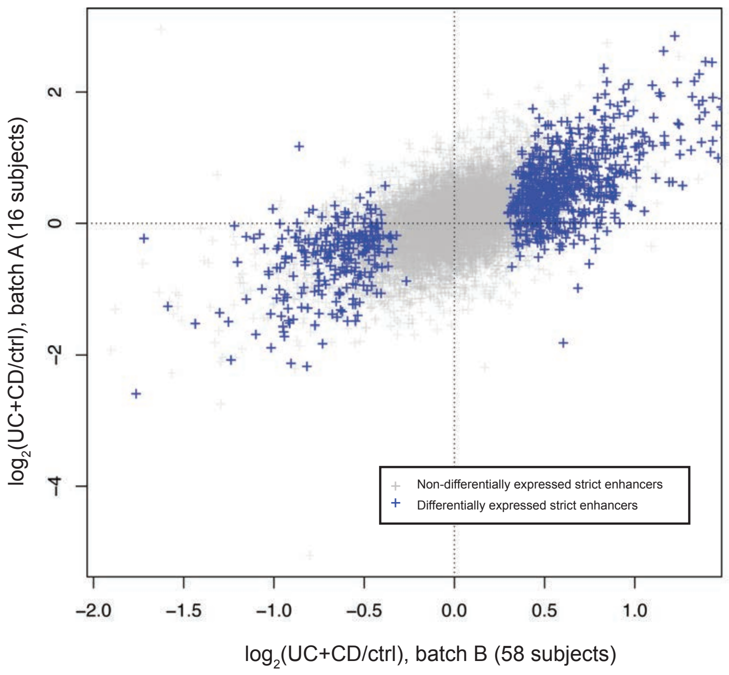

d Comparison of enhancer expression in batches A and B. X and Y axes show log$_2$ fold changes between CD and UC vs Ctrl estimated from batch B and A, respectively. Each cross corresponds to one enhancer region. Blue crosses correspond to enhancers that were found to be differentially expressed (either IBD$_{up}$ or IBD$_{down}$) with 90% posterior probability, assessed from batch B. Note how the sign of the log fold change is in agreement between the two batches for most of the differentially expressed enhancers (blue crosses).

Supplementary Figure 5

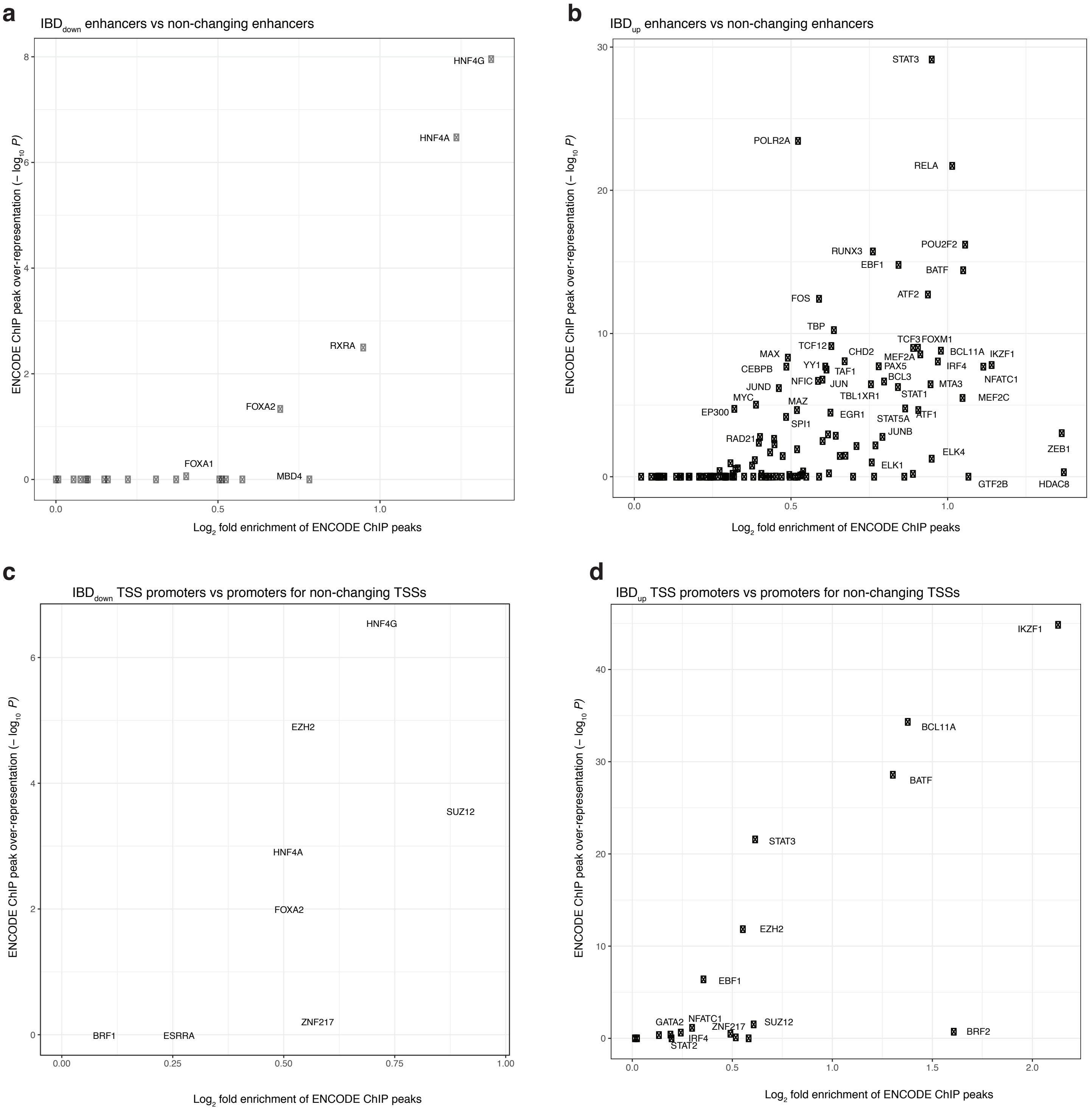

Over-representation of ENCODE transcription factor ChIP-seq peaks in regulatory regions

a: Over-representation of ENCODE transcription factor ChIP-seq peaks in the IBD$_{down}$ enhancer set vs. non-changing enhancers. X-axis shows the over-representation fold change, where 0 indicates no difference compared to background. Y-axis shows corresponding P value in –log10 scale (Fishers exact test). Each dot corresponds to one set of TF peaks. Selected dots are labeled by TF name.

b: Over-representation of ENCODE transcription factor ChIP-seq peaks in the IBD$_{up}$ enhancer set vs. non-changing enhancers, organized as in panel a.

c: Over-representation of ENCODE transcription factor ChIP-seq peaks in promoters for the IBD$_{down}$ TSS set vs. promoters of non-changing TSSs, organized as in panel a.

d: Over-representation of ENCODE transcription factor ChIP-seq peaks in promoters for the IBD$_{up}$ TSS set vs. promoters of non-changing TSSs, organized as in panel a.

Supplementary Figure 6

Enhancer and TSS annotation of the cytokine-cytokine receptor pathway.

The poster depicts a modified version of the KEGG pathway “Cytokine-cytokine receptor interaction” Each gene holds the following information in its own panel:

i) Gene name as defined in KEGG (at a few instances, KEGG does not use official gene names). Gene name color for differential expression (purple indicates IBD_{up} set, green IBD_{down} set, grey indicates no differential expression).

ii) Number of linked enhancer regions, indicated by the number left of the small circle “E”.

iii) Number of TSSs, counting only detectable CAGE clusters, indicated by the number close to “TSS”

iv) Summary of average CAGE expression in log2 scale in UCa, CDa and Ctrl groups as bar plots

Grey lines indicate if the protein encoded by respective gene is located in the cell membrane. Black lines show the cytokine-cytokine receptor interaction as in KEGG.

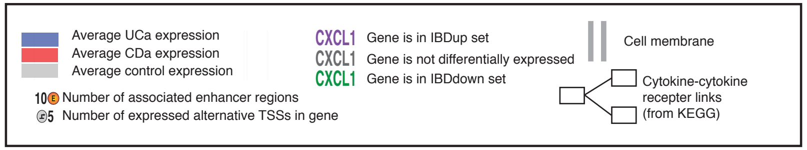

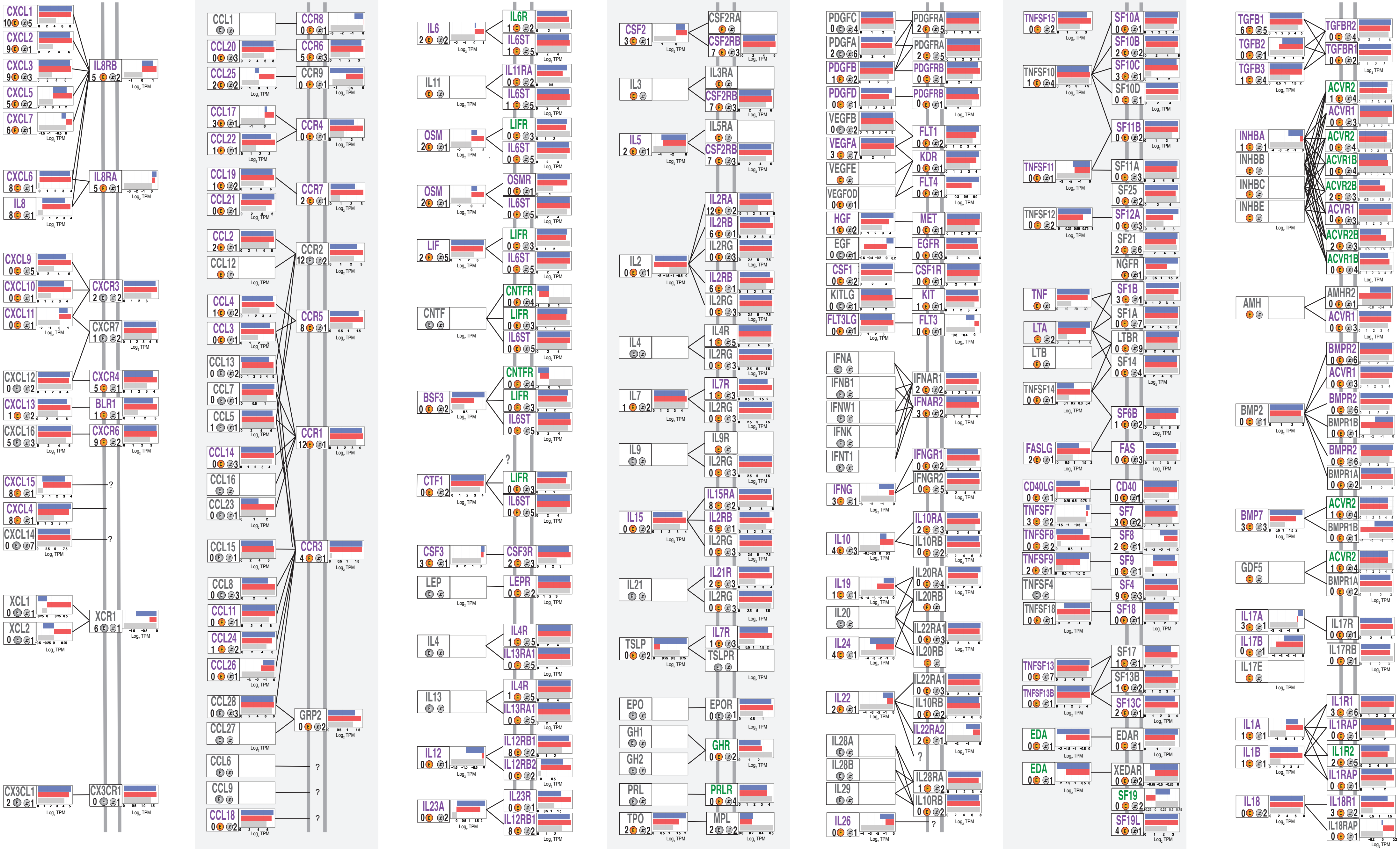

a

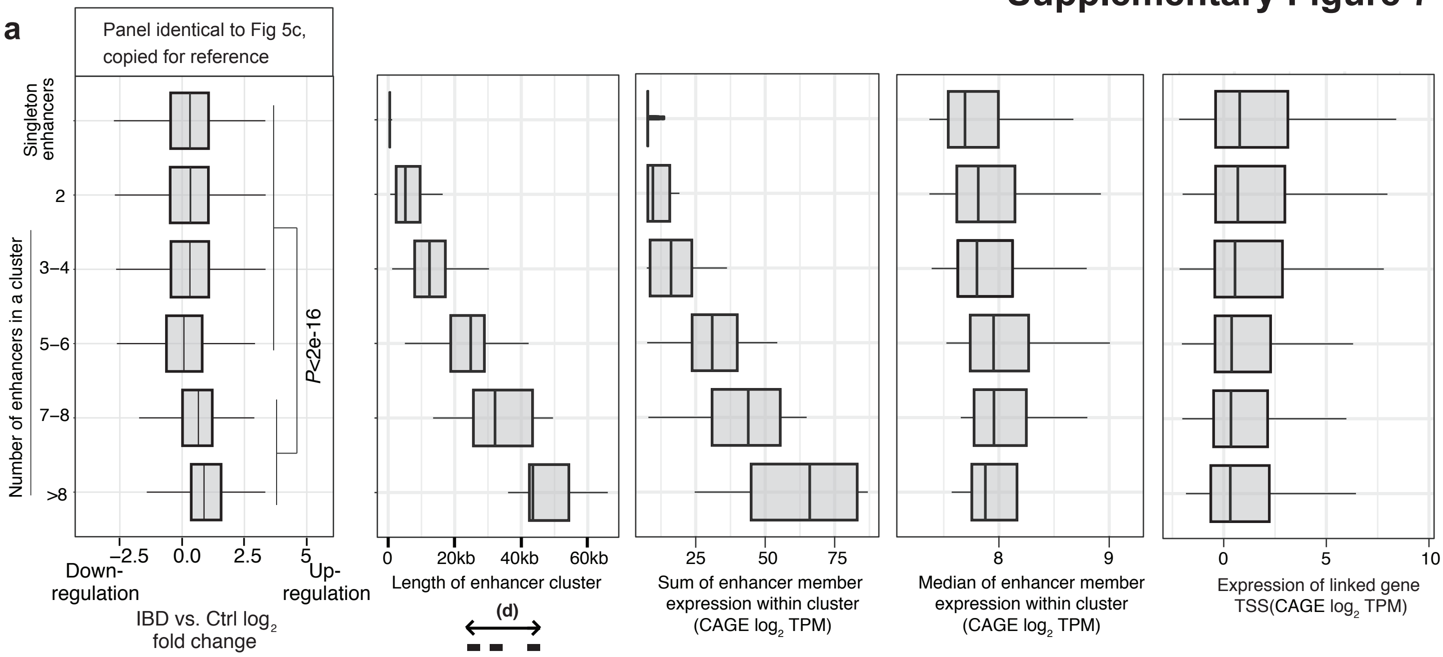

a: Extended co-variate analysis of the relation between the number of enhancers (Y axis) within enhancer clusters vs. other features (x axis), extending Fig. 5c. The first panel from the left is identical to Fig. 5C and included for reference, showing the distribution of IBD vs. Ctrl log_2 fold changes of TSSs linked to singleton enhancers or enhancer clusters as in panel 5B.Remaining panels show distributions of : lengths of enhancer clusters (distance from most 5' to most 3' enhancer edge), summed TPM expression across all libraries across all enhancers in clusters, median TPM expression across all libraries across all enhancers in clusters, and expression of linked gene TSSs.

b

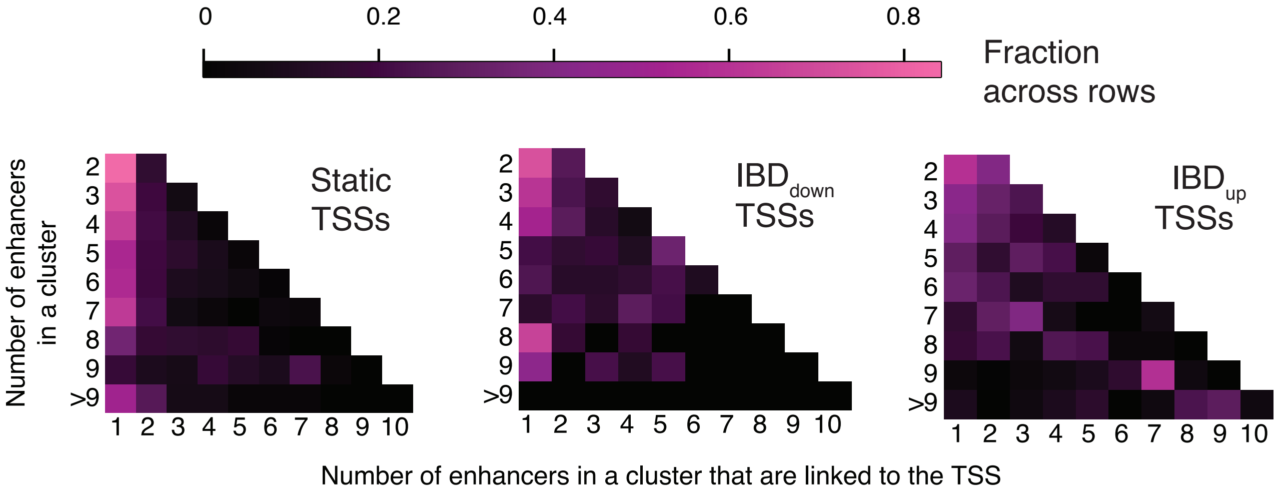

b: Linkage between TSSs and individual members from enhancer clusters as a function of number of enhancers in cluster. Heatmaps for TSSs that are not changing, down-regulated or up-regulated in IBD are shown. Heatmap rows indicate number of enhancers within analyzed enhancer clusters. Columns indicate the number of enhancers that are linked with the TSS. Cell color indicates the fraction of cases across rows.

Supplementary Figure 8

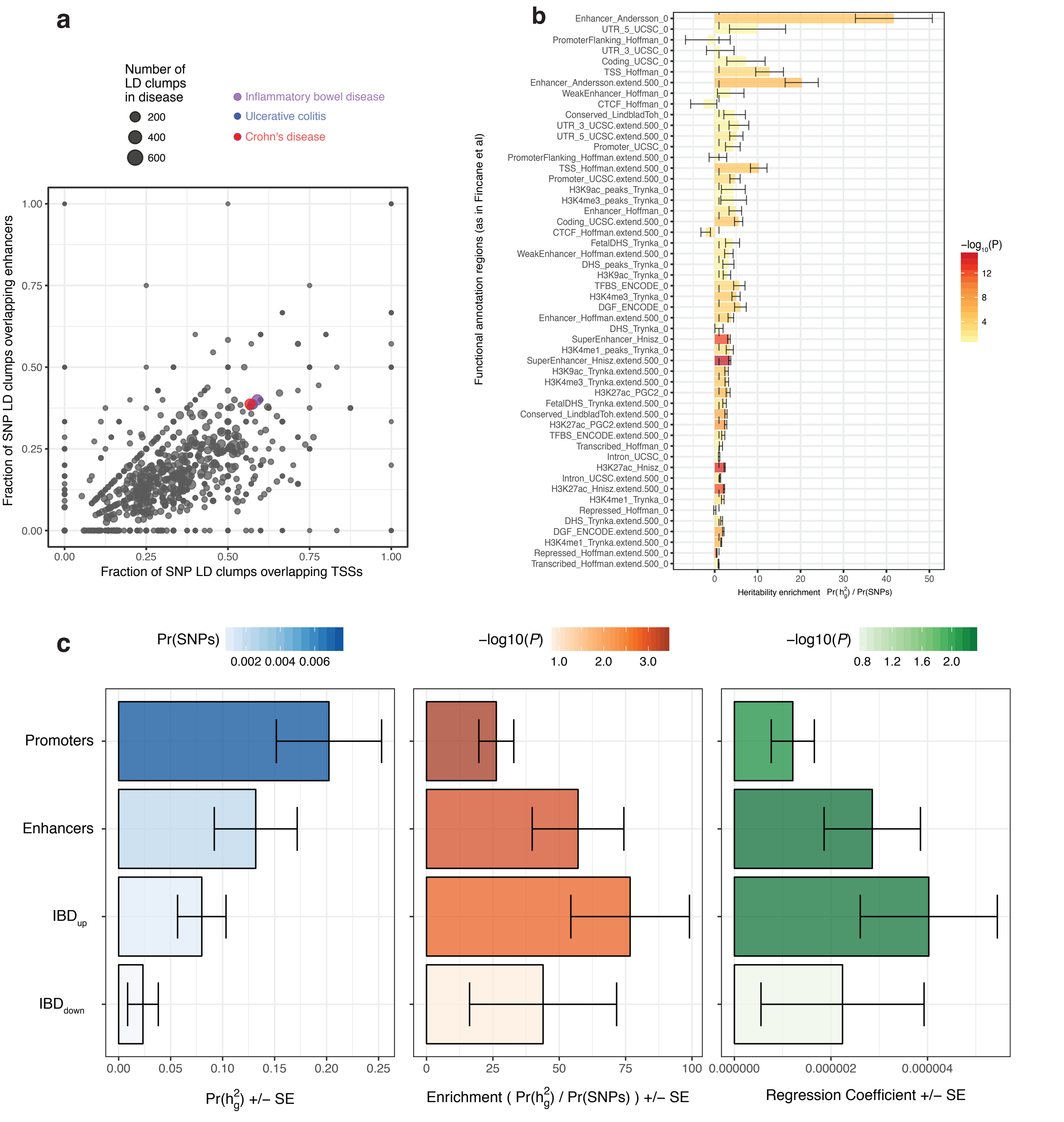

Relation between GWAS and enhancer and promoter regions in IBD
a. Overlap of GWAS catalog diseases and traits with identified TSSs and enhancers. Plot is organized as in Fig. 6a, but without using empirical Bayes shrinkage. Note the tendency that some categories that have a single or very few LD-clumps have relatively high proportions, which is corrected for in Fig.6a.
b. Partitioned heritability of IBD as estimated by stratified LD-score regression for each class of functional annotations. Heritability scores using the IIBDGC GWAS summary statistics for the functional categories included in the baseline model of Fincane et al. X axis shows enrichment of heritability , with jackknife standard errors indicated by whiskers. Y axis is functional annotations sorted by Pr(SNPs).
c. Sequentially adding IBD-specific regions to the baseline model. Left: Bar plot shows the proportion of heritability for each category (as in Fig.6d) relative to their genomic size (Pr(SNPs)) as indicated by color. Middle panel: Bar plots show absolute enrichment of heritability , with color scale indicating P-value. Whiskers indicate jacknifed standard error. Note that despite covering a small fraction of the genome, enhancers, TSSs and IBD_{up} are highly enriched for IBD heritability. Right: Bar plots show relative enrichment of heritability when controlling for the effect of all categories in the genomic baseline model, measured as the absolute value of the regression coefficient of the model, where color scale indicates P-value. Whiskers indicate standard error. Controlling for the effect of all categories in the genomic baseline model TSSs, enhancers and IBD_{up} provide additional information to model.

a

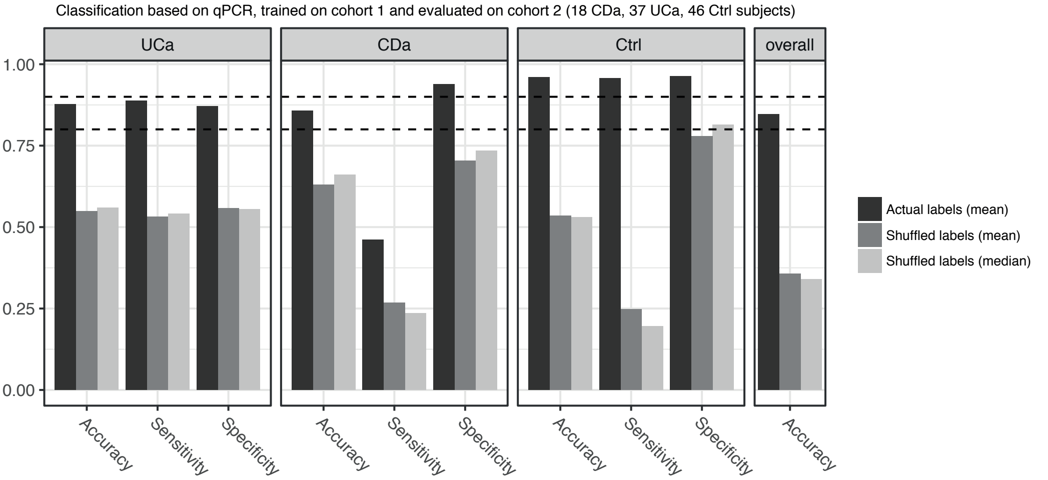

b

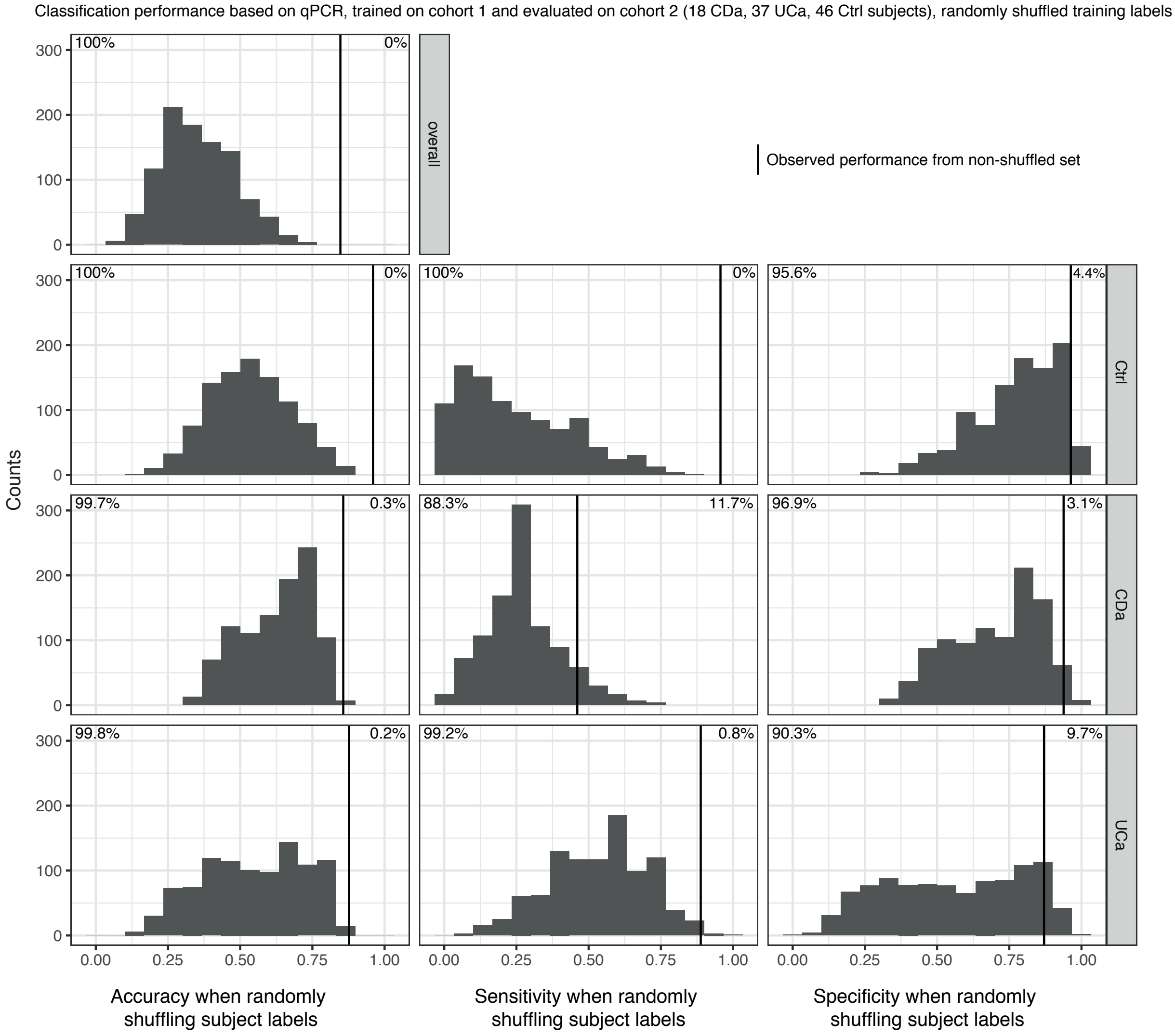

Classification performance
Plots a-b expand Fig.7c and compare classification performance on cohort 2 when Random Forests were trained on cohort 1 data with actual subject labels vs. when training labels were randomly shuffled. The training and prediction, based on randomly shuffled labels, were repeated 1001 times.
a: Average performance. Average accuracy, sensitivity and specificity are shown for each subject group along with the overall accuracy. Black bars show the mean accuracy of our predictions using actual data labels. Mean and median accuracy, sensitivity and specificity from prediction based on shuffled training data are also shown (grey bars).
b: Classification performance distributions. Distributions of accuracy, sensitivity and specificity (columns) for each subject group (rows) for the 1001 predictions after training on shuffled labels, expanding the summary statistics in panel a. Black vertical lines indicate performance on actual (non-shuffled) labels. The percentage of shuffled results that is smaller or larger than the results using actual labels for individual analyses is indicated in corners of subplots

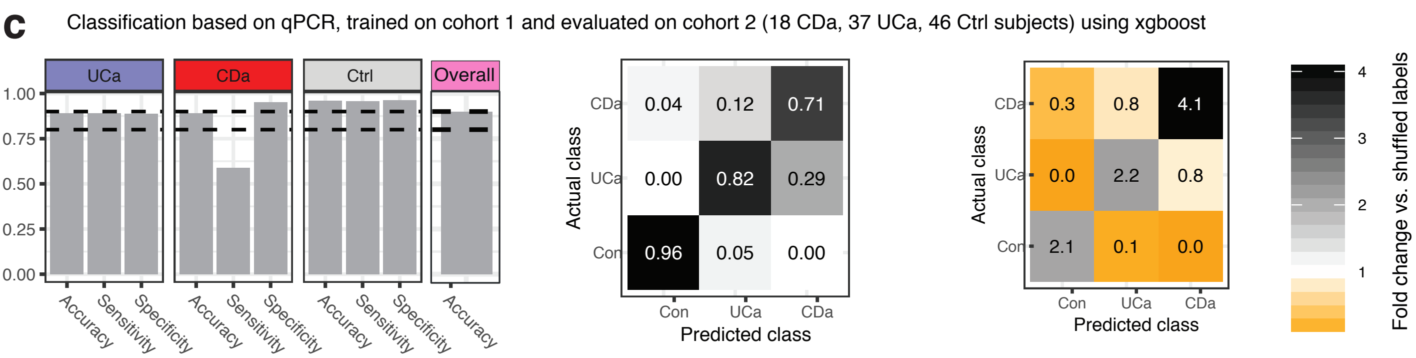

c. Validation of the prediction method in an independent cohort using xgboost
Panels and analysis as in Fig 7d: analysis is identical but using XGboost instead of Random forest as a prediction framework.

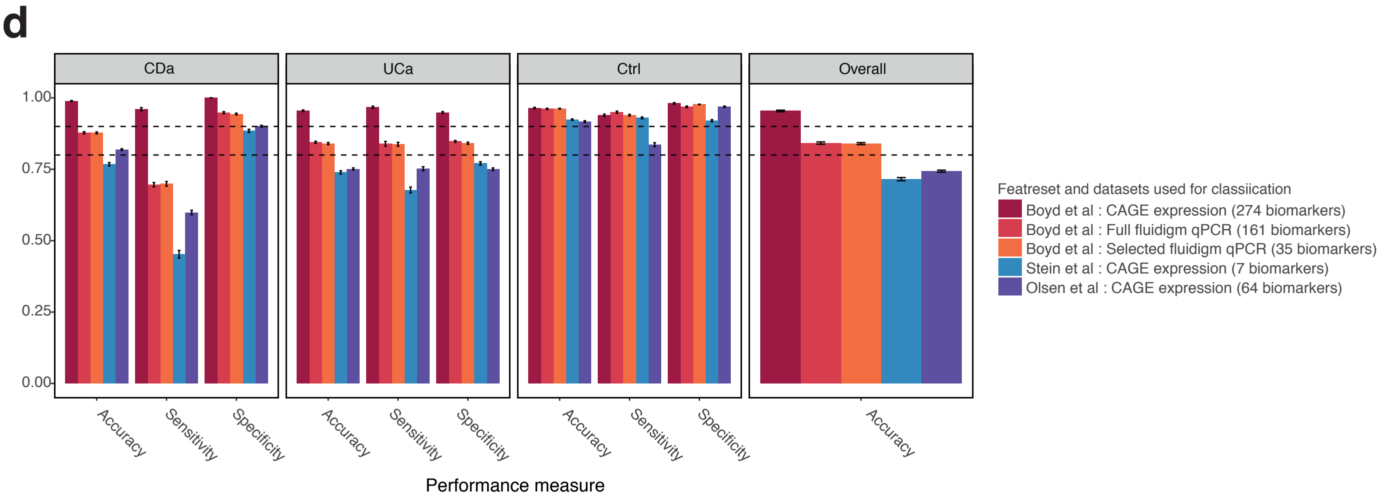

d: Comparison of classification performance between our and external biomarker sets
Stein *et al* 2009 and Olsen *et al* 2008 defined sets of genes which may be able to classify UC, CD and controls. We measured the expression of these genes by summing CAGE tags across corresponding gene models in cohort 1. For each gene set, we trained and evaluated a Random Forest model using a 5-fold cross-validation scheme, a procedure we repeated 1000 times to ensure stable results. For comparison, we made the same analysis on i) our initial 274 CAGE-defined markers, measured by CAGE data ii) 161 Fluidigm targets, measured by qPCR in cohort1, iii) the final 35 Fluidigm targets measured by qPCR in cohort1. Average accuracy, sensitivity and specificity are shown for each subject group as bar plots along with the overall accuracy, as in Figure 7.

Supplementary Figure 10

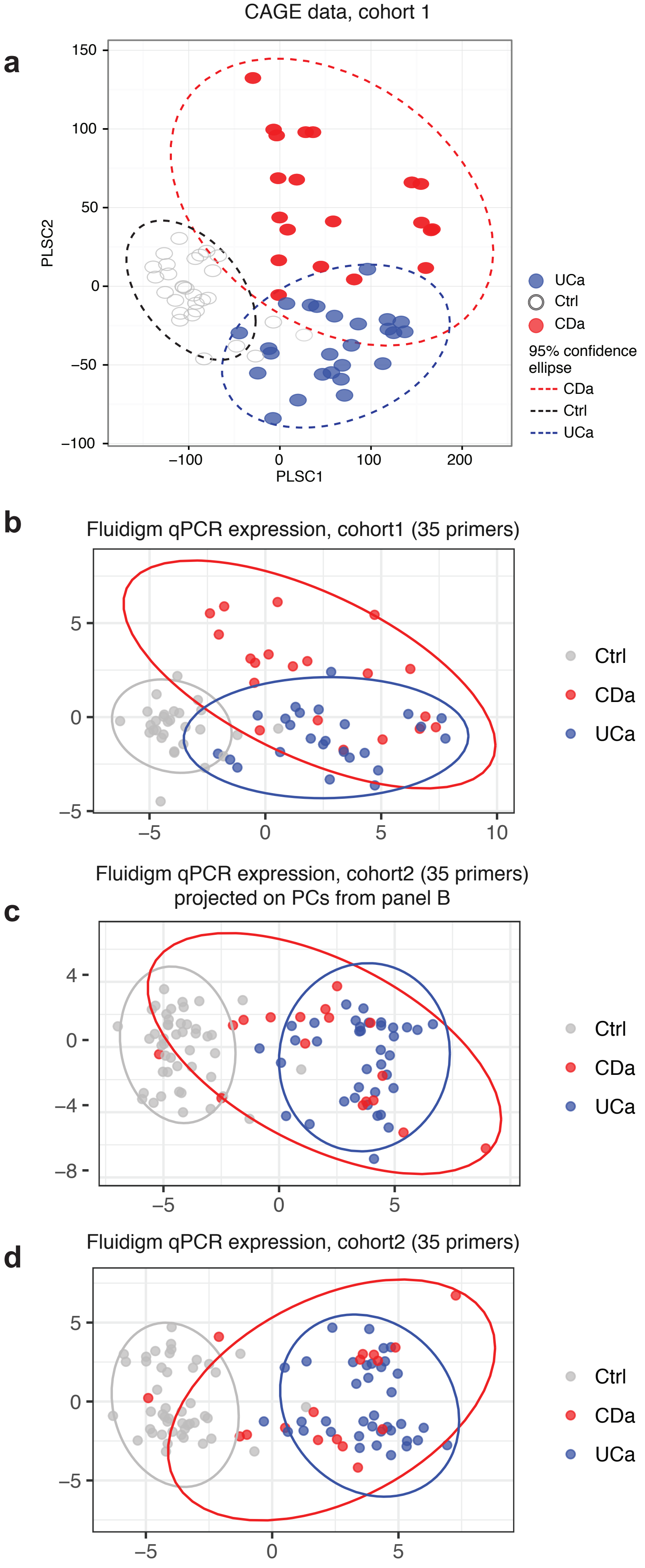

a) Separation of subject groups by PLSDA
Two-dimensional Partial Least Squares Discriminant Analysis (PLSDA) plot of CAGE TSS expression after ComBat correction. First and second components are shown on X- and Y-axes, respectively. Dashed lines indicate 95% confidence ellipses around each group. The CDa group shows the highest intra-group spread, followed by UCa and Ctrl.

b) PCA-plot of cohort 1 samples based on expression of 35 primers measured by fluidigm.

c) Samples from cohort 2 projected onto PCs from B.

d) PCA-plot of cohort 2 samples based on expression of 35 primers measured by fluidigm.

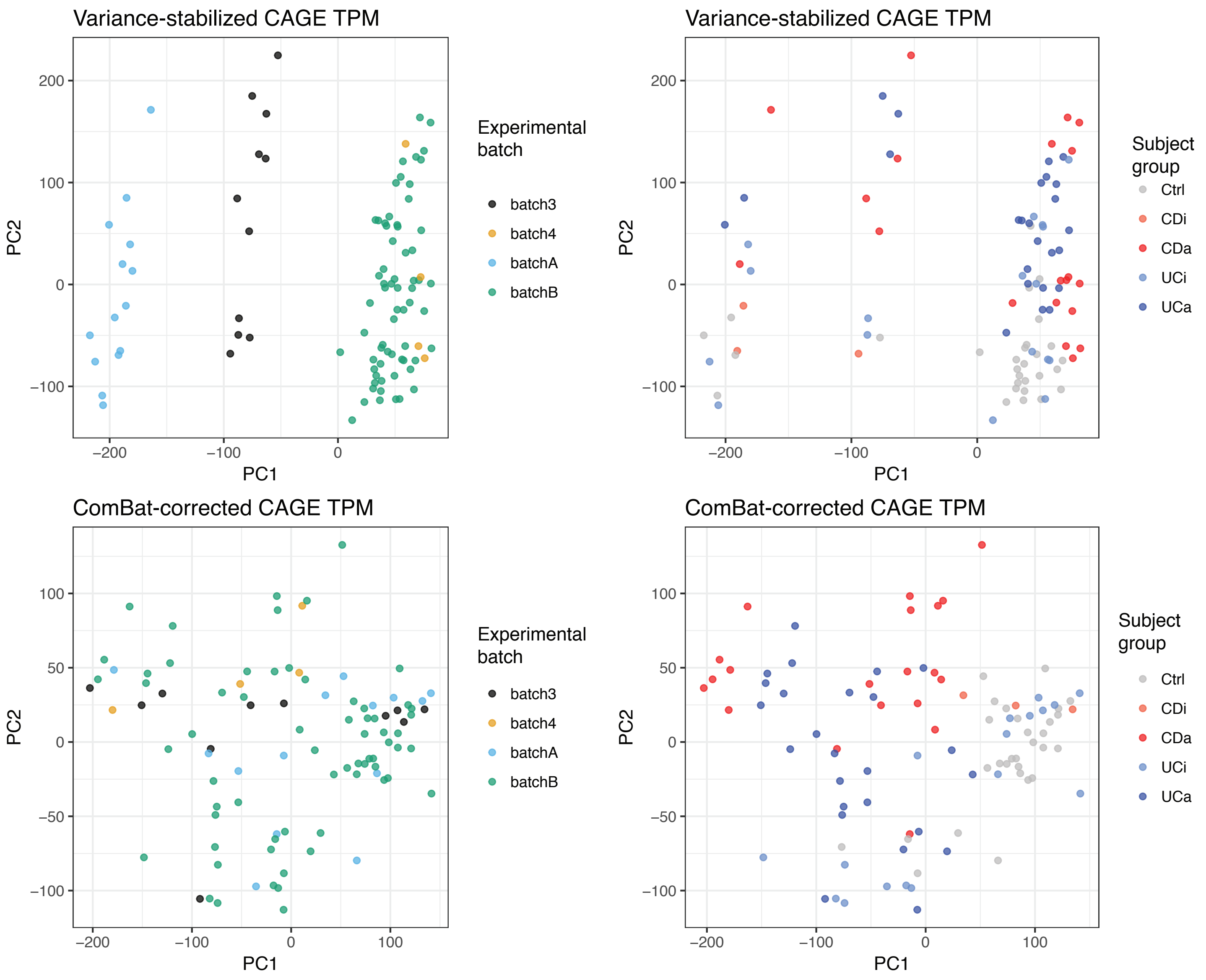

Correcting for experimental batch effects

Top row: two-dimensional PCA-plots of variance stabilized TSS expression (CAGE data), colored by experimental batch (left) and sample group (right). Samples separate by batch along the first component, and group along the second component. Batch labels are as in Supplementary data 1. Bottom row: two-dimensional PCA-plots of TSS expression after ComBat batch correction, colored by experimental batch (left) and sample group (right). Samples separate by IBD along the first component, and CDa vs. UCa along the second component. Bottom right plot is identical to Fig.2a.
