# Supplementary material for: Characterization of the enhancer and promoter landscape of inflammatory bowel disease from human colon biopsies
Source: Nat Commun. 2018 Apr 25;9:1661. doi: 10.1038/s41467-018-03766-z (PMC5916929; doi:10.1038/s41467-018-03766-z)
Supplement: Supplementary file 2 — Description of Additional Supplementary Files [file 41467_2018_3766_MOESM2_ESM.pdf]

Description of Additional Supplementary Files

File Name: Supplementary Data 1
Description: List of all samples, their diagnosis, batch, barcode, GEO record and CTSS file names. For each patient all information from Supplementary Table 1 is included (age, HB_score, Mayo_score, gender, smoking, familial, severity, age at diagnosis, duration of disease, SASA, LASA, SSB, LSB, TNF, AZA, Antibiotics, MXT,6-MP, surgery) as well as information on biopsy location and endoscopical and histological diagnoses. The last column shows phenotypes for controls.

File Name: Supplementary Data 2
Description: CAGE library statistics, including mapping rates and amplification cycles.

File Name: Supplementary Data 3
Description: All CAGE tag clusters used in the paper. The information includes chromosome location (seqnames) and strand (strand), tag cluster start (start), end (end) and width (width), assigned name (id) and total expression (score column). Feat_start and feat_end denote the location of highest expression

File Name: Supplementary Data 4
Description: Average expression, log fold changes and FDR-corrected P-values for TSS-level differential expression analysis. See README sheet for detailed information.

File Name: Supplementary Data 5
Description: Gene-level summary of DTU, including combined P-value and number of individual TSSs changing usage. See README sheet for detailed information.

File Name: Supplementary Data 6
Description: Average expression, log fold changes and FDR-corrected p-values for Gene-level differential expression analysis. See README sheet for detailed information.

File Name: Supplementary Data 7
Description: Complete results for enrichment of various functional categories, including GO and KEGG, for all differential expression sets. See README sheet for detailed information.

File Name: Supplementary Data 8
Description: Complete results for FANTOM5 cell type enrichment for differential expression sets using two-sided Fisher's Exact Test, including odds-ratios, confidence intervals and p-values. See README sheet for detailed information.

File Name: Supplementary Data 9
Description: The table lists the ΔΔCq expression values for 35 biomarkers measured on TNF alpha exposed monocytes and organoids. Rows show experiment ID, sample IDs, and exposure for the individual samples. Columns show primer pair IDs corresponding to biomarkers (as in Table S4-5). Cell values correspond to ΔΔCq expression for a given primer pair and individual, based on two independent cDNA synthesis reactions. The ΔΔCq expression values were calculated as described in Supplemental methods.

File Name: Supplementary Data 10
Description: All permissive enhancers used in the paper. As well as enhancer start and end and names, the thickStart and thickEnd columns define mid position. The score column gives the maximum pooled expression of tags used for each bidirectional loci.

File Name: Supplementary Data 11
Description: All conservative enhancers used in the paper. As well as enhancer start and end and names, the thickStart and thickEnd columns define mid position. The score column gives the maximum pooled expression of tags used for each bidirectional loci.

File Name: Supplementary Data 12
Description: Complete results for enhancer differential expression analysis using EBSeq, including log FCs, posterior probabilities and MAP estimates. See README sheet for detailed information.

File Name: Supplementary Data 13
Description: Complete results for enhancer-TSS correlations, including distances, Pearson correlation coefficients and P-values. See README sheet for detailed information.

File Name: Supplementary Data 14
Description: Genomic locations and enhancer member(s) of enhancer clusters and enhancer singletons

File Name: Supplementary Data 15
Description: The table shows a detailed overview of our biomarker selection process from the first group to the final 35 biomarkers. Each row is a biomarker. The CAGE ID column shows the CAGE cluster of each biomarker. Type of tag cluster provides its location and gene name the associated gene where applicable. Origin gives information from which selection phase the biomarkers originated: differential expression lists, manual curation, or reference genes. Next column describes either from which differential expression comparison the biomarker came from or the reasoning inclusion if it was done from manual selection manual selection. The following three columns shows for each marker if it made it through the selection process: passing manual curation phase, quality control phase and feature reduction phase. Finally, the last column shows background reasoning for feature selection if it was manual.

File Name: Supplementary Data 16
Description: qPCR primers used for standard and Fluidigm qPCR analysis. Primers used for CAGE tag clusters, eRNAs on both strands and reference genes are shown. Genomic locations refer to the hg19 assembly and indicate the position of the corresponding CAGE signal.

File Name: Supplementary Data 17
Description: The table lists the ΔΔCq expression values for the individual subjects and the 161 biomarkers measured on cohort 1. Rows show subject IDs. Columns show primer pair IDs corresponding to biomarkers (as in Table S4-5). Cell values correspond to ΔΔCq expression for a given primer pair and individual, based on two independent cDNA synthesis reactions. The ΔΔCq expression values were calculated as described in Supplemental methods.

File Name: Supplementary Data 18
Description: The table lists the batch corrected ΔΔCq expression values for the individual subjects and the 35 biomarkers measured on cohort 1 and 2. Rows show subject IDs. Columns show primer

pair IDs corresponding to biomarkers (as in Table S4-5). Cell values correspond to ΔΔCq expression for a given primer pair and individual, based on two independent cDNA synthesis reactions. The batch corrected ΔΔCq expression values were calculated as described in Supplemental methods.
